# Electronic Patient-Reported Outcome Monitoring to Improve Quality of Life After Joint Replacement: Secondary Analysis of a Randomized Clinical Trial

**DOI:** 10.1001/jamanetworkopen.2023.31301

**Published:** 2023-09-01

**Authors:** Viktoria Steinbeck, Benedikt Langenberger, Lukas Schöner, Laura Wittich, Wolfgang Klauser, Martin Mayer, David Kuklinski, Justus Vogel, Alexander Geissler, Christoph Pross, Reinhard Busse

**Affiliations:** 1 Department of Healthcare Management, School of Economics and Management, Technical University Berlin, Berlin, Germany; 2 Department of Orthopedics, VAMED Ostseeklinik Damp, Damp, Germany; 3 Chair of Healthcare Management, School of Medicine, University of St Gallen, St Gallen, Switzerland

## Abstract

**Importance:**

Although remote patient-reported outcome measure (PROM) monitoring has shown promising results in cancer care, there is a lack of research on PROM monitoring in orthopedics.

**Objective:**

To determine whether PROM monitoring can improve health outcomes for patients with joint replacement compared with the standard of care.

**Design, Setting, and Participants:**

A 2-group, patient-level randomized clinical trial (PROMoting Quality) across 9 German hospitals recruited patients aged 18 years or older with primary hip or knee replacement from October 1, 2019, to December 31, 2020, with follow-up until March 31, 2022.

**Interventions:**

Intervention and control groups received the standard of care and PROMs at hospital admission, discharge, and 12 months after surgery. In addition, the intervention group received PROMs at 1, 3, and 6 months after surgery. Based on prespecified PROM score thresholds, at these times, an automated alert signaled critical recovery paths to hospital study nurses. On notification, study nurses contacted patients and referred them to their physicians if necessary.

**Main Outcomes and Measures:**

The prespecified outcomes were the mean change in PROM scores (European Quality of Life 5-Dimension 5-Level version [EQ-5D-5L; range, −0.661 to 1.0, with higher values indicating higher levels of health-related quality of life (HRQOL)], European Quality of Life Visual Analogue Scale [EQ-VAS; range, 0-100, with higher values indicating higher levels of HRQOL], Hip Disability and Osteoarthritis Outcome Score–Physical Function Shortform [HOOS-PS; range, 0-100, with lower values indicating lower physical impairment] or Knee Injury and Osteoarthritis Outcome Score–Physical Function Shortform [KOOS-PS; range, 0-100, with lower values indicating lower physical impairment], Patient-Reported Outcomes Measurement Information System [PROMIS]–fatigue [range, 33.7-75.8, with lower values indicating lower levels of fatigue], and PROMIS-depression [range, 41-79.4, with lower values indicating lower levels of depression]) from baseline to 12 months after surgery. Analysis was on an intention-to-treat basis.

**Results:**

The study included 3697 patients with hip replacement (mean [SD] age, 65.8 [10.6] years; 2065 women [55.9%]) and 3110 patients with knee replacement (mean [SD] age, 66.0 [9.2] years; 1669 women [53.7%]). Exploratory analyses showed significantly better health outcomes in the intervention group on all PROMs except the EQ-5D-5L among patients with hip replacement, with a 2.10-point increase on the EQ-VAS in the intervention group compared with the control group (HOOS-PS, −1.86 points; PROMIS-fatigue, −0.69 points; PROMIS-depression, −0.57 points). Patients in the intervention group with knee replacement had a 1.24-point increase on the EQ-VAS, as well as significantly better scores on the KOOS-PS (−0.99 points) and PROMIS-fatigue (−0.84 points) compared with the control group. Mixed-effect models showed a significant difference in improvement on the EQ-VAS (hip replacement: effect estimate [EE], 1.66 [95% CI, 0.58-2.74]; knee replacement: EE, 1.71 [95% CI, 0.53-2.90]) and PROMIS-fatigue (hip replacement: EE, −0.65 [95% CI, −1.12 to −0.18]; knee replacement: EE, −0.71 [95% CI, −1.23 to −0.20]). The PROMIS-depression score was significantly reduced in the hip replacement group (EE, −0.60 [95% CI, −1.01 to −0.18]).

**Conclusions and Relevance:**

In this randomized clinical trial, the PROM-based monitoring intervention led to a small improvement in HRQOL and fatigue among patients with hip or knee replacement, as well as in depression among patients with hip replacement.

**Trial registration:**

Deutsches Register Klinischer Studien ID: DRKS00019916

## Key Points

**Question:**

Does patient-reported outcome measure (PROM)–based remote monitoring after hip and knee replacement surgery improve health outcomes compared with the standard of care?

**Findings:**

This multicenter randomized clinical trial included 3697 patients with primary hip replacement and 3110 with knee replacement across 9 German hospitals. The intervention group had a statistically significantly higher mean increase from baseline to 12 months after surgery in health-related quality of life score, fatigue score, and depression score compared with the control group.

**Meaning:**

This randomized clinical trial found that postoperative PROM monitoring, including alerts for critical recovery, led to small improvements in several health dimensions (health-related quality of life and fatigue after hip and knee replacement and depression after hip replacement).

## Teaser

This randomized clinical trial evaluates whether patient-reported outcome measure monitoring can improve health outcomes for patients with joint replacement compared with the standard of care.

## Introduction

Patient-reported outcome measures (PROMs) are increasingly used for research and health technology assessment across indications and geographic regions^1^; however, their value in routine care and remote patient monitoring is still under investigation.^2^ At the same time, digital technologies are advancing reporting and integration of PROMs into real-time care analytics.^3^ Specifically, PROMs can be used for remote monitoring and early detection of critical recovery pathways.

Critical recovery detection via PROMs has shown promising results in oncology, leading to higher survival rates, improved health-related quality of life (HRQOL), and reduced hospital visits.^4,5,6^ However, there is limited evidence from the field of orthopedics. Some studies show that postsurgery PROM scores are associated with early revisions.^7,8^ In many countries, joint replacements are common procedures that lack a standardized postsurgery pathway with regular consultations, bearing the risk of inadvertent postsurgery adverse effects remaining undetected.^9^ This lack of follow-up is exacerbated in the German health care system, where inpatient care and outpatient care are separate care silos, hindering seamless follow-up care.^10^ Simultaneously, identifying and responding earlier to suboptimal outcome developments can improve patients’ well-being and recovery time.^11^

As quality variation persists and current outcome measures only partially reflect relevant outcomes after surgery,^12^ the Centers for Medicare & Medicaid Services in the US are mandating patient-reported outcome reporting for inpatient hip and knee replacement in a phased implementation from 2023 onward.^13^ Simultaneously, some countries have been collecting PROMs in joint replacement care for years, and others are continuously implementing the collection of PROMs in this setting.^14,15,16^ The increased use of PROMs, the established PROM standard sets for orthopedics, digital PROM platforms, and the lack of standardization of postsurgery follow-up make hip replacement and knee replacement an ideal case for remote monitoring and critical recovery detection based on PROMs that can prompt early review and, if needed, appropriate interventions. Hence, we aimed to determine whether PROM-based remote monitoring after hip replacement or knee replacement surgery improves health outcomes compared with the standard of care.

## Methods

### Study Design

A 2-group, parallel patient-level randomized clinical trial, PROMoting Quality, was conducted across 9 German hospitals to assess a PROM-based monitoring intervention for patients with hip replacement or knee replacement. This study presents an a priori–specified analysis of the secondary outcomes. Patients were blinded to group assignment and group allocation sequence, which was digitally initiated at discharge (individual randomization) and concealed from patients, study nurses, and physicians. The study was conducted in line with the published study protocol^17^ and analysis plan (Supplement 1). The study’s patient recruitment timeframe was prolonged due to the COVID-19 pandemic, and minimal adjustments were made as indicated in the trial registration and analysis plan (eTable 1 in Supplement 2). Patients were recruited from October 1, 2019, to December 31, 2020, with follow-up until March 31, 2022. The PROMoting Quality study was approved by the ethics committee of the Charité-Universitätsmedizin, Berlin, and registered with the German Register for Clinical Studies. Written informed consent was obtained from all participating patients, which could be revoked at any time until anonymization.^17^ The reporting in this article followed the Consolidated Standards of Reporting Trials Extension (CONSORT PRO Extension) reporting guideline.^18^

Patients digitally received the German language versions of (1) the Hip Disability and Osteoarthritis Outcome Score–Physical Function Shortform (HOOS-PS) and Knee Injury and Osteoarthritis Outcome Score–Physical Function Shortform (KOOS-PS) to measure physical functioning, scored from 0 to 100, with lower values indicating lower physical impairment^19^; (2) the European Quality of Life 5-Dimension 5-Level version (EQ-5D-5L) and European Quality of Life Visual Analogue Scale (EQ-VAS) to measure HRQOL, scored from −0.661 to 1.0 (using the German value set^20^) and 0 to 100, respectively, with higher values indicating higher levels of HRQOL (due to the low score range of the EQ-5D-5L, 3 digits after the decimal point are reported)^21,22^; and (3) the Patient-Reported Outcomes Measurement Information System (PROMIS)–fatigue and PROMIS-depression, version 1.0 4a, to measure specific physical and mental health domains, scored from 33.7 to 75.8 and 41 to 79.4, respectively, with lower values indicating lower levels of fatigue and depression symptoms.^23^

All PROMs are validated in German, and most PROMs are validated specifically for patients with hip replacement or knee replacement.^19,21,22,23,24,25,26^ Most PROMs are recommended by the International Consortium for Health Outcomes Measurement.^27^

The control group received PROMs before surgery (between admission to the hospital and surgery), at discharge, and 12 months after surgery, whereas the intervention group additionally received PROMs at 1, 3, and 6 months after surgery (step 1). Automated digital alerts signaled critical recovery paths to hospital study nurses at 1, 3, and 6 months after surgery via a digital notification in the study software of their work computer (step 2).^17^ There were 2 ways in which an alert was initiated: first, by surpassing predetermined PROM thresholds (eTable 2 in Supplement 2) and, second, when a 10% relative worsening of a patient’s individual PROM score was detected (on the EQ-5D-5L, HOOS-PS, or KOOS-PS). After an alert, study nurses contacted patients (step 3) and referred the alert and PROM information to patients and/or their inpatient or outpatient physicians if deemed necessary by the patients (step 4). Patients and physicians were then free to use this information in their preferred way.

### Hospitals

The 9 participating hospitals included 1 university hospital, 1 nonteaching hospital, and 7 teaching hospitals. In Germany, the standard of care includes postsurgery rehabilitation in an inpatient or ambulatory care setting for 3 weeks. Five of the participating hospitals offer inpatient rehabilitation, but where patients receive rehabilitation depends on their health insurance. Both intervention and control group patients equally received the German preoperative and postoperative standard of care. Yearly case volumes per hospital in 2020 ranged from 348 to 2137 (mean, 1051) for hip replacement and from 284 to 1982 (mean, 952) for knee replacement.^28^

### Patients

**Figure 1.**
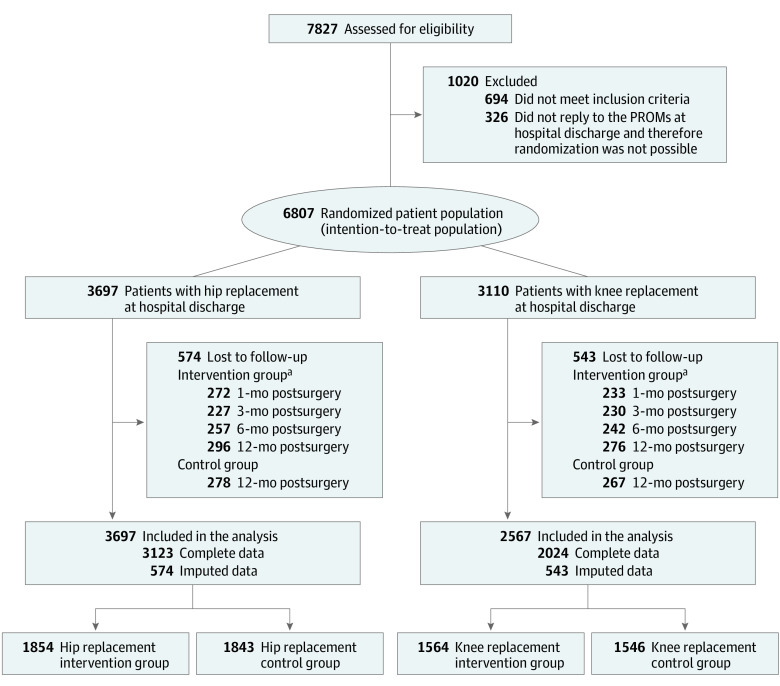
Participant Flow in the PROMoting Quality Trial Under the Intention-to-Treat Assumption PROM indicates patient-reported outcome measure. ^a^Participants in the intervention group were also allowed to skip 1 follow-up (eg, if they did not reply in month 3, they could still reply in month 6). Hence, there are missing data for these times, but they were not completely lost to follow-up.

Study nurses and/or physicians aimed to include all eligible patients in the trial. Patients 18 years of age or older with prespecified surgery codes for hip replacement and knee replacement were included. Exclusion criteria were emergency and life-threatening cases, tumor prothesis, American Society of Anesthesiologists classifications 4 to 6, and patients without email access or without a relative supporting the PROM response. Randomization was triggered automatically at hospital discharge via the PROM-IT-software. A total of 6807 patients were included in the final analysis (Figure 1; eFigure 1 in Supplement 2). Missing data among the study participants were imputed using a random forest imputation with the missForest package, version 1.5 in R, version 4.1.3 (R Group for Statistical Computing). Race and ethnicity were not assessed due to strict rules on assessing these variables in Germany. The study refers to gender because the included variable refers to self-perceived gender identity.

### Outcomes

Although the primary outcome of the PROMoting Quality trial was the cost-effectiveness of the intervention using a composite of all PROM scores, the primary outcomes of this secondary analysis are the trial’s secondary outcomes (ie, changes in individual PROM scores from baseline to 12 months after surgery).^17^ These 5 outcomes are the changes in scores on the (1) EQ-5D-5L, (2) EQ-VAS, (3) HOOS-PS or KOOS-PS, (4) PROMIS-fatigue, and (5) PROMIS-depression. The cost-effectiveness analysis will be published later because health insurance regulations delayed cost data retrieval and processing.

### Statistical Analysis

Descriptive analyses were run per group and joint replacement type for the patient characteristics, PROM scores, and clinical characteristics. Analyses were separated per joint replacement type due to their different recovery durations and levels.^29,30^ As exploratory analyses, 2-sided *t* tests were performed to assess for significant differences between the intervention and control groups for all outcomes using *P* < .05 as the statistical significance threshold. For the main analysis, a linear mixed-effect model was used in the intention-to-treat study population. This model controls for age, gender, mobilization, and presurgery PROM score as fixed covariates and hospital as the random intercept to account for a potential clustering effect at the hospital level. The use of a mixed-effect model was based on a study by Twisk et al,^31^ which showed the benefits of controlling for covariates in randomized clinical trials.

As sensitivity analyses, the primary outcomes were assessed in a compliance-corrected sample^32^ including intervention group patients who completed their PROMs for at least 2 intervention time points. The number of initiated alerts based on critical PROM scores and reactions to alerts from study nurses and patients were analyzed to contextualize the results.

## Results

### Patient Characteristics

Overall, 1854 patients with hip replacement (mean [SD] age, 65.9 [10.6] years; 1029 women [55.5%]) and 1564 patients with knee replacement (mean [SD] age, 66.1 [9.1] years; 839 women [53.6%]) were assigned to the intervention group, and 1843 patients with hip replacement (mean [SD] age, 65.7 [10.6] years; 1036 women [56.2%]) and 1546 patients with knee replacement (mean [SD] age, 65.9 [9.4] years; 830 women [53.7%]) were assigned to the control group (Table 1). The largest share of patients with hip replacement was overweight (1421 of 3697 [38.4%]), while the largest share of patients with knee replacement was obese (1443 of 3110 [46.4%]). Most patients had a high school degree (3977 of 6807 [58.4%]) and were voluntarily not employed, including those in retirement (3889 of 6807 [57.1%]). The largest share of patients in both joint replacement groups (hip replacement, 1708 of 3697 [46.2%]; knee replacement, 1425 of 3110 [45.8%]) started mobilization within 6 hours after surgery (eTable 3 in Supplement 2 for the compliance-corrected descriptives).

**Table 1.** Characteristics of the Study Population (Intention-to-Treat Population)

Characteristic	Patients with hip replacement		Patients with knee replacement	
	Intervention (n = 1854)	Control (n = 1843)	Intervention (n = 1564)	Control (n = 1546)
Age, mean (SD), y	65.9 (10.6)	65.7 (10.6)	66.1 (9.1)	65.9 (9.4)
Gender, No. (%)				
Female	1029 (55.5)	1036 (56.2)	839 (53.6)	830 (53.7)
Male	825 (44.5)	807 (43.8)	725 (46.4)	716 (46.3)
BMI group, No. (%)				
Underweight (<18.5)	10 (0.5)	10 (0.5)	3 (0.2)	3 (0.2)
Normal (18.5-24.9)	576 (31.1)	588 (31.9)	255 (16.3)	222 (14.4)
Overweight (25.0-29.9)	722 (38.9)	699 (37.9)	566 (36.2)	618 (40.0)
Obese (≥30.0)	546 (29.4)	546 (29.6)	740 (47.3)	703 (45.5)
Current smoker, No. (%)				
No	1586 (85.5)	1547 (83.9)	1369 (87.5)	1336 (86.4)
Yes	268 (14.5)	296 (16.1)	195 (12.5)	210 (13.6)
Educational level, No. (%)				
No school degree	8 (0.4)	7 (0.4)	13 (0.8)	5 (0.3)
Primary school degree	243 (13.1)	249 (13.5)	263 (16.8)	271 (17.5)
High school or middle school degree	1085 (58.5)	1033 (56.1)	932 (59.6)	927 (60.0)
University degree	518 (27.9)	554 (30.1)	356 (22.8)	343 (22.2)
Living situation, No. (%)				
Alone	430 (23.2)	414 (22.5)	310 (19.8)	289 (18.7)
Care facility	4 (0.2)	7 (0.4)	10 (0.6)	9 (0.6)
With a partner, family, or friends	1411 (76.1)	1408 (76.4)	1239 (79.2)	1233 (79.8)
Other	9 (0.5)	14 (0.8)	5 (0.3)	15 (1.0)
Job, No. (%)				
Working	583 (31.4)	618 (33.5)	454 (29.0)	468 (30.3)
Voluntarily not working including retirement	1065 (57.4)	1034 (56.1)	915 (58.5)	875 (56.6)
Looking for work	20 (1.1)	15 (0.8)	11 (0.7)	23 (1.5)
Unable to work	186 (10.0)	176 (9.6)	184 (11.8)	180 (11.6)
Mobilization after surgery, No. (%)				
Within 6 h	854 (46.1)	854 (46.3)	716 (45.8)	709 (45.9)
Within 12 h	560 (30.2)	496 (26.9)	460 (29.4)	425 (27.5)
Within 24 h	403 (21.7)	443 (24.0)	331 (21.2)	356 (23.0)
Within 48 h	26 (1.4)	35 (1.9)	43 (2.7)	46 (3.0)
After 48 h	11 (0.6)	15 (0.8)	14 (0.9)	10 (0.6)
Readmission, No. (%)				
No	1809 (97.6)	1789 (97.1)	1531 (97.9)	1506 (97.4)
Within 30 d after surgery	19 (1.0)	28 (1.5)	9 (0.6)	21 (1.4)
30-90 d After surgery	26 (1.4)	26 (1.4)	24 (1.5)	19 (1.2)
Reoperation within 12 mo after surgery, No. (%)				
No	1816 (98.0)	1798 (97.6)	1530 (97.8)	1504 (97.3)
Yes	38 (2.0)	45 (2.4)	34 (2.2)	42 (2.7)
PROM baseline score, mean (SD)^a^				
EQ-5D-5L	0.594 (0.263)	0.603 (0.256)	0.625 (0.257)	0.623 (0.245)
EQ-VAS	56.5 (20.0)	57.1 (19.7)	58.7 (19.4)	57.9 (19.1)
HOOS-PS or KOOS-PS	48.4 (16.4)	47.1 (16.1)	43.1 (13.3)	43.0 (12.0)
PROMIS-fatigue	49.2 (9.9)	49.2 (10.0)	48.3 (10.1)	48.1 (9.5)
PROMIS-depression	49.7 (8.4)	49.8 (8.3)	49.4 (8.4)	49.4 (8.2)

Abbreviations: BMI, body mass index (calculated as weight in kilograms divided by height in meters squared); EQ-5D-5L, European Quality of Life 5-Dimension 5-Level version; EQ-VAS, European Quality of Life Visual Analogue Scale; HOOS-PS, Hip Disability and Osteoarthritis Outcome Score–Physical Function Shortform; KOOS-PS, Knee Injury and Osteoarthritis Outcome Score–Physical Function Shortform; PROM, patient-reported outcome measure; PROMIS, Patient-Reported Outcomes Measurement Information System.

^a^ PROM scores have different score ranges: −0.661 to 1.0 for the EQ-5D-5L, 0 to 100 for the EQ-VAS, 0 to 100 for the HOOS-PS and KOOS-PS, 33.7 to 75.8 for the PROMIS-fatigue, and 41 to 79.4 for the PROMIS-depression. Higher values on the EQ-VAS and EQ-5D-5L indicate better health levels, whereas lower values on the HOOS-PS, KOOS-PS, PROMIS-fatigue, and PROMIS-depression indicate better health (less health impairment). Due to the low score range of the EQ-5D-5L, 3 digits after the decimal point are reported.

### Outcomes Over Time

**Figure 2.**
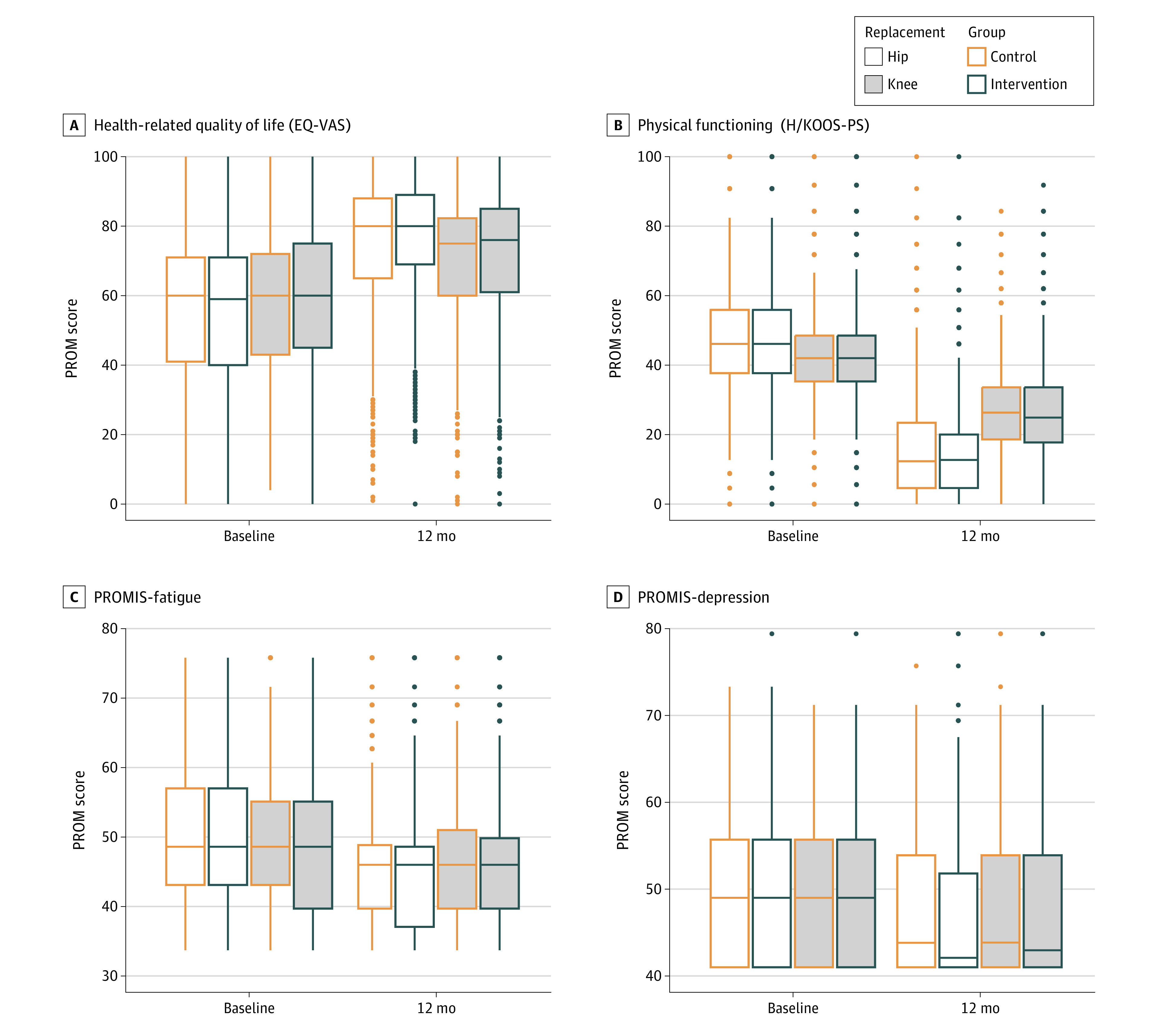
Distriubtion of Raw Patient-Reported Outcome Measure (PROM) Scores for the Control and Intervention Groups Higher values on the European Quality of Life Visual Analogue Scale (EQ-VAS) indicate better health levels, whereas lower values in physical functioning (Hip Disability and Osteoarthritis Outcome Score–Physical Function Shortform or Knee Injury and Osteoarthritis Outcome Score–Physical Function Shortform [H/KOOS-PS]), Patient-Reported Outcomes Measurement Information System (PROMIS)–fatigue, and PROMIS-depression indicate better health (less health impairment). The box indicates the middle 50% of scores for the respective sample (the IQR). The line that divides the box into 2 parts indicates the median.The whiskers extend from each box to capture the range of the remaining data within ±1.5 times the IQR. Any score beyond that distance is considered an outlier and is indicated as a circle.

Figure 2 shows the positive health change based on the raw PROM scores at baseline and 12 months after surgery in the intervention and control groups (eTable 4 and eFigure 2 in Supplement 2). The positive health change was less pronounced for patients with knee replacement compared with patients with hip replacement. Readmission and reoperation rates were compared between groups but showed low numbers that did not indicate significant differences between groups (eTable 5 in Supplement 2).

### Compliance in Answering the PROM Questionnaires

In the intervention group, response rates to the PROM questionnaires were at 85.3% (1582 of 1854), 87.8% (1627 of 1854), and 86.1% (1597 of 1854) at intervention time points at 1, 3, and 6 months after surgery, respectively, for patients with hip replacement and 85.1% (1331 of 1564), 85.3% (1334 of 1564), and 84.5% (1322 of 1564) at 1, 3, and 6 months after surgery, respectively, for patients with knee replacement. At 12 months after surgery, after the intervention timeframe, compliance was 84.0% (1558 of 1854) in the hip replacement intervention group, 84.9% (1565 of 1843) in the hip replacement control group, 82.4% (1288 of 1564) in the knee replacement intervention group and 82.7% (1279 of 1546) in the knee replacement control group.

### Alerts Based on Critical PROM Scores and Reactions to Alerts

The percentage of alerts based on critical PROM scores as a share of the patients with hip replacement with available information on the critical values was 8.9% (157 of 1772) at 1 month, 25.6% (454 of 1773) at 3 months, and 24.8% (424 of 1709) at 6 months (eTable 6 in Supplement 2). For patients with knee replacement, the percentage of alerts based on critical PROM scores was 10.6% (157 of 1486) at 1 month, 26.0% (386 of 1485) at 3 months, and 32.1% (461 of 1435) at 6 months. Among the patients whose PROM scores initiated alerts, 529 initiated an alert once, 188 initiated an alert twice, and 41 initiated an alert 3 times (eFigures 3 and 4 in Supplement 2). Around 20% of alerts based on critical PROM scores were followed up by an email or postal transfer of PROM information to patients (hip replacement: 18.5% [29 of 157] at 1 month, 19.4% [88 of 454] at 3 months, 19.6% [83 of 424] at 6 months; knee replacement: 20.4% [32 of 157] at 1 month, 19.2% [74 of 386] at 3 months, 22.8% [105 of 461] at 6 months) (eTable 6 in Supplement 2). Around 70% of alerts were followed up by a call from study nurses to patients (hip replacement: 72.6% [114 of 157] at 1 month, 70.3% [319 of 454] at 3 months, 75.7% [321 of 424] at 6 months; knee replacement: 70.7% [111 of 157] at 1 month, 74.4% [287 of 386] at 3 months, 68.5% [316 of 461] at 6 months). In 21.1% (24 of 114), 13.5% (43 of 319), and 8.1% (26 of 321) of nurse calls to patients at 1, 3, and 6 months, respectively, patients with hip replacement agreed that they wanted their PROM data to be transferred to a physician by the study nurse (knee replacement: 25.2% [28 of 111], 17.1% [49 of 287], 7.9% [25 of 316] at 1, 3, and 6 months, respectively).

The proportion of treatment changes among patients who visited a physician in the quarter after the PROMs were asked was higher in the group of patients who were called vs those who were not called concerning some changes (eg, change in the aftercare physician at month 3 for patients with hip replacement: 9.8% [8 of 82] vs 2.5% [24 of 960]) but lower concerning other changes (eg, change in physiotherapy at month 3 for patients with hip replacement: 37.8% [31 of 82] vs 42.4% [407 of 960]) (eTable 7 in Supplement 2).

With the study protocol prescribing every patient with an alert to be called, a subanalysis of study nurses’ notes (eFigure 5 in Supplement 2) showed why calls did not take place. For 47.3% (264 of 558), no notes were available, and for the second-largest share (21.7% [121 of 558]), study nurses indicated that they perceived the PROM score difference as too small for a call to be sensible.

### Exploratory Analysis: Mean PROM Score Change

Table 2 shows the mean change in PROM scores from baseline to 12 months after surgery and the mean between-group differences. All mean changes indicated higher health improvements in the intervention group. No significant difference was found between the intervention and control groups in the mean (SD) change in EQ-5D-5L score (hip replacement: 0.287 [0.260] vs 0.271 [0.262]; difference, 0.016 [95% CI, –0.000 to 0.033]; knee replacement: 0.227 [0.255] vs 0.220 [0.251]; difference, 0.007 [95% CI, –0.011 to 0.025]). A significant difference between the intervention and control groups was reported in the mean (SD) change in the EQ-VAS score for patients with hip replacement (18.98 [22.06] vs 16.88 [22.56]; difference, 2.10 [95% CI, 0.66-3.54]) but not for patients with knee replacement (13.55 [21.60] vs 12.31 [21.27]; difference, 1.24 [95% CI, –0.27 to 2.74]). A significantly higher mean (SD) change for the intervention vs control groups was also found in the HOOS-PS and KOOS-PS scores (HOOS-PS: −33.79 [17.41] vs −31.93 [17.99]; difference, −1.86 [95% CI, –3.01 to –0.72]; and KOOS-PS: −17.35 [14.31] vs −16.36 [13.60]; difference, −0.99 [95% CI, −1.97 to 0.01]), while PROMIS-depression showed significant differences between the intervention and control groups only among patients with hip replacement (hip replacement: −2.78 [7.69] vs −2.21 [7.89]; difference, −0.57 [95% CI, −1.07 to −0.06]; knee replacement: −1.88 [7.62] vs −1.64 [7.71]; difference, −0.24 [95% CI, −0.78 to 0.29]). Fatigue change was significantly different between the intervention and control groups for both surgery types (hip replacement: −4.32 [9.21] vs −3.63 [9.17]; difference, −0.69 [95% CI, −1.29 to −0.11]; knee replacement: −2.72 [9.09] vs −1.88 [8.81]; difference, −0.84 [95% CI, −1.48 to −0.22]). The compliance-corrected analysis showed similar results in terms of significantly different PROM score changes, including a difference of −1.85 for the HOOS-PS among the hip replacement group and a difference of −1.14 for the KOOS-PS among the knee replacement group (eTable 8 in Supplement 2).

**Table 2.** Difference in the PROM Score Change From Baseline to 12 Months After Surgery Between Intervention and Control Group

PROM	Intervention		Control		Mean between-group difference (95% CI)^b^	*P* value^c^
	No.	Mean (SD) change in PROM score^a^	No.	Mean (SD) change in PROM score^a^		
Patients with hip replacement						
EQ-5D-5L	1854	0.287 (0.260)	1843	0.271 (0.262)	0.016 (−0.000 to 0.033)	.06^d^
EQ-VAS	1854	18.98 (22.06)	1843	16.88 (22.56)	2.10 (0.66 to 3.54)	.004^e^
HOOS-PS	1854	−33.79 (17.41)	1843	−31.93 (17.99)	−1.86 (−3.01 to −0.72)	.001^e^
PROMIS-depression	1854	−2.78 (7.69)	1843	−2.21 (7.89)	−0.57 (−1.07 to −0.06)	.03^f^
PROMIS-fatigue	1854	−4.32 (9.21)	1843	−3.63 (9.17)	−0.69 (−1.29 to −0.11)	.02^f^
Patients with knee replacement						
EQ-5D-5L	1564	0.227 (0.255)	1546	0.220 (0.251)	0.007 (−0.011 to 0.025)	.44
EQ-VAS	1564	13.55 (21.60)	1546	12.31 (21.27)	1.24 (−0.27 to 2.74)	.11
KOOS-PS	1564	−17.35 (14.31)	1546	−16.36 (13.60)	−0.99 (−1.97 to 0.01)	.048^f^
PROMIS-depression	1564	−1.88 (7.62)	1546	−1.64 (7.71)	−0.24 (−0.78 to 0.29)	.37
PROMIS-fatigue	1564	−2.72 (9.09)	1546	−1.88 (8.81)	−0.84 (−1.48 to −0.22)	.008^e^

Abbreviations: EQ-5D-5L, European Quality of Life 5-Dimension 5-Level version; EQ-VAS, European Quality of Life Visual Analogue Scale; HOOS-PS, Hip Disability and Osteoarthritis Outcome Score–Physical Function Shortform; KOOS-PS, Knee Injury and Osteoarthritis Outcome Score–Physical Function Shortform; PROM, patient-reported outcome measure; PROMIS, Patient-Reported Outcomes Measurement Information System.

^a^ PROM scores have different ranges: −0.661 to 1.0 for the EQ-5D-5L, 0 to 100 for the EQ-VAS, 0 to 100 for the HOOS-PS and KOOS-PS, 33.7 to 75.8 for the PROMIS-fatigue, and 41 to 79.4 for the PROMIS-depression. Due to the low score range of the EQ-5D-5L, 3 digits after the decimal point are reported.

^b^ The mean difference refers to the difference between intervention and control groups in terms of their mean change in the respective PROM score.

^c^ Based on independent sample *t* test.

^d^ *P* < .01.

^e^ *P* < .05.

^f^ *P* < .10.

### Main Analysis of the Primary Outcome: Mixed-Effect Models

After controlling for patient and treatment attributes in the mixed-effect models, the intervention showed significant effect estimates (EEs) for the changes in EQ-VAS score (EE, 1.66 [95% CI, 0.58-2.74]), PROMIS-fatigue score (EE, −0.65 [95% CI, −1.12 to −0.18]), and PROMIS-depression score (EE, −0.60 [95% CI, −1.01 to −0.18]) for patients with hip replacement (eTable 9 in Supplement 2). For patients with knee replacement, the EEs for the EQ-VAS (EE, 1.71 [95% CI, 0.53-2.90]) and PROMIS-fatigue (EE, −0.71 [95% CI, −1.23 to −0.20]) were significantly better for the intervention group. Similar results were found in the compliance-corrected analyses for the EQ-VAS and PROMIS-fatigue, while PROMIS-depression score changes were significant at the 10% level; the difference of –0.89 EE for the HOOS-PS among the hip replacement group and the difference of −0.94 EE for the KOOS-PS among the knee replacement group indicated significantly higher improvements in the intervention group (eTable 10 in Supplement 2).

**Figure 3.**
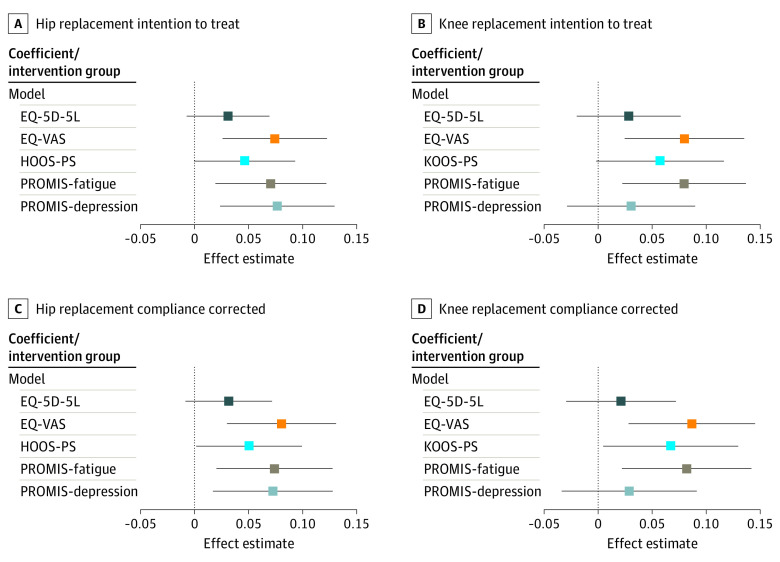
Mixed-Effect Model Effect Estimates for the Intervention per Patient-Reported Outcome Measure (PROM) Score The mixed-effect models used *z* scores as outcomes. The *z* scores were used as outcomes to standardize the different PROM scores on 1 scale and thereby facilitate the comparison of different PROM scores. In their raw form, the PROM scores are measured on different scales and would hence not be directly comparable. The compliance-corrected analysis excludes all patients who did not answer the PROMs at a minimum of 2 intervention time points in the intervention group; effect estimates above zero indicate better health changes in the intervention group, whereas estimates below zero indicate better health levels in the control group. EQ-5D-5L indicates European Quality of Life 5-Dimension 5-Level version; EQ-VAS, European Quality of Life Visual Analogue Scale; HOOS-PS, Hip Disability and Osteoarthritis Outcome Score–Physical Function Shortform; KOOS-PS, Knee Injury and Osteoarthritis Outcome Score–Physical Function Shortform; and PROMIS, Patient-Reported Outcomes Measurement Information System.

The results of the mixed-effect models are visualized in Figure 3 using *z* scores to make the PROM scores visually comparable on 1 scale. Positive EEs indicate better health in the intervention group (eTables 11 and 12 in Supplement 2).

## Discussion

Overall, this randomized clinical trial demonstrated small, statistically significant health benefits in HRQOL and fatigue using the remote PROM monitoring intervention compared with the standard of care for patients with hip replacement and patients with knee replacement; for example, patients with hip replacement had a mean 2.10 between-group difference and 1.66 EE on the EQ-VAS, and a −0.69 between-group difference and −0.65 EE on the PROMIS-fatigue. The intention-to-treat analyses and compliance-corrected analyses confirm these results. In addition, among patients with hip replacement, a small but significant difference in the PROMIS-depression score was observed, with a −0.57 between-group difference and −0.60 EE.

With regard to the clinical significance of the results, to our knowledge, there is currently no established standard for evaluating meaningful PROM score change differences between groups for all selected PROMs for patients with hip replacement or knee replacement. Considering current generic minimal important change (MIC) recommendations for PROMIS measures,^33,34^ the differences in PROMIS-fatigue and PROMIS-depression for patients with hip replacement and the difference in PROMIS-fatigue for patients with knee replacement are above the 2-point *t* value level and can thereby be interpreted as meaningfully different. Based on a MIC for the EQ-VAS for patients with knee replacement, the difference is below the suggested MIC of 5.27 points and could therefore be considered not meaningful.^35^ The EQ-VAS between-group point difference of 2.10 among patients with hip replacement, however, is higher than the 1-point difference observed between fast track vs non–fast track treated patients with hip replacement in the study by Berg et al.^36^ For patients with knee replacement, the observed 1.24 between-group difference on the EQ-VAS is lower than the 2-point difference observed by Berg et al.^36^ As a major health improvement is already achieved through joint replacement surgery, future MIC research should consider how the standard of care plus an add-on intervention, such as PROM-based monitoring, can be compared with the standard of care.

Although the mean change in physical functioning (HOOS-PS and KOOS-PS) was significantly different between the intervention and control groups before adjustment, the significant difference disappeared after adjustment. This could reflect the change in this health dimension being driven by variables for which the randomization might not have completely accounted (eg, presurgery PROM scores). However, a significant difference was observed in the compliance-corrected population, with a difference of −1.85 and an EE of –0.89 for the HOOS-PS among the hip replacement group and a difference of −1.14 and an EE of –0.94 for the KOOS-PS among the knee replacement group, warranting further analysis into the relationship between compliance, alert reactions, and functional health outcomes.

Due to the multistep nature of the intervention, it is unclear which aspect of the intervention caused the positive health changes. Whereas filling out the PROMs and, thus, patients reflecting more on their own health status might already have initiated change (step 1), change could have also been initiated by the reassuring idea that, if scores were critical, a health care professional would get in touch (step 2: alert to study nurses). The health changes might also have been triggered by calls or data transfers to patients by study nurses after the alerts (step 3), or by the actions based on the data transfer to physicians (step 4). Although around 70% of patients were informed about their critical values via a telephone call, around 20% received their values also or only per post or email (step 3). Given the low percentage of patients who were called and initiated PROM data transfers to physicians (hip replacement: 8.1%-21.1%; knee replacement: 7.9%-25.2%) (step 4), one could argue that earlier steps (monitoring, alerts, and calls) were more likely than the data transfer to physicians to drive the positive health changes. Considering the affected health dimensions, the results could point toward a “caring effect” through follow-up communication, rather than a “physical recovery effect” through in-person interventions. Virtual monitoring and telephone communication might have been especially relevant because the trial ran during the COVID-19 pandemic. Moreover, literature suggests that the completion of PROMs in itself can change the way in which patients think about their condition.^37^ These aspects justify further research into the relevance of virtual monitoring and follow-up telephone communication in health care for reducing fatigue and depression symptoms and improving HRQOL.

Based on the presented information, some aspects should be considered when implementing the intervention into routine care. First, a high response rate of around 85% was achieved, in comparison with registry-based PROM collection, showing a mean response rate of 42% for digital PROMs without a reminder.^38^ This response rate was supported through digital reminders and personal reminder calls from study nurses, reflecting that reminder options could be an essential element of the intervention. Second, study nurses did not call all patients who had critical alerts, with 21.7% indicating that the deterioration in patients’ health was too small for a call to be sensible. The adjustment of the relative alert thresholds based on study nurses’ feedback also supports this finding. This reflects the importance of setting accurate, possibly more patient-specific alert thresholds, which do not overburden study nurses while identifying patients with critical recovery needs. Third, implementing the intervention could benefit from a control mechanism for which all patients with critical values would be contacted (eg, central call center to mitigate staff shortage) or digital information would automatically be transferred to patients. Fourth, considering that the readmission rate in the present study was the highest in the first 30 days after surgery, while most alerts were initiated in months 3 and 6, the ideal PROM time intervals and the combination with other measures (eg, sensory technology feedback) should be further investigated.

### Strengths and Limitations

This study has some strengths. To our knowledge, it is the first randomized clinical trial evaluating the effect of PROM-based remote monitoring among patients receiving joint replacements. The randomized clinical trial design has several advantages, including randomization on the patient level, inclusion of multiple hospitals, and patient blinding, as well as the nonchangeability of group assignment via study nurses through the digital solution, and the large sample size. Moreover, the mixed-effects model accounts for several potential confounders. Because the intervention was not separated from routine care, the trial can give insights into the translation into routine practice.

This study also has some limitations. The main limitation is the difficulty of isolating the intervention effect. We cannot determine which part of the intervention impacted the changes in health outcomes. Moreover, we cannot determine whether the differences were meaningful to patients or might have been partially explained through a learning effect. On the one hand, it would have been helpful to include earlier PROMs without alerts among the control group to evaluate whether a treatment effect before the 12-month mark could be observed. On the other hand, the control and intervention groups would have been more similar by both experiencing the monitoring step of the intervention. As in routine care, there might have been heterogeneity in the operationalization of the study design across hospitals. This possibility was addressed as much as possible via the study protocol, workshops, and regular check-ins with study nurses from all hospitals and controlling for the hospital in the mixed-effect model. Additionally, results might differ depending on the hospital implementing the intervention (eg, those with lower case volumes).

## Conclusion

This secondary analysis of a randomized clinical trial found that the PROM monitoring and alert intervention, compared with the standard of care, led to small improvements in HRQOL and fatigue among patients with hip replacement and patients with knee replacement and in depression symptoms among patients with hip replacement. Whether the intervention is also cost-effective needs to be evaluated. Further research on the ideal time intervals, the timeframe, and effects of the different intervention steps, especially the potential caring effect of the monitoring and PROM-based telephone call follow-up conversations, is needed.

## Notes

Supplement 1.
Study Protocol and Statistical Analysis Plan

Supplement 2. **eFigure 1.** Participant Flow in the “PROMoting Quality” Trial With Detailed Information on the Reasons for Exclusion **eTable 1.** Adjustments to the Study Protocol **eTable 2.** Absolute Intervention Alert Thresholds for Each PROM-Score **eTable 3.** Descriptive Statistics of the Compliance Corrected Study Population **eTable 4.** Descriptive Statistics of the Admission, Discharge and 12-Month Follow-up PROM-Scores **eFigure 2.** Raw PROM-Score Distributions (Baseline, 12 Months) for the Control and Intervention Groups in the Intention to Treat Population for the EQ-5D-5L **eTable 5.** Readmission and Reoperation Descriptive Statistics in the Intervention Versus the Control Group Separated per Joint Replacement Type **eTable 6.** Information on Critical Values and Reactions to PROM-Based Critical Value Alerts From Study Assistants and Patients -Critical Values and Alert Reactions **eFigure 3.** Count of Hip Replacement Patients Whose PROM-Scores Initiated Alerts per Intervention Time/Times **eFigure 4.** Count of Knee Replacement Patients Whose PROM-Scores Initiated Alerts per Intervention Time/Times **eTable 7.** Therapy Changes in the Intervention Group of Patients Who Visited a Physician and Were Called Versus Not Called **eFigure 5.** Classification of Notes by Study Nurses of Patients Who Were Not Called Following an Alert (n= 560) **eTable 8.** Difference in the PROM-Score-Change From Baseline to 12 Months Post-Surgery, Between Intervention and Control Group (Compliance Corrected Study Population) **eTable 9.** Mixed Effects Models With PROM-Score Changes as Outcomes, Controlling for Gender, Age, Mobilization and Admission PROM Scores as Fixed Effects and Hospital as Random Intercept (Intention to Treat Study Population) **eTable 10.** Mixed Effects Models With PROM-Score Changes as Outcomes, Controlling for Gender, Age, Mobilization and Admission PROM Scores as Fixed Effects and Hospital as Random Intercept (Compliance Corrected Study Population) **eTable 11.** Mixed Effects Models With PROM-Score Change Z-Scores as Outcomes, Controlling for Gender, Age, Mobilization and Admission PROM Scores as Fixed Effects and Hospital as Random Intercept (Intention to Treat Study Population) **eTable 12.** Mixed Effects Models With PROM-Score Change Z-Scores as Outcomes, Controlling for Gender, Age, Mobilization and Admission PROM Scores as Fixed Effects and Hospital as Random Intercept (Compliance Corrected Study Population)

Supplement 3.
Data Sharing Statement
