# Supplementary material for: Electronic Patient-Reported Outcome Monitoring to Improve Quality of Life After Joint Replacement: Secondary Analysis of a Randomized Clinical Trial
Source: JAMA Netw Open. 2023 Sep 1;6(9):e2331301. doi: 10.1001/jamanetworkopen.2023.31301 (PMC10474554; doi:10.1001/jamanetworkopen.2023.31301)
Supplement: Supplement 1. — Study Protocol and Statistical Analysis Plan [file jamanetwopen-e2331301-s001.pdf]

Ethics committee protocol, data protection plan and analysis plan

Contents:

1) Ethics committee protocol – English translation (August, 2019)
2) Ethics committee protocol – German original (August, 2019)
3) Data protection plan – English translation (July, 2021)
4) Data protection plan – German original (July, 2021)
5) Analysis plan as mandated by the funding institution – English translation (September 2020)
6) Analysis plan as mandated by the funding institution – German original (September 2020)

Subscribe to DeepL Pro to translate larger documents.
Visit www.DeepL.com/pro for more information.

CHARITÉ

Campus Benjamin Franklin | Campus Buch | Campus Mitte | Campus Virchow-Klinikum

Ethics application for the project: PROMoting Quality

Dear Sir or Madam,

Enclosed I am sending you the ethics application for the above project for the meeting date 21.08.2019. The project is being carried out under the direction of Prof. Dr. med. Reinhard Busse - Technical University - Department of Management in Health Care with participation of the Charité and other study centers.

The cost center for the project is: 90100200 Please

do not hesitate to contact me if you have any

questions.

With kind regards

Claudia Hartmann
Project management in the Value-Based Healthcare department

Appendix:

1. Approval of the clinic director
2. Participant information
3. Declaration of consent
4. Statement of the Data Protection Officer of the TU Berlin
5. Formulated data protection concept
6. Data protection impact assessment
7. Support methodologist evaluation concept
8. Methodology threshold determination

Notes:

We would like to refer separately to the supporting statement of the official data protection officer of the TU Berlin (*Annex 1*), as well as the consent of the Center for Musculoskeletal Surgery of the Charité (*Annex 2*).
In *Appendix 3* (Formulated data protection concept), reference is made to additional appendices to the document, which are not attached to this application due to their large volume. They have been carefully checked by the data protection officer of the TU Berlin and have been submitted to all participating clinics for review. Of course, the attachments can be submitted later upon request.

For reasons of better readability, job and function designations are always used in the masculine form in this document. However, the designations in each case include persons of female, male and diverse genders equally.

Request for advice from the ethics committee on the implementation of a medical-scientific project that does not involve the clinical testing of a drug or medical device

1. title of the study	PROMoting Quality - Intersectoral use of Patient Reported Outcome Measures to increase patient-relevant outcome quality.
2. ethics committee application number	*(awarded by the EC)*
3. decisions of other ethics committees on the same matter	No
4. subject of the study and its objectives; state the hypotheses, separated into main and secondary hypotheses, and the clinical parameters (primary and secondary endpoints) against which the hypotheses are tested	The overall aim of the study is to enable early identification and thus prompt treatment of post-operative complications after implantation of an endoprosthesis of the hip or knee.<br>Knee joint (OPS codes 5-820 and 5-822) and/or quality-of-life-reducing abnormalities using Patient Reported Outcome Measures (PROMs)<br>1. **The primary outcome** is an increase in cost-effectiveness (optimization of the ratio of quality of results to resources expended).<br>2. **Secondary key endpoints**<br>PROM indicators according to ICHOM standard set:<br>- Improvement of functional status (assessed via HOOS-PS / KOOS-PS score)<br>- medium- to long-term quality of life (assessed via EQ-5D-5L score)<br>- Depression and exhaustion (assessed via PROMIS 29 - Depression & exhaustion)<br>- Pain in hip, knee or lower back (assessed via analog pain scale)<br><br>Clinical Outcome Indicators:<br>- postoperative readmission and re-operation rate
5. explanation of the significance of the study	PROMoting Quality is a randomized, controlled, multicenter intervention study. Patients with hip and knee TEP will be fully recruited within the study over a 12-month period. Recruited patients assigned to the control or intervention group will then each be followed up for a period of 12 months from the date of surgery/target procedure. For intervention and control groups, PROMs will be collected at hospital admissions as baseline, at hospital discharge, and at 12 months. In the intervention group, PROMs are additionally collected at 1, 3, 6 months.<br><br>The participating clinics cover the German states of Bavaria, Berlin, Brandenburg, Hamburg, Lower Saxony, Schleswig-Holstein and Thuringia. The clinics are among the leading endoprosthetics clinics in Germany and cover a wide range of specialty, teaching, and university hospitals.<br><br>PROM instruments are also to be used in Germany

	become an important component of quality-based remuneration. However, there is little experience in Germany with the introduction of this instrument, even across different hospitals and hospital owners.<br>Furthermore, pre- and postoperative patient quality of life surveys will be used to determine the value and benefits of treatments.<br>This type of quality measurement not only results in potential for improvement in the treatment of specific patient groups, but also the possibility of more individualized treatment.<br>At the same time, the study can provide valuable insights for the use of real word evidence.<br><br>Since the surveys are being conducted in a comparatively large study population of over (targeted) 10 thousand patients, significant results can be expected. These should provide information about the potential to transfer the project to standard care in the long term. The potential here is derived from an increase in cost-effectiveness, feasibility and value of the data for additional analyses with regard to the relevant determinants of outcome quality.
6. Which of the following provisions apply<br>a) Medical Devices Act according to § 23 b MPG - exception of clinical. Testing<br>b) Radiation Protection Ordinance § 23<br>c) X-ray Ordinance § 28 a<br>d) Gene Diagnostics Act<br>e) Data protection laws:<br>- Specific indication of the data protection law to be complied with by the responsible body (for Charité = Berlin Data Protection Act - BlnDSG).<br>- If applicable, additional state data protection laws or BDSG to be observed according to the group of participants.	e) The collection, storage and processing of personal data in this project is carried out in accordance with the Basic Data Protection Regulation (DGSVO) in conjunction with the applicable data protection regulations of the federal states and taking into account the specific provisions of the German Social Code, Book Ten (SGB X).
7. if applicable: designation and characterization of the test products	Not applicable.
8. significant results of the preclinical tests or reasons for not performing them.	-
9. essential content and results of previous studies/applications of the products to be tested in the study	-
10. description of the planned measures/examination methods and any deviations from the measures/examinations commonly used in medical practice (what is "routine", what is done differently in the study?)	The patient-centered survey instrument of the "Patient Reported Outcome Measures" (PROMs) is used to evaluate the course of treatment in the use of hip or knee joint prostheses also from the patient's perspective. Using an intuitive web solution, patients are continuously questioned about their state of health before and after their operation. To this end, patients regularly receive an e-mail asking them to complete a questionnaire.

	Provided that the patient continues to give his or her consent, conspicuous results can be sent to his or her aftercare physician - this enables him or her to intervene at an early stage if necessary and improve the success of the treatment in a targeted manner. On the other hand, the patient also receives valuable information that gives him transparency about the course of treatment and allows him to influence an improvement in discussions with his post-treatment physician.<br><br>Patients are supervised by at least one study assistant in all centers, who explains the study to them, conducts the first survey together with them if desired, and is available for queries at any time. In addition, she supports the patients, if necessary, in completing the follow-up surveys and contacts patients in the intervention group should they exceed critical thresholds.<br>(The assessment and definition of the critical thresholds was performed by physicians of the participating hospitals under the guidance of the TU scientists and on the basis of literature research; the derivation procedure can be found in *Appendix 8*).
11. evaluation and weighing of the foreseeable risks and disadvantages of study participation against the expected benefits for the study participants and persons who will become ill in the future (risk-benefit balance).	Risks and disadvantages are not expected as there is no invasive intervention through the study.<br>The time burden of interviews for patients is approx. 15 - 30 minutes per interview time. Interviews will only be conducted with patients who are in good health and who consent to participate in the study.<br>Participants in both the intervention and control groups will receive identical inpatient treatment.
a. Medical benefit to be tested for the study participants (individual benefit for the individual patient).	Patients benefit from a more individualized, timely approach and treatment. If they exceed defined thresholds (if, for example, a deterioration in general health or an increase in pain becomes apparent), the responsible study assistant contacts them to discuss the results and possible problem on the basis of an individual results statement and to discuss seeing the follow-up physician. With the patient's consent, the data will then also be forwarded to the post-treatment physician.
b. Medical benefit to be tested for persons with the disease in the future **(**group benefit).	In addition to the early identification of unfavorable courses at the individual level, PROMs allow the assessment of therapeutic success from the patient's perspective, which can be incorporated in the improvement of existing therapeutic approaches and the future selection of therapeutic modalities (e.g., surgical techniques).<br><br>The project is funded within the framework of the Innovation Fund. This explicitly provides for an examination of the "translation", i.e. the transfer of the project into standard care, if the added value for care has been proven.

c. **Risks** and burdens to study participants (list all in detail).	There are no disadvantages or risks to study participants for the following reasons:<br>- **Randomization and blinding**: This is a randomized, blinded study: Both study assistant and patient do not know at admission and discharge from the hospital whether they belong to the intervention or control group, which means that there is no influence on inpatient treatment. The software used automatically randomizes patients into hip or knee patients upon discharge from the hospital after stratification.<br>- **Pseudonymization**: The primary data is pseudonymized in the software according to the UUID standard (Universally Unique Identifier).<br>- **Data encryption**: Data is stored exclusively in encrypted form and can only be read by authorized users with a password (see *Appendix 1* for details).<br>- **Time required**: The time required for data collection is low and no medical understanding is required to answer the questions.<br>The data privacy impact assessment *(Annex 2)* sets out further potential risks and measures to avoid them.
12. measures to control risks	In each clinic, remuneration for the study assistance responsible for the participating patients is ensured throughout the entire data collection period. The specialists ("study assistance") receive on-site training by the technical provider of the survey tool. The study assistance will be prepared for the study design and its implementation in training sessions and with appropriate training material. The provider and also the consortium management of the project (TU Berlin) are available for any inquiries from the specialists.
13. termination criteria	Since the study intervention consists exclusively of the regular recording of PROMs as well as any resulting referral for treatment in the routine care system, negative effects for patients are not expected. Criteria for study discontinuation were therefore not established. The HRTBT Medical Solutions GmbH survey tool has additionally been in use at many different clinics for some time.
14. number, age and sex of the persons concerned	Based on current estimates, it is planned that a total of 9,833 patients of each sex aged 18 years and older will be enrolled in the study during the 12-month recruitment period beginning October 01, 2019.
15. biometric planning with indication of statistical methodology, including justification of the number of cases.<br>Specification of the statistician	**Survey timing: For** all patients meeting the inclusion criteria, a baseline survey will be conducted at inpatient admission and after written informed consent for study participation using the heartbeat software, thus taking place before the surgical intervention at the partner hospital. Both intervention and control groups conduct a second survey on the day before discharge from the hospital.

	In the intervention group only, three additional PRO surveys will be performed using software, at months 1, 3, and 6 after surgery.<br>Both groups last respond to the questionnaire 12 months after their procedure and discharge.<br><br>**Case number calculation:** Based on internal case numbers from 2017, a total of approximately 16,526 hip or knee joint implantations as defined by OPS codes 5,820 and 5,822, respectively, can be expected at the partner hospitals of PROMoting Quality within 12 months. Patients with operations after fractures, with revisions and under the age of 18 years are already excluded.<br><br>Experience suggests that about 15% of patients with hip or knee joint implants refuse to participate in the study due to personal reasons. In addition, it is assumed that despite intensive efforts, no PROMs can be collected from 30% of the study participants during the final survey. Based on these estimates, it is anticipated that 9,833 patients will be enrolled and complete the study within 12 months.<br>On the one hand, this figure is still likely to be reduced by a certain proportion of patients without a direct or indirect Internet access option; on the other hand, it is to be expected that in the meantime more hip and knee joint implantations will tend to be performed in the partner clinics than in 2017.<br><br>Based on 9,833 study participants, the number of insured persons or<br>population shares of the health insurers participating in the study, it can be assumed that routine data and thus health-related expenditures on a total of an estimated 2,598 study participants can be taken into account within the framework of PROMoting Quality.<br><br>Thus, statistically robust results can be expected for both the primary outcome and the secondary key endpoints (see also the cover letter from the responsible methodologist in *Appendix 7*).
16.<br>a. Presentation and explanation of the **inclusion and exclusion criteria,** if applicable.	The target population includes all adult patients undergoing elective hip or knee replacement surgery who can be assigned to ASA classes 1 (normal, healthy patient), 2 (patient with mild general disease), and 3 (patient with severe general disease).<br><br>Exclusion criteria include emergencies (e.g., near-hip femur fracture); patients in ASA- classes 4 (patient with life-threatening illness), 5 (morbid patient unlikely to survive without surgery), and 6 (deceased patient with established brain death, organ donor); patients younger than 18 years.<br>In addition, patients are discouraged from participating in

	excluded, who are foreseeably neither directly nor indirectly reachable via email for the duration of the study and who do not have access to the internet. Patients who do not wish to participate in the study will not be included.
b. Study information (who gives this verbally and in writing and indication of how much time is left between information and consent (written information as an attachment).	The participant information can be found in *Appendix 5.* Verbal information about the study is provided in a discussion with the study assistant of the respective hospital on site according to a standard protocol. Patients can decide on their own to participate in the study before their elective surgery and will then be informed immediately by the study assistant.
c. Declaration of consent (written form as attachment)	See *Exhibit 6.*
d. If applicable, information and consent of the legal representative (if applicable, also description of the procedure for the establishment of judicial care)	Not applicable, as participants who are under legal guardianship are not included in the study.
17. measures to attract study participants (notice board ?, newspaper advertisements? Etc.)	Study participants will be made aware of the study by the study assistant only upon hospital admission for their elective knee or hip procedure.
18. if applicable: reason for inclusion and demonstration of therapeutic benefit for individuals who are minors and/or unable to consent.	Minor patients will not be included in the study.
19. relationship between study participant and study physician (is the study physician also the treating physician?)	Since the study is conducted in the context of standard care, no study physicians are employed. Both study participants and non-study participants will be cared for by the treating physicians of the partner hospitals.
20. statement on the involvement of persons possibly dependent on the sponsor	Not applicable.
21. measures that allow a determination of whether a study participant is participating in more than one study at the same time or before the end of a period specified in the previous study.<br>Is it possible to participate in more than one study?	In principle, participation in other studies is possible and does not affect participation in this study.
22. if applicable: remuneration or reimbursement of study participants (amount, for what should be paid?)	Study participants will not receive compensation and will participate in the study as part of standard care.
23. if applicable: plan for the further treatment and medical care of the persons concerned after the end of the study.	Not applicable.

24. if applicable: insurance of the study participants (confirmation of insurance and insurance conditions, insurer, scope of insurance, duration of insurance).	Not applicable.
25. Documentation procedures:<br>- Reference to CRF sheets, if applicable.<br>- Sample handling<br>- Retention / archiving (incl. deadlines)<br>- Access to the data and samples	The study assistant can only access the data in the Heartbeat Portal with a personalized login. There is only a read-only right to patient responses.
26. if applicable: description of how the health status of healthy affected persons is to be documented.	Not applicable (patients undergoing inpatient care for an elective procedure as part of routine care are recruited).
27 If applicable: methods to identify, document and report adverse events (when, by whom and how ??).	Early identification of adverse trajectories is part of the intervention.
28. Procedure to protect the confidentiality of stored data, documents and, if applicable, samples, presentation of pseudonymization or anonymization of data and samples of study participants **(initials and date of birth as coding scheme are not allowed!).**<br>- Description of the separation of medical records, study documentation and allocation of personal data.<br>- Identification of access rights, including access to participant identification lists, during and after study implementation<br>- Detailed specification of the procedures for transmission, encryption, blocking and deletion (including specification of the network structure used, if any, and servers used).<br>-if necessary, access to identifying data for legally authorized auditors (third parties) for the purpose of inspecting the files required for this purpose.	**Data from patient survey**: Encrypted on the server of the respective clinic and can only be decrypted (made readable) with user access.<br><br>**Access rights**: Readable by individual logins for involved parties from: Nursing, Physician, Study Management, and Administration.<br><br>Detailed descriptions of the procedures for transferring, encrypting, blocking and deleting data can be found in the data protection concept (see *Appendix 3*). These are therefore only outlined below:<br>- **Procedure to transfer:**<br>In order to carry out the data transfer, the participating clinics, the cooperating health insurance companies, as well as the Department of Health Care Management at the TU Berlin are provided with Each user is provided with personalized access to the SFTP server of the evaluating aQua institute. Each user receives a separate drive area on this server on which transport-encrypted (AES-256) data can be stored or retrieved.<br>- **Method to encryption:**<br>The primary data are pseudonymized in the software according to the UUID standard (Universally Unique Identifier). The identification codes of the hospital case record are replaced by the clinics with the pseudonym (automatically generated in the software). Re-identification is not possible at any time.<br>**- Procedure for blocking**: If a patient revokes his or her consent, the study assistant sets a corresponding blocking note in the master data of the respective patient in the system.<br>**- Procedure for deletion**: In the clinics, the collected primary data are deleted after the end of the evaluation period and the participation and consent forms are destroyed. If a study participant revokes his/her consent to data processing before the end of the data collection period, the corresponding data will be deleted by the study assistance and no further data will be collected. If the participant revokes his or her consent during the data collection period, the

	Deletion of data also to be carried out manually in the system. This requires fewer changes by the study assistant.<br>To allow for verifiability of study results after project completion, data will be retained for a period of 10 years after project completion and then deleted.
29. Declaration of compliance with data protection<br>- Assurance that all data collected and stored about the study participant will be treated confidentially (data secrecy and medical confidentiality).<br>- Assurance that the identifying data will only be disclosed to the study director or by him/her authorized employees are accessible.<br>- Indication of the measures taken to ensure confidentiality<br>- Measures for the data-protection-compliant transfer of data that do not allow third parties to establish a personal reference.<br>- Indication of the information, objection and deletion options,<br>- Measures to ensure the rights of participants.	- All data of the study participant will be collected according to the principle of data economy. It is contractually stipulated in the cooperation agreement that the cooperation clinic bears the organizational responsibility for ensuring that the employees involved in processing the data of the cooperation clinic (i.e. study assistance and clinic controlling) are bound to data secrecy and are instructed in the corresponding protection provisions.<br>- The cooperation clinic assures the implementation of and compliance with the agreed general, technical and organizational measures in its area of responsibility. In particular, the cooperation clinic will design its internal organization in such a way that it meets the special requirements of data protection. It will take technical and organizational measures to adequately secure the data of the cooperation clinic against misuse and loss, which comply with the requirements of the BDSG.<br>- The patients' data are processed exclusively pseudonymously. Patients consent to the disclosure of their identification characteristics (such as e.g. the health insurance number to the health insurance company so that it can approve the respective declaration of consent) explicitly.<br>- The procedure in the event of a revocation by the study participant is regulated in detail. A sample objection form is also enclosed with the study information so that the patient - should he decide to withdraw - has a form directly at hand. In addition, the study assistant who enrolled the patient in the study is available to answer any questions.<br><br>For further details, see data protection concept (Annex 3)
30. names and addresses of the institutions involved in the study as study centers or study laboratories, as well as the study directors and the study physicians<br>- Detailed information on external service providers involved with details of data access options	**Study management (consortium management of the project) Technical University - Department of Healthcare Management**<br>Prof. Dr. med. Reinhard Busse<br>*TU Berlin, Straße des 17. Juni 135, Secretariat H80, 10623 Berlin*<br><br>**Study Centers**<br>**Charité - University Medicine Berlin**<br>Prof. Dr. med. Carsten Perka<br>*Charité, CMSC, Campus Virchow Augustenburger Platz 1, 13353 Berlin*<br>**Helios Endo Clinic Hamburg**<br>Prof. Dr. med. Thorsten Gehrke<br>*Helios ENDO-Klinik Hamburg GmbH, Holstenstraße 2, 22767 Hamburg*

	**Helios Ostseeklinik Damp**<br>Wolfgang Klauser, MD<br>*VAMED Ostseeklinik Damp GmbH, Seute-Deern-Ring 20, 24351 Ostseebad Damp* **Orthopedic Clinic of the MHH in the Annastift** Prof. Dr. med. Henning Windhagen<br>*DIAKOVERE Annastift, Anna-von-Borries-Straße 1-7, 30625 Hanover*<br>**RoMed Clinic Prien a. Chiemsee**<br>Jens Deerberg-Wittram, M.D.<br>*RoMed Kliniken, P.O. Box 100764, 83007 Rosenheim, Germany*<br>**Schön Klinik Hamburg Eilbek**<br>Prof. Dr. med. Frank Lampe<br>*Schön Klinik Hamburg Eilbek, Dehnhaide 120, 22081 Hamburg*<br>**Schön Klinik Neustadt**<br>Prof. Dr. med. Karl Christian Westphal<br>*Schön Klinik Neustadt, Am Kiebitzberg 10, 23730 Neustadt i. H.*<br>**Sana Clinics Sommerfeld**<br>Prof. Dr. med. Andreas Halder<br>*Sana Kliniken Sommerfeld, Waldhausstraße 44, 16766 Kremmen*<br>**Forest Clinics Eisenberg**<br>Prof. Dr. med. Georg Matziolis<br>*Waldkliniken Eisenberg GmbH, Klosterlausnitzer Straße 81, 07607 Eisenberg*<br><br>**External service providers involved**<br>**aQua - Institute**<br>Dr. Thomas Grobe<br>*aQua - Institute for Applied Quality Promotion and Research in Health Care, Maschmühlenweg 8- 10, 37073 Göttingen, Germany*<br><br>**Heartbeat Medical**<br>Yannik Schreckenberger<br>*HRTBT Medical Solutions GmbH, Greifswalder Str. 212, 10405 Berlin*<br><br>Heartbeat Medical is a technical service provider and does not have access to data.<br>The aQua Institute is responsible for the scientific evaluation of the project and evaluates primary data, hospital case data and health insurance company routine data, in each of which there are no longer any identification markers, i.e. person-identifying characteristics. No re-identification is possible.<br><br>The pseudonymized data are transmitted by the aQua Institute to the Department of Healthcare Management at the Technical University of Berlin, which also acts as a research institution.<br>Both research institutes carry out the project-related evaluations on a division of labor basis.
31. information on the suitability of the investigational site, in particular on the adequacy of the resources and facilities available there, as well as on the personnel available to conduct the clinical trial and on experience in conducting similar trials	The study is not a clinical trial and therefore a "trial site" with the described function does not exist.<br>The aQua Institute is responsible for the evaluation of outcomes of the study (www.aqua-institut.de). The aQua-Institut is an independent service provider in the scientific field, which currently employs more than 60 staff and is responsible for the evaluation and/or implementation of a variety of

	research projects, including more than ten projects funded under the Innovation Fund.
32. agreement on the access of the investigator/principal investigator/principal investigator to the clinical trial, to the data and to the principles on publication.<br>- Publications in a form that does not allow any inference about the person.	The type of data transfer and the access of the evaluating institute to the study data is described in detail in the data protection concept (cf. *Annex 3*).<br><br>Publications are intended exclusively on aggregated results, which do not allow any conclusions to be drawn about individual persons or facilities (hospitals).
33. information on the funding of the study: funding source (name and location) and amount of funding in €.	The study is funded by the Innovation Fund (Joint Federal Committee/Innovation Committee, Berlin) in the thematic area of<br>"Models of Care with Overarching and Measurable Outcome and Process Accountability" funded with a grant amount of €3,576,221.40 during the grant period from April 1, 2019 to March 31, 2023.

Name and signature of the applicant(s)

I hereby certify that the information provided in this application is correct. I believe that it is possible to conduct the above study in accordance with the Protocol, national legislation.

I am aware that according to §19 of the Berlin Data Protection Act (BlnDSG), I am obliged to create a file and procedure description for automated processing of personal and personal-related data and to make this available to the official data protection officer of the Charité according to §19a. I am informed that if the procedure involves the processing of data that is subject to professional secrecy (e.g., medical confidentiality), I must arrange for a prior check by the Charité's official data protection officer in accordance with §5 BlnDSG before using this procedure and that I may only use the procedure if the result of the check is positive.

Name: Hartmann

First name: Claudia

Address: Charitéplatz 1; 10117 Berlin

Position: Project Management in the Value-Based Healthcare

Department Date: 08/07/2019

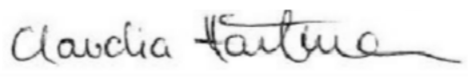

Signature: Claudia Hartmann

Campus Benjamin Franklin | Campus Buch | Campus Mitte | Campus Virchow-Klinikum

Ethikantrag zum Projekt: PROMoting Quality

Sehr geehrte Damen und Herren,

anbei übersende ich Ihnen den Ethikantrag zum o.g. Vorhaben für den Sitzungstermin 21.08.2019. Das Projekt wird unter der Leitung von Prof. Dr. med. Reinhard Busse - Technische Universität – Fachgebiet Management im Gesundheitswesen mit beteiligung der Charité und anderen Studienzentren durchgeführt.

Die Kostenstelle für das Projekt lautet: 90100200

Für Rückfragen stehe ich Ihnen gern zur Verfügung.

Mit freundlichen Grüßen

Claudia Hartmann
Projektleitung in der Abteilung Value-Based Healthcare

Anhang:

1. Zustimmung des Klinikdirektors
2. Teilnehmerinformation
3. Einwilligungserklärung
4. Stellungnahme der Datenschutzbeauftragten der TU Berlin
5. Ausformuliertes Datenschutzkonzept
6. Datenschutz-Folgenabschätzung
7. Unterstützung Methodiker Evaluationskonzept
8. Methodik Schwellenwertbestimmung

Anmerkungen:

Wir möchten gesondert auf die unterstützende Stellungnahme der behördlichen Datenschutz-beauftragten der TU Berlin (*Anlage 1*), sowie die Zustimmung des Centrums für Muskuloskeletale Chirurgie der Charité (*Anlage 2*) hinweisen.
In *Anlage 3* (Ausformuliertes Datenschutzkonzept) wird auf zusätzliche Anlagen des Dokuments verwiesen, die aufgrund des großen Umfangs diesem Antrag nicht beiliegen. Sie wurden sorgfältig von der Datenschutzbeauftragten der TU Berlin geprüft und wurden allen teilnehmenden Kliniken zur Prüfung vorgelegt. Selbstverständlich können die Anlagen auf Nachfrage nachgereicht werden.

Aus Gründen der besseren Lesbarkeit werden in diesem Dokument Berufs- und Funktionsbezeichnungen stets in der maskulinen Form verwendet. Die Bezeichnungen umfassen jedoch jeweils Personen weiblichen, männlichen und diversen Geschlechts gleichermaßen.

Antrag auf Beratung durch die Ethikkommission zur Durchführung eines medizinisch-wissenschaftlichen Vorhabens, welches weder die klinische Prüfung eines Arzneimittels oder Medizinproduktes beinhaltet

1. Titel der Studie	PROMoting Quality - Intersektorale Nutzung von Patient Reported Outcome Measures zur Steigerung der patientenrelevanten Ergebnisqualität
2. Ethikkommissions -Antragsnummer	*(wird von der EK vergeben)*
3. Entscheidungen anderer Ethikkommissionen in derselben Sache	Nein
4. Gegenstand der Studie und ihre Ziele; Angabe der Hypothesen, getrennt in Haupt- und Sekundärhypothesen sowie der klinischen Parameter (primäre und sekundäre Endpunkte), anhand derer die Hypothesen geprüft werden	Übergeordnetes Ziel der Studie ist die Ermöglichung der frühen Identifizierung und somit zeitnahen Behandlung post-operativer Komplikationen nach der Implantation einer Endoprothese am Hüft- bzw. Kniegelenk (OPS Codes 5-820 und 5-822) und/oder die Lebensqualität mindernder Fehlentwicklungen unter zur Hilfenahme von Patient Reported Outcome Measures (PROMs)<br>1. **Primärer Outcome** ist die Steigerung der Kosteneffektivität (Optimierung des Verhältnisses von Ergebnisqualität zu aufgewendeten Ressourcen)<br>2. **Sekundäre Schlüsselendpunkte**<br>PROM-Indikatoren gemäß ICHOM Standard Set:<br>- Verbesserung des funktionellen Status (bewertet via HOOS-PS / KOOS-PS Score)<br>- mittel- bis langfristige Lebensqualität (bewertet via EQ-5D-5L Score)<br>- Depression und Erschöpfung (bewertet via PROMIS 29 - Depression & Erschöpfung)<br>- Schmerzen in Hüfte, Knie oder unterem Rücken (bewertet via analoge Schmerzskala)<br><br>Klinische Ergebnisindikatoren:<br>- postoperative Wiederaufnahme- und Re-operationsrate
5. Erläuterung der Bedeutung der Studie	Bei PROMoting Quality handelt es sich um eine randomisierte, kontrollierte, multizentrische Interventionsstudie. Patienten mit Hüft- und Knie-TEP sollen im Rahmen der Studie innerhalb eines 12-monatigen Zeitraums vollständig rekrutiert werden. Die rekrutierten Patienten mit einer Zuordnung zur Kontroll- oder Interventionsgruppe werden dann jeweils über einen Zeitraum von 12 Monaten ab dem Operationstag/Zieleingriff nachbeobachtet. Bei Interventions- und Kontrollgruppe werden die PROMs bei Aufnahmen im Krankenhaus als Baseline, bei Entlassung aus dem Krankenhaus und nach 12 Monaten erhoben. Bei der Interventionsgruppe werden die die PROMs zusätzlich nach 1, 3, 6 Monaten erhoben.<br><br>Die teilnehmenden Kliniken decken die Bundesländer Bayern, Berlin, Brandenburg, Hamburg, Niedersachsen, Schleswig-Holstein und Thüringen ab. Die Kliniken gehören zu den führenden Endoprothetik Kliniken in Deutschland und decken ein weites Spektrum von Spezial-, Lehr-, und Universitätskrankenhäusern ab.<br><br>PROM-Instrumente sollen auch in Deutschland

	wichtiger Bestandteil der Qualitätsbasierten Vergütung werden. Doch gibt es in Deutschland wenig Erfahrung mit der Einführung dieses Instrumentes, auch über verschiedene Kliniken und Klinikträger hinaus.<br>Des Weiteren soll durch prä- und postoperative Patientenbefragungen über die Lebensqualität der Wert und der Nutzen von Behandlungen ermittelt werden. Durch diese Art der Qualitätsmessung ergeben sich nicht nur Verbesserungspotentiale in der Behandlung von bestimmten Patientengruppen, sondern auch die Möglichkeit einer individuelleren Behandlung. Gleichzeitig kann die Studie wertvolle Erkenntnisse für die Nutzung von Real Word Evidence liefern.<br><br>Da die Befragungen bei einer vergleichsweise großen Studienpopulation von über (anvisiert) 10 Tausend Patienten erfolgt, ist von signifikanten Ergebnissen auszugehen. Diese sollen Aufschluss über das Potenzial geben, das Projekt langfristig in die Regelversorgung zu übertragen. Das Potential leitet sich hier aus Steigerung der Kosteneffektivität, Umsetzbarkeit und Wert der Daten für zusätzliche Analysen in Bezug auf die relevanten Determinanten von Ergebnisqualität ab.
6. Welche der folgenden Bestimmungen finden Anwendung<br>a) Medizinproduktegesetz gemäß § 23 b MPG - Ausnahme der klin. Prüfung<br>b) Strahlenschutzverordnung § 23<br>c) Röntgenverordnung § 28 a<br>d) Gendiagnostikgesetz<br>e) Datenschutzgesetze:<br>- Konkrete Angabe des durch die verantwortliche Stelle zu erfüllenden Datenschutzgesetzes (für die Charité = Berliner Datenschutzgesetz - BlnDSG).<br>- Ggf. entsprechend des Teilnehmerkreises zusätzlich zu beachtende Landesdaten-schutzgesetze oder BDSG.	e) Die Erhebung, Speicherung und Verarbeitung der personenbezogenen Daten in diesem Projekt erfolgt gemäß der Datenschutzgrundverordnung (DGSVO) in Verbindung mit den jeweils geltenden Datenschutzbestimmungen der Länder und unter Berücksichtigung der spezifischen Bestimmungen des Sozialgesetzbuches, Zehntes Buch (SGB X)
7. Ggf.: Bezeichnung und Charakterisierung der Prüfprodukte	Entfällt.
8. Wesentliche Ergebnisse der vorklinischen Tests oder Gründe für die Nichtdurchführung derselben	-
9. Wesentlicher Inhalt und Ergebnisse der vorangegangenen Studien/Anwendungen der in der Studie zu prüfenden Produkte	-
10. Beschreibung der vorgesehenen Maßnahmen/Untersuchungsmethoden und eventuelle Abweichungen von den in der med. Praxis üblichen Maßnahmen/Untersuchungen (was ist „Routine“, was wird davon abweichend in der Studie gemacht?)	Es wird das patientenzentrierte Befragungsinstrument der „Patient Reported Outcome Measures“ (PROMs) eingesetzt, um den Behandlungsverlauf beim Einsatz von Hüft- oder Kniegelenkprothesen auch aus der Patientenperspektive zu bewerten. Über eine intuitiv nutzbare Web-Lösung werden die Betroffenen vor und nach ihrer Operation kontinuierlich zu ihrem Gesundheitszustand befragt. Dazu erhalten die Patienten regelmäßig eine E-Mail, in der sie gebeten werden, einen Fragebogen auszufüllen.

	Sofern der Patient weiterhin zustimmt, können auffällige Ergebnisse an seinen nachbehandelnden Arzt gesendet werden – dadurch kann dieser bei Bedarf frühzeitig eingreifen und den Behandlungserfolg gezielt verbessern. Zum anderen erhält auch der Patient wertvolle Informationen, mit denen er Transparenz über den Behandlungsverlauf erhält und im Gespräch mit seinem nachbehandelnden Arzt auf eine Verbesserung einwirken kann.<br><br>Die Patienten werden in allen Zentren von mindestens einer Studienassistenz betreut, die ihnen die Studie erklärt, die erste Befragung auf Wunsch gemeinsam mit ihnen durchführt und jederzeit für Rückfragen zur Verfügung steht. Zusätzlich unterstützt sie die Patienten, wenn notwendig, beim Ausfüllen der Nachbefragungen und kontaktiert Patienten der Interventionsgruppe, sollten diese kritische Schwellenwerte überschreiten.<br>(Die Einschätzung und Definition der kritischen Schwellenwerte wurde von Ärzten der teilnehmenden Kliniken unter Führung der TU Wissenschaftler und auf Basis von Literaturrecherchen vorgenommen; das Vorgehen der Herleitung ist *Anlage 8* zu entnehmen.)
11. Bewertung und Abwägung der vorhersehbaren Risiken und Nachteile der Studienteilnahme gegenüber dem erwarteten Nutzen für die Studienteilnehmer und zukünftig erkrankte Personen (Nutzen-Risiko-Abwägung)	Risiken und Nachteile sind nicht zu erwarten, da durch die Studie keine invasive Intervention stattfindet.<br>Die zeitliche Belastung durch Interviews beträgt für Patienten je Befragungszeitpunkt ca. 15 - 30 Minuten. Interviews werden nur bei Patienten durchgeführt, die hierzu gesundheitlich in der Lage sind und in die Studienteilnahme einwilligen.<br>Teilnehmer der Interventions- als auch Kontrollgruppe werden stationär identisch behandelt.
a. zu prüfender medizinischer Nutzen für die Studienteilnehmer (individueller Nutzen für den einzelnen Patienten)	Patienten profitieren von einer individuelleren, zeitnahen Ansprache und Behandlung. Sollten sie festgelegte Grenzwerte überschreiten (wenn z.B. eine Verschlechterung des allgemeinen Gesundheitszustands oder die Zunahme von Schmerzen ersichtlich wird), tritt die zuständige Studienassistenz mit ihnen in Kontakt, um die Ergebnisse und mögliche Problem auf Basis eines individuellen Ergebnisauszugs zu besprechen und das Aufsuchen des nachbehandelnden Arztes zu besprechen. Mit Zustimmung des Patienten werden die Daten dann auch an den nachbehandelnden Arzt weitergeleitet.
b. zu prüfender medizinischer Nutzen für zukünftig erkrankte Personen **(**Gruppennutzen)	Neben der frühzeitigen Identifikation ungünstiger Verläufe auf individueller Ebene, ermöglichen PROMs die Beurteilung von Therapieerfolgen aus Patientenperspektive, welche bei der Verbesserung bestehender Therapieansätze und der zukünftigen Auswahl von Therapieformen (z.B. von Operationstechniken) einbezogen werden können.<br><br>Das Projekt wird im Rahmen des Innovationsfonds gefördert. Dieser sieht eine Prüfung der „Translation“, also der Übertragung des Projektes in die Regelversorgung, bei entsprechendem nachgewiesenem Mehrwert für die Versorgung explizit vor.

c. **Risiken** und Belastungen für die Studienteilnehmer (alle im Einzelnen auflisten)	Für die Studienteilnehmer entstehen keine Nachteile oder Risiken aus den folgenden Gründen:<br>- **Randomisierung und Verblindung**: Es handelt sich um eine randomisierte, verblindete Studie: Sowohl Studienassistenz als auch Patient wissen bei Aufnahme und Entlassung aus dem Krankenhaus nicht, ob sie der Interventions- oder Kontrollgruppe zugehören, wodurch es keinerlei Einfluss auf die stationäre Behandlung gibt. Die eingesetzte Software randomisiert die Patienten automatisch bei Entlassung aus der Klinik nach einer Stratifizierung in Hüft- oder Kniepatienten.<br>- **Pseudonymisierung**: Die Primärdaten werden in der Software nach dem UUID Standard (Universally Unique Identifier) pseudonymisiert.<br>- **Datenverschlüsselung**: Die Daten werden ausschließlich verschlüsselt gespeichert und können nur durch autorisierte Nutzer mit Passwort gelesen werden (Details siehe *Anhang 1*).<br>- **Zeitlicher Aufwand**: Der zeitliche Aufwand für die Datenerhebung ist gering und für die Beantwortung der Fragen wird kein medizinisches Verständnis vorausgesetzt.<br>In der Datenschutz-Folgenabschätzung *(Anhang 2)* sind weitere potenzielle Risiken und Maßnahmen zur Vermeidung derselben dargelegt.
12. Maßnahmen zur Risikobeherrschung	In jeder Klinik wird die Vergütung für die Studienassistenz, die für die teilnehmenden Patienten zuständig ist, über den gesamten Datenerhebungszeitraum sichergestellt. Die Fachkräfte („Studienassistenz") erhalten vor Ort eine Schulung durch den technischen Anbieter des Befragungstools. Die Studienassistenz wird in Schulungen und mit entsprechendem Schulungsmaterial auf das Studiendesign und dessen Umsetzung vorbereitet. Der Anbieter und auch die Konsortialführung des Projekts (TU Berlin) stehen für jegliche Nachfragen von Seiten der Fachkräfte zur Verfügung.
13. Abbruchkriterien	Da die Studienintervention ausschließlich in der regelmäßigen Erfassung von PROMs sowie einer ggf. daraus folgenden Vermittlung von Behandlungen im Routineversorgungssystem besteht, sind negative Auswirkungen für Patienten nicht zu erwarten. Kriterien für einen Studienabbruch wurden daher nicht festgelegt. Das Befragungstool der HRTBT Medical Solutions GmbH ist zusätzlich seit längerem bei vielen verschiedenen Kliniken im Einsatz.
14. Anzahl, Alter und Geschlecht der betroffenen Personen	Geplant ist nach derzeitigen Abschätzungen, dass im Rekrutierungszeitraum von 12 Monaten ab dem 01. Oktober 2019 insgesamt 9.833 Patienten jeden Geschlechts ab einem Alter von 18 Jahren in die Studie eingeschlossen werden.
15. Biometrische Planung mit Angabe der statistischen Methodik, einschließlich der Begründung der Fallzahl.<br>Angabe des/der Statistikers/Statistikerin	**Befragungszeitpunkte:** Bei allen Patienten, die die Einschlusskriterien erfüllen, erfolgt bei der stationären Aufnahme und nach einer schriftlichen Einwilligung in die Studienteilnahme mittels der heartbeat-Software eine Baseline-Erhebung, die damit also noch vor dem operativen Eingriff in der Partnerklinik erfolgt. Sowohl Interventions- als auch Kontrollgruppe führen eine zweite Befragung am Tag vor der Entlassung aus der Klinik durch.

	Nur in der Interventionsgruppe erfolgen softwaregestützt drei weitere PRO-Erhebungen, und zwar in den Monaten 1, 3 und 6 nach dem operativen Eingriff.<br>Beide Gruppen beantworten zuletzt den Fragebogen 12 Monate nach ihrem Eingriff und der Entlassung.<br><br>**Fallzahlkalkulation:** Basierend auf der Angabe von internen Fallzahlen aus dem Jahr 2017 ist bei den Partnerkliniken von PROMoting Quality innerhalb von 12 Monaten mit insgesamt etwa 16.526 Implantationen eines Hüft- oder Kniegelenkes im Sinne der OPS-Codes 5.820 bzw. 5.822 zu rechnen. Bereits ausgeschlossen sind hierbei Patienten mit Eingriffen nach Frakturen, mit Revisionen sowie im Alter von unter 18 Jahren.<br><br>Erfahrungswerte lassen vermuten, dass rund 15% der Patienten mit Implantationen eines Hüft- oder Kniegelenkes die Studienteilnahme auf Grund von persönlichen Gründen ablehnen. Zudem wird davon ausgegangen, dass ungeachtet intensiver Bemühungen, bei 30% der Studienteilnehmer keine PROMs im Rahmen der abschließenden Befragung erhoben werden können. Basierend auf diesen Abschätzungen wird damit gerechnet, dass innerhalb von 12 Monaten 9.833 Patienten in die Studie eingeschlossen werden können und diese auch beenden.<br>Diese Zahl dürfte sich einerseits noch um einen gewissen Anteil von Patienten ohne eine unmittelbare oder mittelbare Internetzugangsmöglichkeit reduzieren, andererseits ist damit zu rechnen, dass in den Partnerkliniken zwischenzeitlich tendenziell mehr Implantationen von Hüft- und Kniegelenken als 2017 vorgenommen werden.<br><br>Bei Zugrundelegung von 9.833 Studienteilnehmern kann aufgrund der Versichertenzahlen bzw. Bevölkerungsanteilen der studienbeteiligten Krankenkassen davon ausgegangen werden, dass im Rahmen von PROMoting Quality Routinedaten und damit gesundheitsbezogene Ausgaben zu insgesamt schätzungsweise 2.598 Studienteilnehmern berücksichtigt werden können.<br><br>Damit können sowohl für den primären Outcome als auch für die sekundären Schlüsselendpunkte statistisch belastbare Ergebnisse erwartet werden (siehe dazu auch das Begleitschreiben des zuständigen Methodikers in *Anlage* 7).
16.<br>a. Darlegung und ggf. Erläuterung der **Ein- und Ausschlusskriterien**	Zur Zielgruppe gehören alle erwachsenen Patienten, die sich einem elektiven Eingriff zum Ersatz des Hüft- oder Kniegelenks unterziehen und den ASA-Klassen 1 (normaler, gesunder Patient), 2 (Patient mit leichter Allgemeinerkrankung) und 3 (Patient mit schwerer Allgemeinerkrankung) zugeordnet werden können.<br><br>Zu den Ausschlusskriterien zählen Notfälle (z.B. hüftgelenksnahe Femurfraktur); Patienten der ASA-Klassen 4 (Patient mit lebensbedrohlicher Erkrankung), 5 (Moribunder Patient, der ohne Operation wahrscheinlich nicht überleben wird) und 6 (gestorbener Patient mit festgestelltem Hirntod, Organspender); Patienten jünger als 18 Jahre.<br>Zudem werden Patienten von einer Studienteilnahme

	ausgeschlossen, welche absehbar über die Studiendauer weder mittelbar noch unmittelbar über Email erreichbar sind und welche keinen Zugang zum Internet haben. Patienten, die nicht an der Studie teilnehmen möchten, werden nicht eingeschlossen.
b. Studieninformation (wer diese mündlich und schriftlich erteilt und Angabe, wie viel Zeit zwischen Aufklärung und Einwilligung verbleibt (schriftliche Information als Anlage)	Die Teilnehmerinformation ist *Anlage 5* zu entnehmen. Die mündliche Aufklärung über die Studie erfolgt in einem Gespräch mit der Studienassistenz der jeweiligen Klinik vor Ort nach einem Standardprotokoll. Die Patienten können sich vor ihrem elektiven Eingriff selbstständig für eine Studienteilnahme entscheiden und werden dann unverzüglich von der Studienassistenz aufgeklärt.
c. Einwilligungserklärung (schriftliche Form als Anlage)	Siehe *Anlage 6*.
d. Ggf. Information und Einwilligung des gesetzlichen Vertreters (ggf. auch Beschreibung des Verfahrens zur Einrichtung einer gerichtlichen Betreuung)	Trifft nicht zu, da Teilnehmer, welche unter gesetzlicher Betreuung stehen nicht in die Studie eingeschlossen werden.
17. Maßnahmen zur Gewinnung von Studienteilnehmern (Aushang ?, Zeitungsannoncen? Etc.)	Die Studienteilnehmer werden ausschließlich bei Krankenhausaufnahme für ihren elektiven Eingriff an Knie oder Hüfte von der Studienassistenz auf die Studie aufmerksam gemacht.
18. Ggf.: Grund für die Einbeziehung und Darlegung des therapeutischen Nutzens für Personen, die minderjährig und/oder nicht einwilligungsfähig sind.	Minderjährige Patienten werden nicht in die Studie eingeschlossen.
19. Beziehung zwischen Studienteilnehmer und Studienarzt/-ärztin (ist der Studienarzt zugleich der behandelnde Arzt?)	Da die Studie im Kontext der Regelversorgung durchgeführt wird, werden keine Studienärzte beschäftigt. Sowohl Studienteilnehmer als auch Nicht-Studienteilnehmer werden von den behandelnden Ärzten der Partnerkliniken versorgt.
20. Erklärung zur Einbeziehung möglicherweise vom Sponsor abhängiger Personen	Nicht zutreffend.
21. Maßnahmen, die eine Feststellung zulassen, ob ein Studienteilnehmer an mehreren Studien zugleich oder vor Ablauf einer in der vorangegangenen Studie festgelegten Frist teilnimmt.<br>Ist die Teilnahme an mehreren Studien möglich?	Prinzipiell ist die Teilnahme an anderen Studien möglich und hat keinen Einfluss auf die Teilnahme an dieser Studie.
22. Ggf.: Honorierung bzw. Kostenerstattung der Studienteilnehmer (Höhe, wofür soll gezahlt werden?)	Die Studienteilnehmer erhalten keine Vergütung und nehmen an der Studie im Rahmen der Regelversorgung teil.
23. Ggf.: Plan für die Weiterbehandlung und medizinische Betreuung der betroffenen Personen nach dem Ende der Studie	Nicht zutreffend.

24. Ggf.: Versicherung der Studienteilnehmer (Versicherungsbestätigung und Versicherungsbedingungen, Versicherer, Versicherungsumfang, Versicherungsdauer)	Nicht zutreffend.
25. Dokumentationsverfahren:<br>- Ggf. Verweis auf CRF-Bögen.<br>- Probenumgang<br>- Aufbewahrung / Archivierung (inkl. Fristen)<br>- Zugang zu den Daten und Proben	Der Zugang der Studienassistenz zu den Daten im Heartbeat Portal erfolgt nur mit personalisiertem Login. Es besteht nur ein Leserecht auf Patientenantworten.
26. Ggf.: Beschreibung, wie der Gesundheitszustand gesunder betroffener Personen dokumentiert werden soll	Nicht zutreffend (es werden Patienten rekrutiert, die sich im Rahmen der Routineversorgung für einen elektiven Eingriff in stationäre Betreuung begeben).
27. Ggf.: Methoden, unerwünschte Ereignisse festzustellen, zu dokumentieren und mitzuteilen (wann, von wem und wie ??)	Die frühzeitige Identifizierung von ungünstigen Verläufen ist Bestandteil der Intervention.
28. Vorgehen zum Schutz der Geheimhaltung der gespeicherten Daten, Dokumente und ggf. Proben, Darlegung der Pseudonymisierung oder Anonymisierung der Daten und Proben von Studienteilnehmern **(Initialen und Geburtsdatum als Codierungsschema sind nicht zulässig!)**<br>- Beschreibung der Trennung von Krankenakten, Studiendokumentation und Zuordnung der personenbezogenen Daten<br>- Nennung der Zugriffsrechte einschließlich des Zugangs zu Teilnehmeridentifikations-listen während und nach der Studiendurch-führung<br>- Detaillierte Angabe der Verfahren für die Übertragung, Verschlüsselung, Sperrung und Löschung (einschließlich Angabe der ggf. verwendeten Netzstruktur und verwendete Server).<br>-ggf. Zugang zu identifizierenden Daten für gesetzlich berechtigte Prüfer (Dritte) zur zweckgebundenen Einsichtnahme in die dafür erforderlichen Akten.	**Daten aus Patientenbefragung**: Liegen verschlüsselt auf dem Server der jeweiligen Klinik und können nur mit Nutzerzugang entschlüsselt (lesbar gemacht) werden.<br><br>**Zugriffrechte**: Lesbar durch individuelle Logins für beteiligte Parteien aus: Pflege, Arzt, Leitung der Studie und Administration.<br><br>Es finden sich detaillierte Beschreibungen zu den Verfahren der Übertragung, Verschlüsselung, Sperrung und Löschung der Daten im Datenschutzkonzept (siehe *Anlage 3*). Im Folgenden werden diese deshalb nur skizziert:<br>- **Verfahren zu Übertragung:**<br>Zur Durchführung der Datenübermittlung wird den beteiligten Kliniken, den kooperierenden Krankenkassen, sowie dem Fachgebiet Management im Gesundheitswesen an der TU Berlin jeweils ein personalisierter Zugang zum SFTP-Server des evaluierenden aQua-Instituts zu Verfügung gestellt. Jeder Nutzer erhält dazu auf diesem Server einen separaten Laufwerksbereich auf dem transportverschlüsselt (AES-256) Daten abgelegt, bzw. abgeholt werden können<br>- **Verfahren zu Verschlüsselung:**<br>Die Primärdaten werden in der Software nach dem UUID Standard (Universally Unique Identifier) pseudonymisiert. Die Identifikationskennzeichen des Krankenhaus-Falldatensatzes werden von den Kliniken durch das Pseudonym (automatisch generiert in der Software) ersetzt. Eine Re-Identifizierung ist zu keiner Zeit möglich.<br>**- Verfahren zu Sperrung**: Sollte ein Patient sein Einverständnis widerrufen, setzt die Studienassistenz einen entsprechenden Sperrvermerk in den Stammdaten des jeweiligen Patienten im System.<br>**- Verfahren zur Löschung**: In den Kliniken werden die erhobenen Primärdaten nach Ablauf des Auswertungszeitraumes gelöscht und die Teilnahme- und Einverständniserklärungen vernichtet. Sofern ein Studienteilnehmer vor Ablauf des Erhebungszeitraumes sein Einverständnis zur Verarbeitung der Daten widerruft, werden die entsprechenden Daten durch die Studienassistenz gelöscht und keine weiteren Daten erhoben. Widerruft der Teilnehmer während des Erhebungszeitraums sein Einverständnis, ist die

	Löschung der Daten ebenfalls manuell im System vorzunehmen. Dafür bedarf es weniger Änderungen durch die Studienassistenz.<br>Um nach Abschluss des Projektes eine Überprüfbarkeit der Studienergebnisse zu ermöglichen, werden die Daten für einen Zeitraum von 10 Jahren nach Projektabschluss aufbewahrt und anschließend gelöscht.
29. Erklärung zur Einhaltung des Datenschutzes<br>- Zusicherung, dass alle über den Studien-teilnehmer erhobenen und gespeicherten Daten vertraulich (Datengeheimnis und ärztliche Schweigepflicht) behandelt werden.<br>- Zusicherung, dass die identifizierenden Daten nur dem Studienleiter oder von ihm beauftragten Mitarbeitern zugänglich sind.<br>- Angabe der Maßnahmen zur Sicherstellung der Vertraulichkeit<br>- Maßnahmen zur datenschutzgerechten Übermittlung von Daten, die für Dritte keinen Personenbezug herstellen lassen.<br>- Angabe der Auskunfts- Widerspruchs- und Löschmöglichkeiten,<br>- Maßnahmen zur Sicherstellung der Rechte der Teilnehmer.	- Alle Daten des Studienteilnehmers werden nach dem Prinzip der Datensparsamkeit erhoben.<br>Im Kooperationsvertrag ist vertraglich festgelegt, dass die Kooperationsklinik die organisatorische Verantwortung dafür trägt, dass die mit der Verarbeitung der Daten der Kooperationsklinik befassten Mitarbeiter (also Studienassistenz und Klinikcontrolling) dem Datengeheimnis verpflichtet sind und in entsprechende Schutzbestimmungen eingewiesen werden.<br>- Die Kooperationsklinik sichert in ihrem Verantwortungsbereich die Umsetzung und Einhaltung der vereinbarten allgemeinen, technischen und organisatorischen Maßnahmen zu. Insbesondere wird die Kooperationsklinik ihre innerbetriebliche Organisation so gestalten, dass sie den besonderen Anforderungen des Datenschutzes gerecht wird. Sie wird technische und organisatorische Maßnahmen zur angemessenen Sicherung der Daten der Kooperationsklinik vor Missbrauch und Verlust treffen, die den Forderungen des BDSG entsprechen.<br>- Die Daten der Patienten werden ausschließlich pseudonymisiert verarbeitet. Die Patienten stimmen der Weitergabe ihrer Identifikationsmerkmale (wie z.B. der Krankenversichertennummer an die Krankenkasse, damit diese die jeweilige Einverständniserklärung zustimmen kann) explizit zu.<br>- Das Vorgehen im Falle eines Widerrufs des Studienteilnehmers ist ausführlich geregelt. Der Studieninformation wird zudem ein Muster-Widerspruchsformular beigelegt, damit der Patient - sollte er sich für einen Widerruf entscheiden – ein Formular direkt zur Hand hat. Zusätzlich steht ihm auch in dieser Frage die Studienassistenz, die ihn in die Studie eingeschlossen hat, für Nachfragen zur Verfügung.<br><br>Für weitere Details siehe Datenschutzkonzept (Anlage 3)
30. Namen und Anschriften der Einrichtungen, die als Studienzentrum oder Studienlabor in die Studie eingebunden sind, sowie der Studienleiter und die Studienärzte<br>- Detaillierte Angabe beteiligter externer Dienstleister mit Angabe der Datenzugriffs-möglichkeit	**Studienleitung (Konsortialführung des Projektes)**<br>**Technische Universität – Fachgebiet Management im Gesundheitswesen**<br>Prof. Dr. med. Reinhard Busse<br>*TU Berlin, Straße des 17. Juni 135, Sekretariat H80, 10623 Berlin*<br><br>**Studienzentren**<br>**Charité – Universitätsmedizin Berlin**<br>Prof. Dr. med. Carsten Perka<br>*Charité, CMSC, Campus Virchow Augustenburger Platz 1, 13353 Berlin*<br>**Helios Endo-Klinik Hamburg**<br>Prof. Dr. med. Thorsten Gehrke<br>*Helios ENDO-Klinik Hamburg GmbH, Holstenstraße 2, 22767 Hamburg*

	**Helios Ostseeklinik Damp**<br>Dr. med. Wolfgang Klauser<br>*VAMED Ostseeklinik Damp GmbH, Seute-Deern-Ring 20, 24351 Ostseebad Damp*<br>**Orthopädische Klinik der MHH im Annastift**<br>Prof. Dr. med. Henning Windhagen<br>*DIAKOVERE Annastift, Anna-von-Borries-Straße 1-7, 30625 Hannover*<br>**RoMed Klinik Prien a. Chiemsee**<br>Dr. med. Jens Deerberg-Wittram<br>*RoMed Kliniken, Postfach 100764, 83007 Rosenheim*<br>**Schön Klinik Hamburg Eilbek**<br>Prof. Dr. med. Frank Lampe<br>*Schön Klinik Hamburg Eilbek, Dehnhaide 120, 22081 Hamburg*<br>**Schön Klinik Neustadt**<br>Prof. Dr. med. Karl Christian Westphal<br>*Schön Klinik Neustadt, Am Kiebitzberg 10, 23730 Neustadt i. H.*<br>**Sana Kliniken Sommerfeld**<br>Prof. Dr. med. Andreas Halder<br>*Sana Kliniken Sommerfeld, Waldhausstraße 44, 16766 Kremmen*<br>**Waldkliniken Eisenberg**<br>Prof. Dr. med. Georg Matziolis<br>*Waldkliniken Eisenberg GmbH, Klosterlausnitzer Straße 81, 07607 Eisenberg*<br><br>**Beteiligte externe Dienstleister**<br>**aQua – Institut**<br>Dr. Thomas Grobe<br>*aQua – Institut für angewandte Qualitätsförderung und Forschung im Gesundheitswesen, Maschmühlenweg 8-10, 37073 Göttingen*<br><br>**Heartbeat Medical**<br>Yannik Schreckenberger<br>*HRTBT Medical Solutions GmbH, Greifswalder Str. 212, 10405 Berlin*<br><br>Heartbeat Medical ist technischer Dienstleister und hat keine Datenzugriffsmöglichkeit.<br>Das aQua-Institut ist für die wissenschaftliche Evaluation des Projekts verantwortlich und wertet Primärdaten, Krankenhaus-Falldaten und Krankenkassen-Routinedaten aus, in denen jeweils keine Identifikationskennzeichen, d.h. personenidentifizierende Merkmale mehr vorhanden sind. Es ist keine Re-Identifizierung möglich.<br><br>Die pseudonymisierten Daten werden vom aQua-Institut an das Fachgebiet Management im Gesundheitswesen an der TU Berlin, welches ebenfalls als forschende Einrichtung fungiert, übermittelt.<br>Beide forschenden Einrichtungen führen arbeitsteilig die projektbezogenen Auswertungen durch.
31. Angaben zur Eignung der Prüfstelle, insbesondere zur Angemessenheit der dort vorhandenen Mittel und Einrichtungen sowie des zur Durchführung der klinischen Prüfung zur Verfügung stehenden Personals und zu Erfahrungen in der Durchführung ähnlicher Studien	Bei der Studie handelt es sich nicht um eine klinische Studie, weshalb eine „Prüfstelle“ mit der beschriebenen Funktion nicht existiert.<br>Für die Evaluation von Outcomes der Studie ist das aQua-Institut zuständig (www.aqua-institut.de). Das aQua-Institut ist ein im wissenschaftlichen Bereich angesiedeltes, unabhängiges Dienstleistungsunternehmen, welches zurzeit mehr als 60 Mitarbeiter beschäftigt und mit der Evaluation und/oder Durchführung einer Vielzahl an

	Forschungsprojekten befasst ist, darunter mehr als zehn Projekte, die im Rahmen des Innovationsfonds gefördert werden.
32. Vereinbarung über den Zugang des Prüfers/Hauptprüfers/Leiter der klinischen Prüfung, zu den Daten und den Grundsätzen über die Publikation.<br>- Publikationen in einer Form, die keinen Rückschluss auf die Person zulassen.	Die Art der Datenübermittlung und der Zugang des evaluierenden Instituts zu den Studiendaten ist ausführlich im Datenschutzkonzept (vgl. *Anlage 3*) dargelegt.<br><br>Publikationen sind ausschließlich zu aggregierten Ergebnissen vorgesehen, die keinerlei Rückschlüsse auf einzelne Personen oder Einrichtungen (Krankenhäuser) erlauben.
33. Angaben zur Finanzierung der Studie: Finanzierungsquelle (Name und Sitz) und Höhe der Förderung in €.	Die Studie wird aus Mitteln des Innovationsfonds (Gemeinsamer Bundesausschuss/ Innovationsausschuss, Berlin) im Themenfeld „Versorgungsmodelle mit übergreifender und messbarer Ergebnis- und Prozessverantwortung“ im Förderzeitraum vom 1. April 2019 bis 31. März 2023 mit einem Förderbetrag von 3.576.221,40 € finanziert.

Name und Unterschrift des/der Antragsteller

Ich versichere hiermit, dass die in diesem Antrag gegebenen Informationen richtig sind. Ich bin der Auffassung, dass es möglich ist, die o.g. Studie in Übereinstimmung mit dem Protokoll, den nationalen Rechtsvorschriften durchzuführen.

Mir ist bekannt, dass ich gemäß §19 Berliner Datenschutzgesetz (BlnDSG) verpflichtet bin, für automatisierte Verarbeitungen personenbezogener und personenbeziehbarer Daten eine Datei- und Verfahrensbeschreibung zu erstellen und diese gemäß §19a dem behördlichen Datenschutzbeauftragten der Charité zur Verfügung stellen muss. Ich bin darüber informiert, dass wenn es sich um ein Verfahren handelt, mit dem Daten verarbeitet werden, die einem Berufsgeheimnis (z.B. ärztliche Schweigepflicht) unterliegen, ich gemäß §5 BlnDSG vor dem Einsatz dieses Verfahrens eine Vorabkontrolle durch den behördlichen Datenschutzbeauftragten der Charité veranlassen muss und ich das Verfahren erst bei positivem Prüfergebnis anwenden darf.

Name: Hartmann

Vorname: Claudia

Adresse: Charitéplatz 1; 10117 Berlin

Position: Projektleitung in der Abteilung Value-Based Healthcare

Datum: 07.08.2019

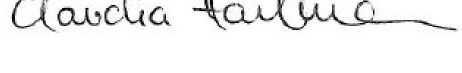

Unterschrift: Claudia Hartmann

Subscribe to DeepL Pro to edit this
Visit www.DeepL.com/pro for more information.

PROMoting Quality
Data protection concept

Status: 15.07.2021

Table of contents

1. Processing purpose 3
2. Principles of data protection law 3
3. Informed consent 3
4. Description of the data collected and justification of the data collection. 4
   Primary data4
   KH case data (secondary data) 5
   KK routine data (secondary data) 6
   Contact details of the post-treatment physician in private practice 6
5. Description of the data flow 7
   Participating institutions 7
   Data collection in the study centers 7
   Data transmission 9
   Pseudonymization procedure and data linking 10
   Storage, evaluation period and deletion periods 10
   Technical procedure for data transmission (transport encryption) 10
6. Description of the TOM of the researching institutions 12
   aQua institute 12
   Department of Healthcare Management at the TU Berlin 12
7. Definitions of terms 13
8. References 13

1. Processing purpose

The project "PROMoting Quality - Intersectoral Use of Patient Reported Outcome Measures to Improve Patient-Relevant Outcome Quality" (abbreviated PROMoting Quality), funded by the Innovation Fund of the G-BA, is a randomized, controlled, multicenter intervention study.

The intervention consists of regular monitoring of patients[1] by web-based questionnaires for up to one year after elective hip or knee replacement using Patient Reported Outcome Measures (PROMs).

The aim of the intervention is to positively influence patient-relevant outcomes (e.g., clinical outcome indicators, functionality and quality of life) of the patients, also by reflecting the results back to patients and post-treatment physicians. The aim of the study is to test whether measurably better outcomes and possibly lower treatment costs can be observed in the intervention group compared to the control group.

To carry out the study, primary data and secondary data will be collected, which will be linked in a personal but pseudonymized manner.
A total of three data packages are collected from different sources:

- Primary data
- KH case data
- KK route data

The primary data are data collected specifically for the purpose of the study. The other data packages are data routinely collected in the participating hospitals (N=9) (hospital case data), as well as routine data from the health insurance companies (health insurance company routine data) of those study participants who are insured with one of the cooperating health insurance companies (BARMER and health insurance companies belonging to the BKK umbrella organization).

On the one hand, data processing serves the implementation of the intervention (regular monitoring with query of PROMs). On the other hand, data processing is necessary to analyze the effects of the intervention and the influence of relevant treatment factors on the outcomes and thus to achieve the project goal.

2. Data protection principles

The collection, storage and processing of personal data in this project is carried out in accordance with the applicable data protection regulations of the federal states in conjunction with the German Data Protection Regulation (DSGVO) and taking into account the specific provisions of the German Social Code, Book Ten (SGB X).

3. Informed consent

The scientific use of the collected data is based on informed consent from the study participants. The target population is patients undergoing elective, i.e. planned, hip or knee replacement surgery. Each potential study participant meeting the inclusion criteria will be offered participation in the study prior to surgery. In this context, information is provided in an understandable and easily accessible form in clear and simple language about the type of data collected, data transmission, processing, storage and deletion, and the possibility of revocation.

[1] For reasons of better readability, job and function designations are always used in the masculine form in this document. However, the designations in each case include persons of female, male and diverse genders equally.

Based on this information, potential participants can make an informed decision for or against participation. This takes into account the principle of data processing that is comprehensible to the data subject (Article 5 DGSVO or Section 67b SGB X).

Informed consent is requested separately for the three data packages and confirmed in writing:

- Participation in the study in conjunction with the collection of personal data and PROMs (primary data) ***Prerequisite for inclusion in the study.***
- Collection, processing and personal linking of the medical treatment case data in the format according to § 21 KHEntgG (hospital case data) ***optional***
- Collection, processing and personal linking of routine health insurance data (KK-Routinedaten) for study participants who are insured with one of the cooperating health insurance companies (according to § 67b SGB X) ***optional***

Participants who agree to primary data collection but do not wish to have KH case data and/or KK routine data collected, linked, and processed will also be included in the study if they have agreed to participate. That is, in this case, only primary data would be collected.

Informed consent from study participants regarding study participation and the processing of KH case data and KK routine data will be kept at the respective hospital.

4. Description of the data collected and justification of data collection

The selection of the data to be collected is made within the framework of the study planning under the principle of data minimization (Article 5 GDPR).

Primary data

The collection of primary data is based on the internationally agreed ICHOM standard set. The ICHOM standard set for the field of "arthroplasty" was defined by a global committee of experts, specialists, patients and academics to generate a globally standardized minimal compendium of questions. The following primary data are collected:

Master data and demographic information of the study participants

- Contact details (last name, first name, date of birth, gender, postal address, telephone number, e-mail address (patient), *optional*: e-mail address (relative)
- Identification code (health insurance number, hospital-internal code of the treatment case)
- Demographic information (education level, housing situation, work status, occupation)

The contact data are required to conduct regular follow-up surveys (intervention group) or the final survey at the end of the study period (intervention group and control group). The consent of the study participants to be contacted by e-mail is obtained with the declaration of consent to participate in the study.

The identification codes are used to assign pseudonyms that can be used to link the primary data to the KH case data and KK routine data.

PROMs

- General quality of life (EQ-5D-5L)
- Functional status (KOOS-PS; HOOS-PS)
- Depression and fatigue scales (subscales of the PROMIS 29).
- Analog pain scales

The standardized and validated questionnaires EQ-5D-5L, KOOS-PS and HOOS-PS and the analogous pain scales are components of the ICHOM standard set. The addition of the PROMIS 29 depression and fatigue questionnaires is based on the practical experience of experts consulted and the specialist literature[2] , which show that characteristics in these dimensions have a significant influence on the patient's recovery and are not adequately covered by the EQ-5D-5L.

Clinical variables

- History - findings trauma or injury
- History of previous knee or hip surgeries
- Aftercare (type of rehab, discharge to home or rehab).
- Mobilization of the patient

Transferred from hospital internal OR report/clinical documentation:

- Diagnosis codes (ICD-10-GM)
- Procedure codes (OPS)
- Discharge to home or rehabilitation
- Rehabilitation type
- Operator experience
- Date of the surgery
- Composition of the surgical team
- Duration of the surgery (incision-suture time)
- REF/LOT Number of the implant

Transferred from internal hospital documentation to external inpatient quality assurance (esQS):

- Mobility at discharge
- Fall prevention
- Death during acute inpatient stay
- General complications
- Specific complications
- Ability to walk at discharge
- Duration of the surgical procedure

The clinical variables and demographics are required to form subgroups for risk stratification. Subgroup analyses will further be used to test whether different effects of the intervention are observed depending on certain characteristics. Information from esQS documentation is needed to compare patient-reported PROMs with physician-reported esQS data (e.g., regarding functionality at hospital discharge).

KH case data (secondary data).

- Identification code (first and last name, date of birth, health insurance number, hospital-internal code of the treatment case)
- Other case data (reason for admission, reason for discharge/transfer, etc.)
- Diagnosis information (diagnosis type, diagnosis code, secondary diagnosis, etc.)
- Procedure information (OPS version, localization, etc.)

The hospital case data are based on the data according to § 21 KHEntgG (medical data of the treatment case), which are routinely collected in hospitals in the context of billing to statutory or private health insurers. An extract of these data is transmitted for each study participant who has consented to the processing and linking of these data.

The identification codes are used to assign pseudonyms that can be used to link the KH case data to the primary data.

[2] See, e.g., Singh & Lewallen (2014) Depression in primary TKA and higher medical comorbidities in revision TKA are associated with suboptimal subjective improvement in knee function.

The diagnoses and procedures are required to perform sensitivity analyses (e.g., excluding patients with complicated constellations). The discharge reason is used to identify deaths or cases transferred to post-treatment facilities.

KK routine data (secondary data)

- Identification code (health insurance number)
- Insured person master data (reason for leaving, if applicable)
- Performance data

Service data according to §§ 284, 295, 300, 301, 302 SGB V, as well as § 105 SGB XI according to the data specification from the accounting years 2018 to 2022 are generated as part of the task fulfillment of the statutory health insurance funds. For the purpose of the study, those data will be collected that are available for each participant in the period from October 2019 to September 2021 at his or her health insurance company.

For each study participant who is insured with one of the cooperating health insurance companies and has agreed to the processing and linking of these data, the above-mentioned KK routine data will be transmitted. The identification code is used to assign pseudonyms, which can be used to link the KK routine data with the primary data and KH case data. The performance data are required to examine the cost-effectiveness of the intervention.

Time frame

The data are expected to be made available by health insurers after the completion of the intervention phase in December 2021, with the appropriate time lag in Q3 2022.

Selection criteria

The data of all insured persons named in the assignment list provided by the clinic (see below) are to be included. These insured persons correspond to the patients included in the participating study centers according to the inclusion criteria. Data from 2018 to 2021 will be selected from these insured patients. There are no further inclusion or exclusion criteria.

Assignment list

For selection purposes, a machine-readable assignment list (CSV file) will be provided to health insurers listing study participants whose informed consent has been obtained for the collection of health insurance data.

Data tables

For the selected patients, data from the tables described in the data specification are to be provided. The numbers preceding the names of the tables refer to the legal basis for data transmission according to SGB V. The requested data fields are based on the technical annexes of the corresponding data exchange procedures and their key directories.

In order to merge the data with the primary data of the patients from the participating hospitals and the data of the PROMs, it is necessary to use the patient pseudonym of the assignment table for all supplied data ("PID" field).

Contact details of the post-treatment physician in private practice

- *Contact details (fax number, postal address)*

The contact details of the post-treatment physician are only collected from study participants in the intervention group. This is necessary to be able to inform the responsible treatment provider in case of limit values being exceeded. In case of an unfavorable course detected by regular monitoring, a study information and the reported PROMs will be sent to the post-treatment resident physician either by fax or mail.

The contact data of the post-treatment physician will only be stored in the study centers for the duration of the data collection period; these data will not be transmitted.

5. Description of the data flow

Participating institutions

The following facilities are involved in the data flow:

- Data providers (participating study centers, cooperating health insurers)
- Data receiving agency (aQua institute)
- Research institutions (aQua Institute, MiG Department at TU Berlin)

The participating study centers collect and transmit the primary data and the hospital case data. The study assistant named in the study centers is responsible for this. The study assistant is obligated by the respective participating hospital to maintain confidentiality and secrecy of personal data of the study participants. The transmission of the KK routine data is carried out by the cooperating health insurance companies.

The aQua-Institute receives the pseudonymized primary data and hospital case data directly from the study centers. The KK routine data (previously pseudonymized by the cooperating health insurance funds) are also transmitted directly to the aQua Institute by them.

The research institutions in this project are the aQua Institute and the Department of Healthcare Management at the Technical University of Berlin (MiG). The MiG department receives the three pseudonymized data packages (primary data, KH case data, KK routine data) from the aQua Institute.

Data collection in the study centers

Technical installation for primary data collection

The heartbeat ONE software from HRTBT Medical Solutions GmbH (hereinafter 'heartbeat software') is used for data collection. This allows the study participants and, if applicable, the study assistant to report the PROMs electronically via an input mask. The master data, demographic information and clinical variables listed above are completed by the study assistant.

Heartbeat ONE is operated (depending on the preference of the participating study centers) either by installation in the hospital's IT landscape (on-premise operation) or in an external, ISO 9001 and ISO 27001 certified, TÜV-approved German data center (SaaS operation). This means that each participating hospital has a completely separate and independent instance and database of the heartbeat software.

Data collection at inpatient admission and discharge (PRO survey 1 and 2).

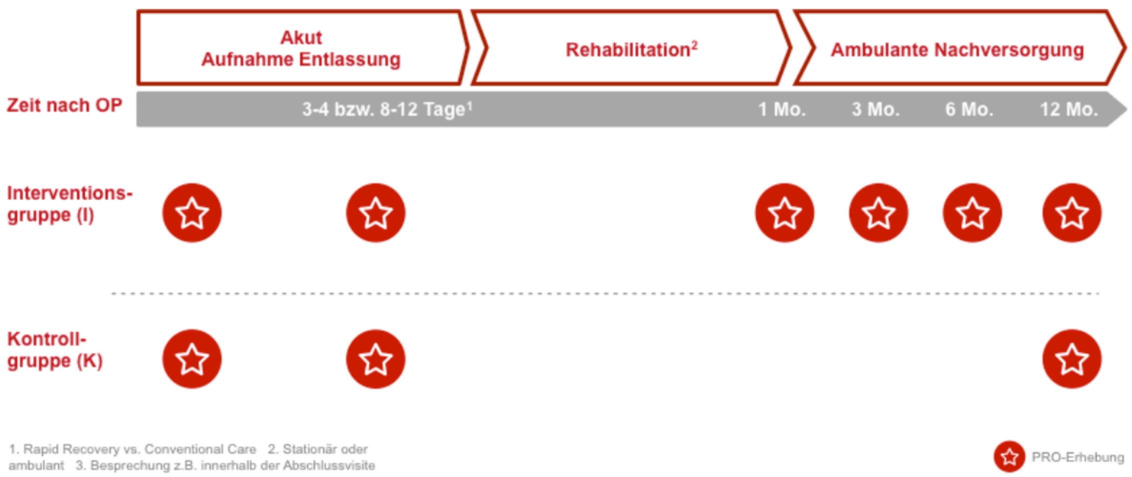

Figure 1: Continuous PROM surveys in intervention group (admission, discharge, follow-up at 1, 3, 6, and 12 months, baseline (admission, discharge), and outcome (follow-up at 12 months) PRO survey in control group.

Primary data collection is performed at admission and discharge at the participating study centers by the patients, supplemented by the study assistance. The study assistance is responsible for recruitment and education of study participants, obtaining informed consent, and data collection and follow-up.

Follow-up data collection (PROM survey after 1, 3, 6 and 12 months).

The follow-up with regular PROM surveys (intervention group) as well as the final survey at the end of the data collection period (intervention group and control group) are triggered automatically by the heartbeat software by requesting the study participant to answer the questionnaire (electronically) via email. The study assistance can be supportive (e.g., telephone) to assist patients in entering the data and to keep the proportion of patients with complete follow-up as high as possible.

Data flow when threshold is exceeded (intervention group only).

In the event of an adverse outcome identified by periodic monitoring, PROMs reported by study participants will be communicated to the patient and, upon consent, to the follow-up office-based physician.

The procedure is as follows:

- The heartbeat software detects an exceedance of the threshold values.
- The study assistant studies the report and initially checks the results for validity (e.g. correct response).
- The study assistant creates a PDF export of the values and sends it password-encrypted by e-mail to the study participant (the patient agrees to this procedure by signing when joining the study).
- The study assistant sends the patient a second e-mail directly afterwards, in which he is informed of the key for the password-protected PDF.
- The study assistant contacts the participant by telephone. The telephone call will be announced in advance in the first e-mail.
- The study assistant then asks the patient whether the physician stored in the system (general practitioner or specialist) is still current and whether the results may be forwarded to him. The verbal consent of the study participant is thus explicitly requested again in order for the study assistant to inform the post-treatment physician. The patient's consent for forwarding is noted in the heartbeat software each time the threshold is exceeded.
- The study assistant sends the participant's report ("one pager") and study information to the post-treatment physician:

  Procedure A: If the post-treatment physician has not changed since discharge and the patient has consented, the fax number is preferred and otherwise the postal address to be used is stored in the heartbeat software or can be determined subsequently by the study assistant.

Procedure B: If the post-treatment physician should have changed since discharge, the study assistance preferably determines his fax number and otherwise the postal address.

Intermediate reports for data collection

The study assistance generates so-called 'Intermediate Reports' from the heartbeat software at the end of each month and transmits them to the MiG department at the TU Berlin. The intermediate reports contain exclusively aggregated data without personal reference (inclusion rate, response rate).

Storage of data in the study centers and deletion periods

In the study centers, the collected primary data are deleted after the end of the evaluation period and the participation and consent forms are destroyed. If, in deviation from this, further processing of the data is planned by the clinic, the clinic will obtain informed consent from the study participants for this. All reading and processing procedures are logged in the database.

If a study participant revokes his/her consent to the processing of data before the end of the data collection period in accordance with Art. 17 DSGVO, the corresponding data will be deleted by the study assistance and no further data will be collected.

In order for the patient to be able to revoke his participation and/or his consent to the linking of the hospital case data and/or his consent to the linking of the KK routine data, a template for this purpose is enclosed with the patient information. The patient can sign this for the purpose of revocation and send it by post to the study assistant responsible for him/her in the hospital.

The deletion of data must be done manually in the system and can be done according to certain filters:

- If the patient has not given consent to the e-mail survey, the autopilot for follow-up surveys cannot be created because the "Consent" field in the master data is answered with "No" by the study assistant.
- Provided that the patient has revoked his consent to the e-mail survey, the autopilot must be deactivated to prevent outgoing e-mails. This is done via a few clicks in the heartbeat software. In the master data, the "Consent" field must be changed to "No".
- If the patient requests the deletion of his or her data, the patient's data must be manually deleted by a heartbeat user with manager rights, specifying the patient's date of birth.
- In the heartbeat software, the status Yes/No/Revocation is stored by the study assistant for each of the consent forms for the hospital case data and KK routine data. The status can be changed manually. The data export can be set so that the status is also exported. Patients with a declaration of consent can then be filtered so that only the information about those patients who have consented to the linking of hospital case data and/or health insurer routine data is forwarded.

The deletion of data that has already been processed is not provided for in accordance with Section 35 of the German Federal Data Protection Act (BDSG), as this would involve a disproportionately high effort.

Data transmission

After completion of the data collection period, the study centers export the data of the study participants collected via Heartbeat ONE and transmit them pseudonymized to the aQua Institute.

The KH case data of the study participants are exported by the study centers after completion of the data collection period. The study centers (either the study assistant or a designated person from controlling) replace the identification tags in the data with the pseudonym automatically generated by the heartbeat software and transmit the pseudonymized data to the aQua institute.

Based on a template, the study assistant generates a list of participants from the software for BITMARCK Service GmbH (which acts as an order processor according to § 80 SGB X for the cooperating company health insurance funds) and BARMER with the identification codes and pseudonyms of the study

participants insured there. It then transmits these lists to these entities by storing them in encrypted form for BITMARCK on the sFTP server of the aQua Institute and for BARMER in its Dracoon Data Room.

BITMARCK and BARMER export the KK routine data for the insured study participants named on the participant list and replace the identification codes with the pseudonyms. The pseudonymized KK routine data are then transmitted to the aQua Institute via the sFTP server (BITMARCK) or the Dracoon Data Room (BARMER). A detailed process description can be found in the appendix.

The aQua Institute thus has at its disposal primary data, KH case data and KK routine data, in each of which there are no longer any identification markers, i.e. person-identifying characteristics. Using the pseudonyms, the data can be linked for analysis for each study participant without the possibility of re-identification. The pseudonymized data are transmitted by the aQua Institute to the Department of Health Care Management at the Technical University of Berlin, which also acts as the researching institution. Both researching institutions carry out the project-related evaluations on a division of labor basis.

Pseudonymization procedure and data linkage

The primary data are pseudonymized in the heartbeat software according to the UUID standard (Universally Unique Identifier). The pseudonym is added to the KH case data and KK routine data records, and the identifiers are subsequently deleted. This allows the data to be linked on a person-by-person basis without the presence of person-identifying characteristics.

Only the pseudonyms are visible to the aQua Institute and the Department of Healthcare Management at the TU Berlin. Re-identification of study participants is thus not possible. The key for pseudonymization is not known to the researching institutions. Only the heartbeat software has the key.

A linkage of primary data, KH case data and KK routine data only takes place for evaluation purposes at the aQua Institute and at the Department of Healthcare Management.

Storage, evaluation duration and deletion periods

The pseudonymized data are stored at the researching institutions for the purpose of analysis. The analysis period begins at the end of the survey period and lasts for two years. In order to allow for verifiability of study results after project completion, data will be retained for a period of 10 years after project completion. This is in accordance with Good Practice Secondary Data Analysis (GPS) recommendations.[3]

Technical procedure for data transmission (transport encryption)

For the implementation of the data transfer

- the participating study centers,
- the BITMARCK and
- the Department of Healthcare Management at the TU Berlin

(hereinafter referred to as "user") is provided with personalized access to the SFTP server of the aQua Institute.

Each user receives a separate drive area on this server where transport-encrypted (AES-256) data can be stored or retrieved. An upload and a download folder are available for this purpose.

For the access, a person is named by each user beforehand (if necessary a substitute), who receives corresponding access data from the aQua-Institute. For additional security, the IP address of the computer used on the user side must be communicated to aQua-Institut.

The access then takes place via access data provided by the aQua institute consisting of: User name, password (communicated by telephone), IP address of the aQua-SFTP server with port specification.

3 See Swart et al. (2015).

Additional explanation of the Data Room from BARMER

DRACOON of "DRACOON GmbH Regensburg" offers a web-based space (operation in Germany), which enables BARMER to exchange data with "clients". There, all data remains maximally protected at all times in compliance with all compliance requirements and data protection guidelines. DRACOON guarantees through end-to-end encryption, including client-side encryption, that the crucial key needed to decrypt the data to be exchanged remains in BARMER's possession. DRACOON is continuously certified and attested, for example with the BSI C5 test certificate or ISO27001.

6. Description of the TOM of the researching institutions

aQua institute

The technical and organizational measures according to Art. 32 DSGVO (Security Manual) can be found in the attachment.

Department of Healthcare Management at the TU Berlin

Person responsible for data protection

Responsibility for data protection lies with Professor Reinhard Busse, project manager and head of the Department of Healthcare Management at the Technical University of Berlin.

Questions should be directed to the Data Protection Officer of Technische Universität Berlin (Annette Hiller, K3-DS).

Memory control

The data is stored on an external hard disk protected by a password. The hard drive is stored in a locked metal cabinet on the premises of the department.

Access control and user control

Data access is only granted to named project participants who are employees of the Department of Healthcare Management at the TU Berlin. A personal user profile with detailed access rights is created for each authorized data user. These access rights are not transferable. The assignment and administration of access rights is the responsibility of the project manager. Access is password-protected and logged.

Input control

All interventions in the evaluation data set in the form of changes, deletions or additions of data are logged.

Recoverability

A backup copy of the raw data set is created. This is stored on a ***password-protected*** data carrier and kept in a separate safe cabinet on the premises of the department.

Data security control

The data analysis is performed on a computer without Internet access. Temporary files generated during the analyses are deleted afterwards. Aggregated results of the analysis are stored on the department's firewall-secured server.

7. Definitions

Primary data are data that are processed and analyzed within the scope of their originally intended use (cf. GPS).

Secondary data are data that are evaluated beyond their original, primary purpose (cf. GPS).

Pseudonymization refers to the processing of personal data in such a way that the data can no longer be assigned to a specific data subject without the addition of additional information, provided that this additional information is stored separately and is subject to technical and organizational measures that ensure that the data cannot be assigned to a data subject (cf. Section 46 BDSG).

8. References

Swart E, Gothe H, Geyer S, Jaunzeme J, Maier B, Grobe TG, Ihle P. Good practice secondary data analysis (GPS): guidelines and recommendations. 3rd version; 2012/2014 version. *health care 2015*; 77(2): 120-26.

Singh J, Lewallen DG. Depression in primary TKA and higher medical comorbidities in revision TKA are associated with suboptimal subjective improvement in knee function. *BMC Musculoskeletal Disorders* 2014; 15:127.

PROMoting Quality

Datenschutzkonzept

Stand: 15.07.2021

Inhaltsverzeichnis

1. Verarbeitungszweck 3
2. Datenschutzrechtliche Prinzipien 3
3. Informierte Einwilligung 3
4. Beschreibung der erhobenen Daten und Begründung der Datenerhebung 4
   Primärdaten 4
   KH-Falldaten (Sekundärdaten) 5
   KK-Routinedaten (Sekundärdaten) 6
   Kontaktdaten des nachbehandelnden niedergelassenen Arztes 7
5. Beschreibung des Datenflusses 7
   Beteiligte Einrichtungen 7
   Datenerhebung in den Studienzentren 7
   Datenübermittlung 10
   Pseudonymisierungsverfahren und Datenverknüpfung 10
   Speicherung, Auswertungsdauer und Löschfristen 10
   Technisches Vorgehen bei der Datenübermittlung (Transportverschlüsselung) 11
6. Beschreibung der TOM der forschenden Einrichtungen 12
   aQua-Institut 12
   Fachgebiet Management im Gesundheitswesen an der TU Berlin 12
7. Begriffsdefinitionen 13
8. Verweise 13

1. Verarbeitungszweck

Bei dem durch den Innovationsfonds des G-BA geförderten Projekt namens „PROMoting Quality - Intersektorale Nutzung von Patient Reported Outcome Measures zur Steigerung der patientenrelevanten Ergebnisqualität" (Kurzbezeichnung PROMoting Quality) handelt es sich um eine randomisierte, kontrollierte, multizentrische Interventionsstudie.

Die Intervention besteht in einem regelmäßigen Monitoring von Patienten[1] durch webbasierte Fragebögen mit einer Laufzeit von bis zu einem Jahr nach elektivem Hüft- oder Kniegelenksersatz unter zu Hilfenahme von Patient Reported Outcome Measures (PROMs).

Ziel der Intervention ist es, patientenrelevante Outcomes (z.B. klinische Ergebnisindikatoren, Funktionalität und Lebensqualität) der Patienten, auch durch Rückspiegelung der Ergebnisse an Patienten und nachbehandelnde Ärzte, positiv zu beeinflussen. Ziel der Studie ist zu prüfen, ob sich in der Interventionsgruppe messbar bessere Outcomes und möglicherweise geringere Behandlungskosten im Vergleich zur Kontrollgruppe beobachten lassen.

Zur Durchführung der Studie werden Primärdaten und Sekundärdaten erhoben, die personenbezogen, aber pseudonymisiert, miteinander verknüpft werden sollen.
Insgesamt werden drei Datenpakete aus unterschiedlichen Quellen erhoben:

- Primärdaten
- KH-Falldaten
- KK-Routinedaten

Bei den Primärdaten handelt es sich um Daten, die spezifisch für den Studienzweck erhoben werden. Bei den weiteren Datenpaketen handelt es sich um routinemäßig in den teilnehmenden Krankenhäusern (N=9) erhobene Daten (KH-Falldaten), sowie um Routinedaten der Krankenkassen (KK-Routinedaten) derjenigen Studienteilnehmer, die bei einer der kooperierenden Krankenkassen (BARMER und dem BKK-Dachverband angehörende Krankenkassen) versichert sind.

Die Datenverarbeitung dient einerseits der Durchführung der Intervention (regelmäßiges Monitoring mit Abfrage von PROMs). Zum anderen ist die Datenverarbeitung erforderlich, um die Effekte der Intervention und den Einfluss von relevanten Behandlungsfaktoren auf die Outcomes zu analysieren und damit das Projektziel zu erreichen.

2. Datenschutzrechtliche Prinzipien

Die Erhebung, Speicherung und Verarbeitung der personenbezogenen Daten in diesem Projekt erfolgt gemäß den jeweils geltenden Datenschutzbestimmungen der Länder in Verbindung mit der Datenschutzgrundverordnung (DSGVO) und unter Berücksichtigung der spezifischen Bestimmungen des Sozialgesetzbuches, Zehntes Buch (SGB X).

3. Informierte Einwilligung

Die wissenschaftliche Nutzung der erhobenen Daten erfolgt auf der Grundlage einer informierten Einverständniserklärung der Studienteilnehmer. Bei der Zielpopulation handelt es sich um Patienten mit einem elektiven, d.h. geplanten Eingriff zum Hüft- oder Kniegelenkersatz. Jedem potentiellen, die Einschlusskriterien erfüllenden Studienteilnehmer wird vor dem Eingriff die Teilnahme an der Studie angeboten. Dabei wird in verständlicher und leicht zugänglicher Form in einer klaren und einfachen

[1] Aus Gründen der besseren Lesbarkeit werden in diesem Dokument Berufs- und Funktionsbezeichnungen stets in der maskulinen Form verwendet. Die Bezeichnungen umfassen jedoch jeweils Personen weiblichen, männlichen und diversen Geschlechts gleichermaßen.

Sprache über die Art der erhobenen Daten, die Datenübermittlung, -verarbeitung, -speicherung und -löschung, sowie die Möglichkeit des Widerrufs informiert.

Auf der Grundlage dieser Informationen können potentielle Teilnehmer eine informierte Entscheidung für oder gegen eine Teilnahme treffen. Damit wird dem Grundsatz einer für die betroffene Person nachvollziehbaren Datenverarbeitung (Artikel 5 DGSVO bzw. § 67b SGB X) Rechnung getragen.

Das informierte Einverständnis wird für die drei Datenpakete separat abgefragt und schriftlich bestätigt:

- Teilnahme an der Studie in Verbindung mit der Erhebung personenbezogener Daten und PROMs (Primärdaten) ***Voraussetzung zum Einschluss in die Studie***
- Erhebung, Verarbeitung und personenbezogene Verknüpfung der Daten des medizinischen Behandlungsfalls im Format gemäß § 21 KHEntgG (KH-Falldaten) ***optional***
- Erhebung, Verarbeitung und personenbezogene Verknüpfung der Routinedaten der Krankenkassen (KK-Routinedaten) für Studienteilnehmer, die bei einer der kooperierenden Krankenkassen versichert sind (gemäß § 67b SGB X) ***optional***

Teilnehmer, die mit der Primärdatenerhebung einverstanden sind, jedoch keine Erhebung, Verknüpfung und Verarbeitung von KH-Falldaten und/oder KK-Routinedaten wünschen, werden ebenfalls in die Studie eingeschlossen, sofern sie sich zur Teilnahme bereit erklärt haben. Das heißt in diesem Fall würden nur Primärdaten erhoben.

Die Einverständniserklärung der Studienteilnehmer über die Studienteilnahme und die Verarbeitung der KH-Falldaten sowie KK-Routinedaten werden in der jeweiligen Klinik aufbewahrt.

4. Beschreibung der erhobenen Daten und Begründung der Datenerhebung

Die Auswahl der zu erhebenden Daten erfolgt im Rahmen der Studienplanung unter dem Grundsatz der Datenminimierung (Artikel 5 DSGVO).

Primärdaten

Die Erhebung der Primärdaten orientiert sich an dem international konsentierten ICHOM-Standardset. Das ICHOM-Standardset für den Bereich „Endoprothetik“ wurde von einem globalen Gremium aus Experten, Fachärzten, Patienten und Akademikern definiert, um eine weltweit standardisiertes minimales Fragenkompendium zu generieren. Es werden die folgenden Primärdaten erhoben:

Stammdaten und demografische Angaben der Studienteilnehmer

- Kontaktdaten (Nachname, Vorname, Geburtsdatum, Geschlecht, Postanschrift, Telefonnummer, E-Mail-Adresse (Patient), *optional*: E-Mail-Adresse (Angehöriger)
- Identifikationskennzeichen (Krankenversichertennummer, krankenhausinternes Kennzeichen des Behandlungsfalls)
- Demografische Angaben (Bildungsstand, Wohnsituation, Arbeitsstatus, Beruf)

Die Kontaktdaten sind erforderlich, um regelmäßige Nachbefragungen (Interventionsgruppe) bzw. die Abschlussbefragung am Ende des Studienzeitraums (Interventionsgruppe und Kontrollgruppe) durchzuführen. Das Einverständnis der Studienteilnehmer zur Kontaktaufnahme per E-Mail wird mit der Einverständniserklärung zur Teilnahme an der Studie eingeholt.

Die Identifikationskennzeichen dienen der Zuordnung von Pseudonymen, anhand derer die Primärdaten mit den KH-Falldaten und KK-Routinedaten verknüpft werden können.

PROMs

- Generelle Lebensqualität (EQ-5D-5L)
- Funktioneller Status (KOOS-PS; HOOS-PS)
- Depressions- und Erschöpfungsskalen (Subskalen des PROMIS 29)
- Analoge Schmerzskalen

Die standardisierten und validierten Fragebögen EQ-5D-5L, KOOS-PS und HOOS-PS und die analogen Schmerzskalen sind Bestandteile des ICHOM-Standardsets. Die Hinzunahme der Depressions- und Erschöpfungsfragebögen des PROMIS 29 erfolgt aufgrund praktischer Erfahrungswerte hinzugezogener Experten und Fachliteratur[2], welche zeigen, dass Ausprägungen in diesen Dimensionen die Genesung des Patienten maßgeblich beeinflussen und durch den EQ-5D-5L nicht ausreichend abgedeckt sind.

Klinische Variablen

- Historie - Befund Trauma oder Verletzung
- Historie von vorherigen OPs an Knie oder Hüfte
- Nachsorge (Art der Reha, Entlassung nach Hause oder in Reha)
- Mobilisierung des Patienten

Übertragen aus klinik-internem OP-Bericht/klinischer Dokumentation:

- Diagnosekodes (ICD-10-GM)
- Prozedurenkodes (OPS)
- Entlassung nach Hause oder in Rehabilitation
- Art der Rehabilitation
- Erfahrung des Operateurs
- Datum der OP
- Zusammensetzung des OP-Teams
- Dauer der OP (Schnitt-Naht-Zeit)
- REF/LOT Nummer des Implantats

Übertragen aus der klinikinternen Dokumentation zur externen stationären Qualitätssicherung (esQS):

- Beweglichkeit bei Entlassung
- Sturzprophylaxe
- Todesfall während des akut-stationären Aufenthaltes
- Allgemeine Komplikationen
- Spezifische Komplikationen
- Gehfähigkeit bei Entlassung
- Dauer des OP-Eingriffs

Die klinischen Variablen und demografischen Angaben sind erforderlich, um Subgruppen für die Risikostratifizierung zu bilden. Durch Subgruppenanalysen soll weiterhin geprüft werden, ob in Abhängigkeit von bestimmten Merkmalen unterschiedliche Effekte der Intervention zu beobachten sind. Die Informationen aus der esQS-Dokumentation sind erforderlich, um die patientenberichteten Angaben der PROMs mit den ärztlich berichteten esQS-Daten zu vergleichen (z.B. bezüglich der Funktionalität bei Entlassung aus dem Krankenhaus).

KH-Falldaten (Sekundärdaten)

- Identifikationskennzeichen (Vor- und Nachname, Geburtsdatum, Krankenversichertennummer, krankenhausinternes Kennzeichen des Behandlungsfalls)
- Weitere Falldaten (Aufnahmeanlass, Entlassungs-/Verlegungsgrund, etc.)
- Diagnoseangaben (Diagnoseart, Diagnoseschlüssel, Sekundär-Diagnose, etc.)
- Prozedurenangaben (OPS Version, Lokalisation, etc.)

---

[2] Siehe z.B. Singh & Lewallen (2014) Depression in primary TKA and higher medical comorbidities in revision TKA are associated with suboptimal subjective improvement in knee function.

Bei den KH-Falldaten handelt es sich um eine Anlehnung an die Daten gemäß § 21 KHEntgG (Medizinische Daten des Behandlungsfalls), die im Rahmen der Rechnungslegung gegenüber gesetzlichen oder privaten Krankenkassen in den Kliniken routinemäßig erhoben werden. Ein Auszug dieser Daten wird für jeden Studienteilnehmer übermittelt, der der Verarbeitung und Verknüpfung dieser Daten zugestimmt hat.

Die Identifikationskennzeichen dienen der Zuordnung von Pseudonymen, anhand derer die KH-Falldaten mit den Primärdaten verknüpft werden können.

Die Diagnosen und Prozeduren sind erforderlich, um Sensitivitätsanalysen (z.B. unter Ausschluss von Patienten mit komplizierten Konstellationen) durchzuführen. Der Entlassungsgrund dient der Identifikation von Todesfällen oder Fällen, die in nachbehandelnde Einrichtungen verlegt werden.

KK-Routinedaten (Sekundärdaten)

- Identifikationskennzeichen (Krankenversichertennummer)
- Versichertenstammdaten (ggf. Austrittsgrund)
- Leistungsdaten

Leistungsdaten nach §§ 284, 295, 300, 301, 302 SGB V, sowie § 105 SGB XI gemäß der Datenspezifikation aus den Abrechnungsjahren 2018 bis 2022 werden im Rahmen der Aufgabenerfüllung der gesetzlichen Krankenkassen generiert. Für den Zweck der Studie werden diejenigen Daten erhoben, die für jeden Teilnehmer im Zeitraum von Oktober 2019 bis September 2021 bei seiner Krankenkasse vorliegen.

Für jeden Studienteilnehmer, der bei einer der kooperierenden Krankenkassen versichert ist und der Verarbeitung und Verknüpfung dieser Daten zugestimmt hat, werden die o.g. KK-Routinedaten übermittelt. Das Identifikationskennzeichen dient der Zuordnung von Pseudonymen, anhand derer die KK-Routinedaten mit den Primärdaten und KH-Falldaten verknüpft werden können. Die Leistungsdaten sind erforderlich, um die Kosteneffektivität der Intervention zu untersuchen.

Zeitrahmen

Die Daten sollen nach Abschluss der Interventionsphase im Dezember 2021 mit der entsprechenden zeitlichen Verzögerung im Q3 2022 durch die Krankenkassen bereitgestellt werden.

Selektionskriterien

Es sollen die Daten aller Versicherten einbezogen werden, die in der von der Klinik bereitgestellten Zuordnungsliste (s.u.) benannt sind. Diese Versicherten entsprechen den in den teilnehmenden Studienzentren entsprechend der Einschlusskriterien eingeschlossen Patienten. Von diesen Versicherten sollen die Daten der Jahre 2018 bis 2021 selektiert werden. Es gibt keine weiteren Ein- oder Ausschlusskriterien.

Zuordnungsliste

Für die Selektion wird den Krankenkassen eine maschinenlesbare Zuordnungsliste (CSV-Datei) bereitgestellt, die die Teilnehmer der Studie, deren Einwilligungserklärung für die Erhebung von Krankenkassendaten vorliegt, auflistet.

Datentabellen

Für die selektierten Patienten sollen Daten der in der Datenspezifikation beschriebenen Tabellen bereitgestellt werden. Die den Namen der Tabellen vorangestellten Ziffern beziehen sich auf die gesetzliche Grundlage zur Datenübermittlung gemäß SGB V. Die angeforderten Datenfelder orientieren sich an den technischen Anlagen der entsprechenden Datenaustauschverfahren und deren Schlüsselverzeichnissen.

Um die Daten mit den Primärdaten der Patienten aus den teilnehmenden Krankenhäusern und den Daten der PROMs zusammenführen zu können, ist es notwendig, das Patientenpseudonym der Zuordnungstabelle für alle gelieferten Daten zu verwenden (Feld „PID").

Kontaktdaten des nachbehandelnden niedergelassenen Arztes

- *Kontaktdaten (Fax-Nummer, Postanschrift)*

Die Kontaktdaten des nachbehandelnden Arztes werden nur bei Studienteilnehmern der Interventionsgruppe erhoben. Dies ist erforderlich, um im Falle der Überschreitung von Grenzwerten den zuständigen Behandler informieren zu können. Im Falle eines durch das regelmäßige Monitoring festgestellten ungünstigen Verlaufes werden eine Studieninformation und die berichteten PROMs an den nachbehandelnden niedergelassenen Arzt entweder per Fax oder postalisch übermittelt.

Die Kontaktdaten des nachbehandelnden Arztes werden nur in den Studienzentren für die Dauer des Erhebungszeitraumes gespeichert, eine Übermittlung dieser Daten findet nicht statt.

5. Beschreibung des Datenflusses

Beteiligte Einrichtungen

Folgende Einrichtungen sind am Datenfluss beteiligt:

- Datenliefernde Stellen (Teilnehmende Studienzentren, kooperierende Krankenkassen)
- Datenannehmende Stelle (aQua-Institut)
- Forschende Einrichtungen (aQua-Institut, Fachgebiet MiG an der TU Berlin)

Die teilnehmenden Studienzentren erheben und übermitteln die Primärdaten sowie die KH-Falldaten. Verantwortlich dafür ist jeweils die in den Studienzentren namentlich benannte Studienassistenz. Die Studienassistenz wird durch die jeweilige teilnehmende Klinik zur Verschwiegenheit und Geheimhaltung persönlicher Daten der Studienteilnehmer verpflichtet. Die Übermittlung der KK-Routinedaten erfolgt durch die kooperierenden Krankenkassen.

Das aQua-Institut erhält die pseudonymisierten Primärdaten und KH-Falldaten direkt von den Studienzentren. Auch die (zuvor von den kooperierenden Krankenkassen pseudonymisierten) KK-Routinedaten werden von diesen direkt an das aQua-Institut übermittelt.

Forschende Einrichtungen in diesem Projekt sind das aQua-Institut sowie das Fachgebiet Management im Gesundheitswesen an der TU Berlin (MiG). Das Fachgebiet MiG erhält die drei pseudonymisierten Datenpakete (Primärdaten, KH-Falldaten, KK-Routinedaten) vom aQua-Institut.

Datenerhebung in den Studienzentren

Technische Installation zur Primärdatenerhebung

Für die Datenerhebung wird die Software heartbeat ONE der HRTBT Medical Solutions GmbH (im Folgenden ‚heartbeat-Software‘) eingesetzt. Damit können die Studienteilnehmer und ggf. die Studienassistenz die PROMs über eine Eingabemaske elektronisch berichten. Die oben aufgeführten Stammdaten, demografischen Angaben und klinischen Variablen werden von der Studienassistenz ergänzt.

Der Betrieb von Heartbeat ONE erfolgt (je nach Präferenz der teilnehmenden Studienzentren) entweder durch Installation in der IT-Landschaft des Krankenhauses (onPremise Betrieb) oder in einem externen, nach ISO 9001 und ISO 27001 zertifizierten, TÜV-geprüften, deutschen Rechenzentrum (SaaS Betrieb). Damit verfügt jedes teilnehmende Krankenhaus über eine vollständig separierte und eigenständige Instanz und Datenbank der heartbeat-Software.

Datenerhebung bei stationärer Aufnahme und Entlassung (PRO-Erhebung 1 und 2)

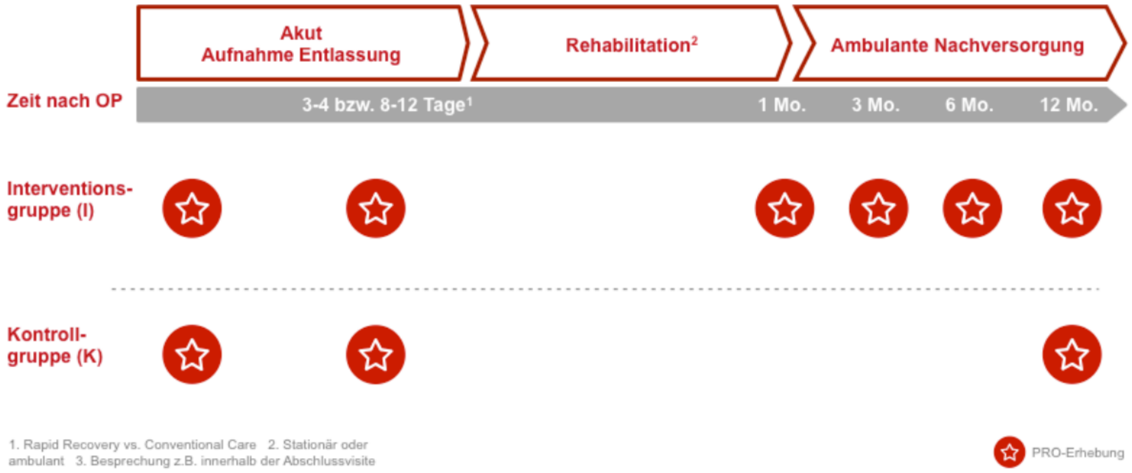

Abbildung 1: Kontinuierliche PROM-Erhebungen bei Interventionsgruppe (Aufnahme, Entlassung, Follow-up nach 1, 3, 6 und 12 Monaten, Baseline (Aufnahme, Entlassung) und Outcome (Follow-up nach 12 Monaten) PRO-Erhebung bei Kontrollgruppe.

Die Erhebung der Primärdaten erfolgt bei der Aufnahme und der Entlassung in den teilnehmenden Studienzentren durch die Patienten, ergänzt von der Studienassistenz. Die Studienassistenz ist für die Rekrutierung und Aufklärung der Studienteilnehmer, das Einholen der Einverständniserklärung sowie die Datenerhebung und Nachverfolgung verantwortlich.

Follow-up Datenerhebung (PROM-Erhebung nach 1, 3, 6 und 12 Monaten)

Die Nachverfolgung mit regelmäßigen PROM-Erhebungen (Interventionsgruppe) sowie die Endbefragung am Ende des Erhebungszeitraumes (Interventionsgruppe und Kontrollgruppe) werden automatisiert durch die heartbeat-Software ausgelöst, indem der Studienteilnehmer per E-Mail zur (elektronischen) Beantwortung des Fragebogens aufgefordert wird. Die Studienassistenz kann unterstützend tätig werden (z.B. Telefon), um Patienten bei der Eingabe der Daten zu unterstützen und den Anteil der Patienten mit vollständiger Nachverfolgung möglichst hoch zu halten.

Datenfluss bei Schwellenwertüberschreitung (nur Interventionsgruppe)

Im Falle eines durch das regelmäßige Monitoring festgestellten ungünstigen Verlaufes werden die von Studienteilnehmern berichteten PROMs an den Patienten und nach Zustimmung an den nachbehandelnden niedergelassenen Arzt übermittelt.

Der Ablauf gestaltet sich wie folgt:

- Die heartbeat-Software stellt eine Überschreitung der Schwellenwerte fest.
- Die Studienassistenz studiert den Report und überprüft die Ergebnisse initial auf Validität (z.B. korrekte Beantwortung).
- Die Studienassistenz stellt einen PDF-Export der Werte her und versendet diesen passwortverschlüsselt per E-Mail an den Studienteilnehmer (der Patient stimmt diesem Vorgehen bei Teilnahmeeinschluss durch seine Unterschrift zu).
- Die Studienassistenz schickt dem Patienten direkt im Anschluss eine zweite E-Mail, in welcher ihm der Schlüssel für das passwortgeschützte PDF mitgeteilt wird.
- Die Studienassistenz kontaktiert den Teilnehmer telefonisch. Das Telefonat wird vorab in der ersten E-Mail angekündigt.
- Dann erfragt die Studienassistenz beim Patienten, ob der im System hinterlegt Arzt (Hausarzt oder Facharzt) noch aktuell ist, und ob die Ergebnisse an ihn weitergeleitet werden dürfen. Für die Unterrichtung des nachbehandelnden Arztes durch die Studienassistenz wird somit explizit das mündliche Einverständnis des Studienteilnehmers erneut erfragt. Das Einverständnis des Patienten für die Weiterleitung wird in der heartbeat-Software bei jeder Schwellenwertüberschreitung vermerkt.

– Dem nachbehandelnden Arzt schickt die Studienassistenz den Report des Teilnehmers ("One Pager") sowie eine Studieninformation zu:

  Vorgehen A: Wenn der nachbehandelnde Arzt sich seit Entlassung nicht geändert und der Patient zugestimmt hat, ist präferiert die Fax-Nummer und ansonsten die zu verwendende Post-Adresse in der heartbeat-Software hinterlegt oder nachträglich durch die Studienassistenz zu ermitteln.

  Vorgehen B: Wenn sich der nachbehandelnde Arzt seit Entlassung geändert haben sollte, ermittelt die Studienassistenz präferiert dessen Fax-Nummer und ansonsten die Post-Adresse.

Intermediate Reports zur Datenerhebung

Die Studienassistenz erzeugt aus der heartbeat-Software zum Ende eines jeden Monats sogenannte ‚Intermediate Reports' und übermittelt diese an das Fachgebiet MiG an der TU Berlin. Die Intermediate Reports enthalten ausschließlich aggregierte Daten ohne Personenbezug (Einschlussquote, Rücklaufquote).

Speicherung der Daten in den Studienzentren und Löschfristen

In den Studienzentren werden die erhobenen Primärdaten nach Ablauf des Auswertungszeitraumes gelöscht und die Teilnahme- und Einverständniserklärungen vernichtet. Sofern abweichend davon seitens der Klinik eine weitere Verarbeitung der Daten vorgesehen ist, holt die Klinik dafür eine informierte Einwilligung der Studienteilnehmer ein. Alle Lese- und Bearbeitungsvorgänge werden in der Datenbank protokolliert.

Sofern ein Studienteilnehmer gemäß Art. 17 DSGVO vor Ablauf des Erhebungszeitraumes sein Einverständnis zur Verarbeitung der Daten widerruft, werden die entsprechenden Daten durch die Studienassistenz gelöscht und keine weiteren Daten erhoben.

Damit der Patient seine Teilnahme und/oder sein Einverständnis zur Verknüpfung der KH-Falldaten und/oder sein Einverständnis zur Verknüpfung der KK-Routinedaten widerrufen kann, wird der Patienteninformation eine Vorlage dazu beigelegt. Diese kann der Patient zwecks Widerrufes unterzeichnen und an die für ihn zuständige Studienassistenz in der Klinik postalisch zuschicken.

Die Löschung der Daten ist manuell im System vorzunehmen und kann nach bestimmten Filtern erfolgen:

- Sofern der Patient keine Einwilligung zur E-Mail-Befragung abgegeben hat, kann der Autopilot für Follow-Up Befragungen nicht angelegt werden, da in den Stammdaten das Feld „Einwilligung" mit „Nein" durch die Studienassistenz beantwortet wird.
- Sofern der Patient seine Einwilligung zur E-Mail-Befragung widerrufen hat, muss der Autopilot deaktiviert werden, um ausgehende E-Mails zu verhindern. Dies geschieht über wenige Klicks in der heartbeat-Software. In den Stammdaten muss das Feld „Einwilligung" in „Nein" umgeändert werden.
- Sofern der Patient die Löschung seiner Daten beantragt, müssen die Daten des Patienten von einem heartbeat-User mit Manager-Rechten unter Angabe des Geburtsdatums des Patienten manuell gelöscht werden.
- In der heartbeat-Software wird für die Einverständniserklärungen zu den KH-Falldaten und KK-Routinedaten je der Status Ja/Nein/Widerruf durch die Studienassistenz hinterlegt. Der Status ist manuell änderbar. Der Datenexport lässt sich so einstellen, dass der Status mitexportiert wird. Danach lassen sich Patienten mit einer Einverständniserklärung filtern und so wird nur die Information über diejenigen Patienten weitergeleitet, die einer Verknüpfung von KH-Falldaten und/oder KK-Routinedaten zugestimmt haben.

Die Löschung bereits verarbeiteter Daten ist in Orientierung an § 35 BDSG nicht vorgesehen, da hierdurch ein unverhältnismäßig hoher Aufwand entstehen würde.

Datenübermittlung

Nach Abschluss des Erhebungszeitraums werden von den Studienzentren die über Heartbeat ONE erhobenen Daten der Studienteilnehmer exportiert und pseudonymisiert an das aQua-Institut übermittelt.

Die KH-Falldaten der Studienteilnehmer werden nach Abschluss des Erhebungszeitraums von den Studienzentren exportiert. Die Studienzentren (entweder die Studienassistenz oder eine benannte Person des Controllings) ersetzen die Identifikationskennzeichen in den Daten durch das automatisch von der heartbeat-Software generierte Pseudonym und übermitteln die pseudonymisierten Daten an das aQua-Institut.

Die Studienassistenz erzeugt aus der Software basierend auf einem Template jeweils eine Teilnehmerliste für die BITMARCK Service GmbH (welche als Auftragsverarbeiter nach § 80 SGB X für die kooperierenden Betriebskrankenkassen agiert) und die BARMER mit den Identifikationskennzeichen und Pseudonymen der dort versicherten Studienteilnehmer. Daraufhin übermittelt sie diese Listen an diese Stellen, indem sie sie verschlüsselt für die BITMARCK auf dem sFTP-Server des aQua-Institutes und für die BARMER in deren Dracoon Data Room ablegt.

BITMARCK und BARMER exportieren für die auf der Teilnehmerliste genannten, versicherten Studienteilnehmer die KK-Routinedaten und ersetzen die Identifikationskennzeichen durch die Pseudonyme. Die pseudonymisierten KK-Routinedaten werden anschließend über den sFTP-Server (BITMARCK), bzw. den Dracoon Data Room (BARMER) an das aQua-Institut übermittelt. Eine detaillierte Prozessbeschreibung befindet sich im Anhang.

Das aQua-Institut verfügt damit über Primärdaten, KH-Falldaten und KK-Routinedaten, in denen jeweils keine Identifikationskennzeichen, d.h. personenidentifizierende Merkmale mehr vorhanden sind. Über die Pseudonyme können die Daten zur Auswertung für jeden Studienteilnehmer verknüpft werden, ohne dass eine Re-Identifizierung möglich ist. Die pseudonymisierten Daten werden vom aQua-Institut an das Fachgebiet Management im Gesundheitswesen an der TU Berlin, das ebenfalls als forschende Einrichtung fungiert, übermittelt. Beide forschenden Einrichtungen führen arbeitsteilig die projektbezogenen Auswertungen durch.

Pseudonymisierungsverfahren und Datenverknüpfung

Die Primärdaten werden in der heartbeat-Software nach dem UUID Standard (Universally Unique Identifier) pseudonymisiert. Das Pseudonym wird den Datensätzen KH-Falldaten und KK-Routinedaten hinzugefügt, die Identifikationskennzeichen werden anschließend gelöscht. Damit können die Daten personenbezogen verknüpft werden, ohne dass personenidentifizierende Merkmale vorhanden sind.

Für das aQua-Institut und das Fachgebiet Management im Gesundheitswesen an der TU Berlin sind nur die Pseudonyme sichtbar. Eine Re-Identifikation von Studienteilnehmer ist damit nicht möglich. Der Schlüssel zur Pseudonymisierung ist den forschenden Einrichtungen nicht bekannt. Nur die heartbeat-Software verfügt über den Schlüssel.

Eine Verknüpfung von Primärdaten, KH-Falldaten und KK-Routinedaten findet nur für Auswertungszwecke beim aQua-Institut und am Fachgebiet Management im Gesundheitswesen statt.

Speicherung, Auswertungsdauer und Löschfristen

Die pseudonymisierten Daten werden zum Zweck der Analyse bei den forschenden Einrichtungen gespeichert. Der Analysezeitraum beginnt mit dem Ende des Erhebungszeitraums und beträgt zwei Jahre. Um nach Abschluss des Projektes eine Überprüfbarkeit der Studienergebnisse zu ermöglichen, werden die Daten für einen Zeitraum von 10 Jahren nach Projektabschluss aufbewahrt. Dies entspricht den Empfehlungen der Guten Praxis Sekundärdatenanalyse (GPS).[3]

[3] Siehe Swart et al. (2015).

Technisches Vorgehen bei der Datenübermittlung (Transportverschlüsselung)

Zur Durchführung der Datenübermittlung wird

- den teilnehmenden Studienzentren,
- der BITMARCK und
- dem Fachgebiet Management im Gesundheitswesen an der TU Berlin

(im folgenden „Nutzer" genannt) jeweils ein personalisierter Zugang zum SFTP-Server des aQua-Instituts zu Verfügung gestellt.

Jeder Nutzer erhält dazu auf diesem Server einen separaten Laufwerksbereich auf dem transportverschlüsselt (AES-256) Daten abgelegt, bzw. abgeholt werden können. Dazu stehen ein Upload- und ein Download-Ordner zur Verfügung.

Für den Zugang wird von jedem Nutzer zuvor eine Person benannt (ggf. eine Vertretung), die vom aQua-Institut entsprechende Zugangsdaten erhält. Zur zusätzlichen Absicherung muss die IP-Adresse des Rechners, die auf Nutzerseite verwendet wird, dem aQua-Institut mitgeteilt werden.

Der Zugang erfolgt dann über vom aQua-Institut bereitgestellte Zugangsdaten bestehend aus: Benutzername, Passwort (wird telefonisch mitgeteilt), IP-Adresse des aQua-SFTP-Servers mit Port-Angabe.

Zusätzliche Erläuterung des Data Rooms von der BARMER

DRACOON der „DRACOON GmbH Regensburg“ bietet einen webbasierten Raum (Betrieb in Deutschland), welcher der BARMER den Datenaustausch mit „Clienten“ ermöglicht. Dort bleiben alle Daten unter Einhaltung sämtlicher Compliance-Vorgaben und Datenschutz-Richtlinien zu jedem Zeitpunkt maximal geschützt. DRACOON garantiert durch eine Ende-zu-Ende-Verschlüsselung inkl. clientseitiger Verschlüsselung, dass der entscheidende Schlüssel, der benötigt wird, um die auszutauschenden Daten wieder entschlüsseln zu können, im Besitz der BARMER bleibt. DRACOON wird laufend zertifiziert und testiert, beispielsweise mit dem BSI C5 Testat oder der ISO27001.

6. Beschreibung der TOM der forschenden Einrichtungen

aQua-Institut

Die technischen und organisatorischen Maßnahmen gem. Art. 32 DSGVO (Sicherheitshandbuch) finden sich in der Anlage.

Fachgebiet Management im Gesundheitswesen an der TU Berlin

Verantwortliche für den Datenschutz

Die Verantwortung für den Datenschutz liegt bei Professor Reinhard Busse, Projektleiter und Leiter des Fachgebietes Management im Gesundheitswesen an der TU Berlin.

Fragen sind an die Behördliche Datenschutzbeauftragte der Technischen Universität Berlin (Annette Hiller, K3-DS) zu richten.

Speicherkontrolle

Die Daten werden auf einer externen Festplatte passwortgeschützt gespeichert. Die Festplatte wird in einem verschlossenen Metallschrank in den Räumen des Fachgebietes aufbewahrt.

Zugriffskontrolle und Benutzerkontrolle

Ein Datenzugriff erfolgt nur durch namentlich benannte Projektbeteiligte, die Mitarbeiter des Fachgebietes Management im Gesundheitswesen an der TU Berlin sind. Für jeden berechtigten Datennutzer wird ein persönliches Nutzerprofil mit detaillierten Zugriffsrechten erstellt. Diese Zugriffsrechte sind nicht übertragbar. Die Vergabe und Verwaltung von Zugriffsrechten liegt in der Verantwortung des Projektleiters. Zugriffe erfolgen passwortgeschützt und werden protokolliert.

Eingabekontrolle

Alle Eingriffe in den Auswertungsdatensatz in Form von Änderungen, Löschen oder Hinzufügen von Daten werden protokolliert.

Wiederherstellbarkeit

Es wird eine Sicherungskopie des Rohdatensatzes erstellt. Diese wird auf einem Datenträger passwortgeschützt gespeichert und in einem separaten Tresorschrank in den Räumen des Fachgebietes aufbewahrt.

Datensicherheitskontrolle

Die Datenauswertung erfolgt auf einem Rechner ohne Internetzugang. Temporäre Dateien, die während der Analysen erzeugt werden, werden anschließend gelöscht. Aggregierte Ergebnisse der Analyse werden auf dem Firewall-gesicherten Server des Fachgebietes gespeichert.

7. Begriffsdefinitionen

Primärdaten sind Daten, die im Rahmen ihres originär vorgesehenen Verwendungszwecks aufbereitet und analysiert werden (vgl. GPS).

Sekundärdaten sind Daten, die einer Auswertung über ihren originären, vorrangigen Verwendungszweck hinaus zugeführt werden (vgl. GPS).

Pseudonymisierung bezeichnet die Verarbeitung personenbezogener Daten in einer Weise, in der die Daten ohne Hinzuziehung zusätzlicher Informationen nicht mehr einer spezifischen betroffenen Person zugeordnet werden können, sofern diese zusätzlichen Informationen gesondert aufbewahrt werden und technischen und organisatorischen Maßnahmen unterliegen, die gewährleisten, dass die Daten keiner betroffenen Person zugewiesen werden können (vgl. § 46 BDSG).

8. Verweise

Swart E, Gothe H, Geyer S, Jaunzeme J, Maier B, Grobe TG, Ihle P. Gute Praxis Sekundärdatenanalyse (GPS): Leitlinien und Empfehlungen. 3. Fassung; Version 2012/2014. *Gesundheitswesen 2015*; 77(2): 120-26.

Singh J, Lewallen DG. Depression in primary TKA and higher medical comorbidities in revision TKA are associated with suboptimal subjective improvement in knee function. *BMC Musculoskeletal Disorders* 2014; 15:127.

Subscribe to DeepL Pro to translate larger documents.
Visit www.DeepL.com/pro for more information.

Technische Universität Berlin

ZUKUNFT DURCH QUALITÄT

24 . September 2020

PROMoting Quality - Intersectoral use of Patient Reported Outcome Measures to increase patient-relevant outcome quality.

Funding code 01NVF18016

Evaluation concept V4

Responsible methodologist and contact person for the evaluation concept:
Thomas G. Grobe, MD, MPH

aQua - Institute for Applied Quality Promotion and
Research in Health Care GmbH Maschmühlenweg 8-
10 - 37073 Göttingen
Phone (+49) 0551-789 52 - 139
thomas.grobe@aqua-institut.de

Background

This document contains a compilation of different versions of the evaluation concept. Contents of the first version of the evaluation concept (version 1, 08 January 2019), the revised version (version 2, 28 May 2019), the third version (version 3, 27 November 2019), which were further supplemented in the present fourth version (version 4, 18 September 2020), were taken into account.

To simplify readability, only the final target or expected case numbers are listed in this version (exceptions exist where it was purely a matter of the funding commitment from the unblocking application of the originally requested case number). Changes in the number of cases compared to previous versions of the evaluation concept can be explained by changes in the number of participating hospitals (e.g. Tabea Hospital withdrew from the study, which was compensated by the addition of RoMed Kliniken and Waldklinikum Eisenberg) and by recruitment losses due to the COVID-19 pandemic.

Since the estimates of the number of cases in the project are based on certain assumptions and on a methodology that is as comprehensible as possible, changes in the general conditions inevitably lead to changes in the expected number of cases. From the point of view of those involved in the study, it must first and foremost be ensured that the originally targeted case numbers are not undercut by changes in the framework conditions and can therefore jeopardize the study. At this point, it should be explicitly pointed out that higher case numbers tend to improve the study's informative value. Since the higher case numbers are realized cost-neutrally within the framework of the study and negative effects of the study intervention on the health of patients can be ruled out with a very high degree of probability, no disadvantages are to be expected with an increase in case numbers.

Components version 1

In the notification of the Innovation Committee at the G-BA dated 19.10.2018, it was stated as a first requirement for the implementation of the project: *A more detailed evaluation concept is to be submitted, which in particular presents the methodology, the intervention and control group, and the case number planning in more detail. The approach to avoid bias is to be explained. The number of cases for the health care services must be derived from the evaluation concept.*

During a telephone conversation between the applicants and the project executing agency on December 4, 2018, these statements were substantiated. In the minutes of the telephone conversation, the project executing agency lists the following three sub-items under "2. evaluation concept":

- *A more detailed evaluation concept with the signature of the responsible methodologist must be submitted, which in particular presents the methodology, the intervention and control group as well as the case number planning in more detail.*
- *It is to be considered that a selection bias can arise due to the e-mail contact. Please explain the procedure to avoid a bias.*
- *The number of cases for the health care services must be derived from the evaluation concept. However, the case number for the health care services includes significantly more persons (4,908) than would be required by the evaluation criteria (698) (factor 7). This contradiction is to be dissolved please.*

The following explanations present the evaluation concept of the PROMoting Quality project in more detail - with recourse to text passages from the original application - whereby both strategies for the avoidance of bias are dealt with and, in particular, comprehensible justifications for the targeted case numbers are to be provided.

At the same time, the explanations are intended to make clear that *"the care services provided are also included in the scientific monitoring and evaluation", which is stated in the protocol of the telephone call in the section under "Health Care Services"* is mentioned as a requirement for funding of related services.

Components version 2 and 3

For the explanations, we have followed the sequence of version 1 of the concept and used the additional questions - where appropriate in terms of content - under the respective headings from the funding decision (dated 29.03.19), the amendment decision (dated 05.08.19) and the additional requirements (dated 24.10.19) *highlighted in gray* as a starting point for the answers.

Components version 4

The current version contains both new assumptions on the expected case numbers due to the changed COVID-19 framework and explanations on their impact on the planned evaluations. Furthermore, a modified evaluation plan resulting from the extended recruitment period is explained.
The updated information is marked in green font color.

Care goals, intervention, and target population

The intervention within the framework of PROMoting Quality aims - in general terms - to positively influence the health development and quality of life of patients over time after major medical interventions/interventions. The gain in health and quality of life is to be realized cost-effectively, i.e. without a disproportionate increase in the resources expended, which can lead to savings in the utilization of health care services in the medium term. In addition to determining the effects of the intervention, the project also aims to demonstrate the practicability of the intervention procedure, to identify approaches for optimizing the procedure, and to assess the transferability of the results and the effects of extending the intervention to all patients with the target interventions under consideration in Germany.

The **intervention** initially consists of regular monitoring of patients over time after major medical interventions with the aid of Patient Reported Outcome Measures (PROMs). The aim is to identify complications and unfavorable developments at an early stage and to treat them more effectively. In the case of unfavorable events and developments, a study nurse will then notify the attending specialist, if necessary in relation to threshold values, and on this basis provide closer and more individualized care in the sense of adapting the follow-up treatment to the situation.

The **target population** for a corresponding intervention within the framework of PROMoting Quality are patients with an elective hip or knee replacement. In principle, the intervention could also be applied to other medical interventions. The decisive factor for a restriction to exactly this patient group is the expectation that within a limited period of time, involving a still manageable number of centers, a sufficient number of patients with comparatively homogeneous characteristics can be recruited. Only under these conditions can the practical implementation of the planned intervention and its evaluation be guaranteed within a limited study period with limited resources.

Outcomes relevant to evaluation

In the evaluation of intervention effects, PROMoting Quality takes into account different characteristics and derived parameters, which are recorded in different ways.

According to the project proposal, the **primary outcome** is the "outcome over cost ratio".

Secondary outcomes assessed are:

- Functional status (assessed via HOOS-PS / KOOS-PS score),
- Quality of life (assessed via EQ-5D-3L score),
- Pain in the hip, knee or lower back,
- Work Status,
- general satisfaction with treatment success,
- Clinical outcome indicators such as postoperative readmission rate, reoperation rate.

The "outcome over cost ratio" represents the relevant goal of the intervention - the cost-effective gain in health and quality of life without a disproportionate increase in resources expended - in a single metric and was defined as the primary outcome in light of this.

Nevertheless, the secondary outcomes and, in particular, the inclusion of the patient's perspective within the framework of PROMoting Quality also play an important role in the evaluation of the success of the intervention. All secondary outcomes should be influenced by the intervention in a positive sense, whereby negative effects should at least be excluded with a high degree of probability during the evaluation.

Study design (RCT) and study procedure

PROMoting Quality is a multicenter randomized controlled intervention trial (RCT) in the classical sense. The randomized assignment of patients to the control or intervention arm of the study will be software-controlled and uninfluenced by the treating persons at the level of the individual patients, whereby patients assigned to both the control and intervention groups will be recruited equally from all partner clinics (see below).

A special feature of the research design is that for subgroups of patients from both the intervention and the control group, in the case of insurance with the health insurance funds involved in the project, BARMER and the BKK umbrella organization, the service and billing data are also evaluated in the sense of a 'Value Based Health Care' (VBHC) approach in addition to the quality of outcomes. The proportion of these patients, whose data are primarily included in the evaluation of the primary endpoint, in the total population studied is expected to be just under one third (29.06%). Nevertheless, all collected data, i.e. data of the 'VBHC patients' as well as data of the PROM-only patients (without the availability of service and billing data) will find their way into the scientific monitoring and evaluation.

Inclusion and exclusion criteria
Patients with target interventions will be fully recruited within a 12-month period as part of the study. The recruited patients with an assignment to the control or intervention group will then each be followed up over a period of 12 months from the day of surgery/target intervention. A total of nine hospitals with different locations in Hamburg, Schleswig-Holstein, Lower Saxony, Bavaria, Berlin and Brandenburg are involved as partner hospitals in PROMoting Quality.

- **Inclusion criterion** for study participation is the elective performance of a first-time joint replacement of the hip or knee in one of the partner hospitals within the recruitment period (procedures with the operation and procedure codes (OPS) 5-820 "Implantation of an endoprosthesis of the hip joint" and 5-822 "Implantation of an endoprosthesis of the knee joint".

- Patients who are younger than 18 years of age at hospital admission, who have had previous joint replacement at the same joint, who are undergoing emergency surgery (e.g., after near-hip femur fracture), and patients in the ASA-Classes 4 to 6 (patient with life-threatening illness; moribund patient unlikely to survive without surgery; deceased patient with established brain death, organ donor).

  In addition, patients will be excluded from study participation if they cannot be reached directly or indirectly via e-mail for the duration of the study and if they do not have access to the Internet.

Randomization
The randomized assignment of suitable patients (according to inclusion criterion and without one of the mentioned exclusion criteria) to the intervention or control group will be done after a consent for study participation in the baseline PROM survey, which is obligatory for both groups in the course of the study. The assignment will be implemented in such a way that it will take place without any influence by the treating physician or the patient. Patients will be randomly assigned to either the intervention or the control group in all partner clinics with equal probabilities, which should result in approximately the same number of intervention and control group patients per clinic and overall.

From the point of view of the study participants, the described procedure ensures that study participants are assigned to the intervention or control group in the best sense of an RCT - and apart from deviations due to chance - without any bias. The patients initially assigned to the intervention or control group in the baseline PROM survey form the starting point for all subsequent analyses, whereby evaluations including precisely these patients, who were selected prior to any intervention that may take place, can also be referred to as intention-to-treat analyses. With regard to the subgroup of patients insured by BARMER and health insurance companies belonging to the BKK umbrella organization, the later availability of unbiased data can be expected within the framework of PROMoting Quality until the end of the planned observation period one year after the surgical joint replacement, also independent of the further participation of the patients in the PROM survey.

Primary collection of data (especially PROMs).
For all patients with inclusion criteria and no reasons for exclusion, a baseline PROM survey will be performed during inpatient admission and after written informed consent for study participation using the Heartbeat ONE software, i.e. before the surgical procedure at the partner hospital.

Exclusively in the intervention group, four additional software-supported PROM surveys are performed, namely one day before discharge and regularly also in months 1, 3, and 6 after surgery.

A new and final PROM survey of all patients included in the study is planned 12 months after performance of the target intervention. It is assumed that after appropriate use of the study nurses, results of the final PROM survey can be recorded for about 70% of the study participants.

The management of PROM results by study nurses includes manual follow-up of missing PROM data, training of patients with the PROM survey tool Heartbeat ONE on admission and before discharge (only for intervention group I), complementing the ICHOM standard set with joint-specific medical history (past indications and (surgical) procedures) and clinical outcome quality indicators, documentation of variables from the OR report (team composition, experience of the surgeon and implant choice) and the §21 KHEntgG data (inpatient length of stay, discharge type/reason) as well as initial review of PROM results, forwarding of critical PROM results to relevant institutions (in any case to treating specialist).

To support the PROM analysis, conspicuous PROM results are displayed with priority by the Heartbeat ONE software. Conspicuous PROM results are defined as exceeding defined critical values and/or a continuous deterioration in values. Based on the forwarded abnormal PROM results, the attending specialist can order (additional) examinations or interventions to remedy the identified symptoms and complaints.

Secondary use of data by health insurers (especially costs)
Data routinely collected from health insurers are used in particular to determine the costs of health care for study participants, but also to estimate the transferability of study results to nationwide care. Within the framework of PROMoting Quality, the BARMER and the BKK Dachverband could be won as cooperation partners for the provision of corresponding data. Assessments of the transferability of study results are primarily based on data from BARMER. It is estimated that about 30% of the German population is insured by BARMER and the BKK Dachverband, which means that routine data on more than a quarter of all study participants can be used within the framework of PROMoting Quality.

Additional health insurers could not be persuaded to participate in the project in the run-up to the study planning. In any case, the inclusion of additional health insurance funds would have increased the costs incurred by the funds. At the same time, the coordination effort as well as the effort for data preparation would have increased considerably in the course of the evaluation, since different data warehouse solutions are used by the health insurance funds.

Expected patient numbers and study participation
All clinics were asked to specify their case numbers according to the exclusion criteria (patients under 18 years, fractures, and patients in ASA classes 4-6). This allows inclusion of only those patients with desired inclusion criteria in the evaluation.

However, the expected patient numbers had to be corrected during the preparation period.

- The reasons for this were changes in the sample of clinics: Two clinics (Asklepios Westklinikum Hamburg and Tabea Krankenhaus) cancelled their participation, but two new clinics joined (RoMed Klinik Prien a. Chiemsee and Waldkliniken Eisenberg).
- In addition, the expected patient numbers had to be adjusted again due to the special situation of the COVID-19 lockdown and the associated suspension of planned and scheduling of new hip and knee arthroplasty surgeries. Hospitals were unable to perform these generally elective surgeries in a timely manner due to the provision of resources in the area of intensive care beds for patients suffering from COVID-19, as well as risk minimization with respect to infection of particularly vulnerable age groups (the average age of the study population is > 68 years[1,2]). This led to a considerable collapse in recruitment figures.

According to the final estimate, a total of approximately **17,213** hip or knee joint implantations as defined by OPS codes 5,820 and 5,822 respectively (which explicitly do not include procedures in the sense of revisions) are to be expected at the partner clinics of PROMoting Quality within 12 months.

[1] Federal evaluation of knee arthroplasty care: Basic evaluation, 2017 IQTiG
[2] Federal evaluation: hip arthroplasty care: Baseline evaluation 2017, IQTiG

In addition, estimates suggest that about 17% of patients with implantations of a hip or knee joint cannot participate in the study due to exclusion criteria and about 15% of patients refuse to participate in the study for personal reasons. In addition, it is assumed that, despite intensive efforts, no PROMs can be collected from 30% of the study participants during the final survey.

Based on these estimates and taking into account the necessary case number adjustment for the reasons mentioned above, it is expected that **7,597 patients** will be enrolled and complete the study within the recruitment period.

On the one hand, this figure is likely to be reduced by a certain proportion of patients without direct or indirect Internet access; on the other hand, it can be expected that in the meantime the partner clinics will tend to perform even more hip and knee joint implantations than in 2015.

Taking **7,597 study participants as a** basis, it can be assumed on the basis of the numbers of insured persons or population shares of the health insurance funds participating in the study that routine data and thus health-related expenditures for a total of an estimated **1,403 study participants** can be taken into account within the framework of PROMoting Quality.

Table 1: Expected patient numbers, study participation, and cost data availability.

Description of the population	Total group	Patient-level randomization	
		Intervention Group	Control group
Total number of implantations in partner hospitals according to inclusion criteria (1.)	17.213	-	-
of which treatment cases with study participation (2.)	9.494	4.747	4.747
of which treatment cases with PROM survey (3.)	7.597	3.798	3.798
of which insured with partner insurers and insofar with availability also of cost data (4.)	1.403	701	701
Minimum required population size for statistical validation of group differences with respect to the primary outcome only (5.).	1.396	698	698

1.) Assumption for data collection period based on internal case numbers 2017 (inclusion of elective first implantation only; exclusion of fractures and age < 18 years).
2.) Assumption: 2% with other reasons for exclusion (e.g. patients with error rate on admission such as revision) and not already considered under 1.), 15% cases with refusal to participate in the study.
3.) Assumption: response rate 70%.
4.) Assumption: insured population share 19.35% (query by aQua Institute at BARMER, share of BKKs unchanged).
5.) Approximate assumption: intervention-related change in primary outcome corresponding to 0.15 times the standard deviation, statistical validation with an error probability of p = 5% with a probability of 80% (power) - see also following section on case number planning.

Study participants without cost data

During the conceptual design of PROMoting Quality, it was considered (especially against the background of the decision to consider the "outcome over cost ratio" as the primary outcome) whether it might make sense to make study participation a priori dependent on insurance with a health insurer involved in the study. The consideration was rejected primarily for the reasons listed below:

- The insurance-dependent restriction of study participation to about a quarter of the currently expected number of cases would no longer have allowed a meaningful implementation and everyday work of study nurses, especially in hospitals with lower case numbers. A realistic practical test of the work of corresponding
  Study Nurses in different clinics, however, represents an essential element in the overall project.

- Only if patients are included in the primary survey across all health insurance funds can any existing bias effects due to the restriction to insured persons of certain health insurance funds with regard to certain aspects be detected or excluded with a high degree of probability.

- The outcomes recorded with the primary survey form - also independently of a use in the combined primary study outcome (the "outcome over cost ratio") - very important parameters for the assessment of the study success, with which an availability to the largest possible population in each study is ensured.
  case is desirable. Moreover, only with data on comparatively large populations can subgroup analyses be performed, which may reveal different effects of the intervention in certain patient groups and thus lead to a more targeted use or optimization of the intervention.

of the intervention. Since the intervention is an innovative and novel procedure for which comparatively little practical experience is available to date, corresponding analyses not only appear desirable, but are, in the view of the consortium partners, a necessary component of PROMoting Quality.

Summary from the subsequent claims

In the course of the preparation period, the project executing agency made various additional requests, which were answered by the consortium management. In addition, this version answers questions from the last letter (A3), which are recorded under B3.
The course is listed systematically below:

A1 *Funding decision from 29.03.2019 (DLR)*
B1 *Unblocking request of 28.05.2019 (TUB).*
A2 *Amendment notice dated 05.08.2019 (DLR)*
B2 *Status Report/Requests as of 09/12/2019 (TUB).*
A3 *Legal basis_requirements as of 24.10.2019 (DLR).*
B3 Evaluation conceptV3_legal basis of 29.11.2019 (TUB).
A4 *Request for work plan amendment - further subsequent requests dated 16.09.2020 (DLR).*
B4 *Evaluation conceptV4 of 18.09.2020 (TUB/aQua)*

A1 A revised evaluation design shall be submitted that demonstrates the need for and added value of this Case number (9,817 patients) for secondary outcomes and subgroup analyses justified in more detail.

B1 According to the results of the updated calculation, the expected total number of study participants with successful PROM survey is now slightly higher at 9,833 patients instead of 9,817, despite the more conservative assumptions for inclusion and response rates. As already justified, both estimates are based on approximate values, which is why the value from the original application - to which the unblocking letter applies - should not be modified further.

In summary, we are seeking a funding commitment for the care delivery of 9,817 patients. To this end, we provide a detailed rationale below for the arguments in favor of collecting PROMs related to secondary outcomes.
In addition, for the sake of completeness, points are listed that influence the valid evaluation of the primary endpoint. This is to clarify the importance of the target total patient number for the evaluation of the primary endpoint as well as for the scientific investigation and evaluation of the secondary key endpoints.

B1Rationale in favor of total case count for secondary outcomes evaluation.
For the analysis of potential improvement in quality of life, optimization of functional status, minimization of pain and postoperative mortality, complication, readmission, and reoperation rates, and faster fitness for work through the use of PROMs (collectively: secondary outcomes), we aim to capture as complete a population as possible in the participating hospitals.

The secondary endpoints of the study are based on the *items defined in* the *ICHOM Standard Set (*see project proposal). They are highly relevant to the overall message of the study, as the resulting scores are equivalent to the "quality of outcome" examined for each patient. Only if complete data - including PROMs - are collected from all patients (i.e., **all** 9,817 cases), regardless of their insurance affiliation, is the statistical significance of the recorded quality of outcome, which is reflected in the secondary outcomes, ensured. This (in addition to cost-effectiveness) forms an essential basis for justifying the implementation of PROMs in standard care.

B2 Application admission of the Waldkliniken Eisenberg
In the clinic sample of the PROMoting Quality project, there was a change at short notice: Tabea Krankenhaus unfortunately decided against participating in the project during the preparation phase. Instead, the Waldkliniken Eisenberg GmbH could be won over quickly as a replacement, whose LOI is attached to this letter.

For the case number calculation, the dropout of patients potentially to be included in the study by Tabea Hospital was 850 participants (500 hip TEP and 350 knee TEP). For Waldkliniken Eisenberg, a higher number of patients of 1,637 can be expected (1012 hip TEP, 625 knee TEP). This leads to the fact that now in total for the calculation of the primary endpoint the data of more PROM patients can be included (number first 2,589, then 2,907, now 1,600). This has no impact on the total amount of health care services available (€823,332.90). We would like to emphasize at this point that the case number calculation is always only an assumption, and that it is contractually agreed with clinics that the remuneration to which they are entitled in each case cannot be increased even if there is more recruitment.

A total of € 823,332.90 is available for the health care services in the PROMoting Quality project. According to the case number calculation, a total sum of € 789,879 was recognized for the clinics, and an implementation lump sum of € 33,453.87 was subsequently approved.

According to the calculation, the Tabea Hospital would have been entitled to a maximum remuneration sum of € 44,710. We would like to request that the sum originally available to Tabea Hospital, as well as the budget resulting from slight shifts between the hospitals (incl. the implementation lump sum), be used for the remuneration of Waldkliniken Eisenberg.
The rounded remuneration shown below has been agreed with all nine participating hospitals, taking into account the respective costs of recruiting personnel and technical implementation. These remuneration amounts are currently being fixed in the cooperation agreement with the clinics.

**Clinical Center**	**Entitled remuneration amount**
HELIOS ENDO Clinic Hamburg	190.000 €
VAMED Baltic Sea Clinic Damp	60.000 €
Forest Clinics Eisenberg	80.000 €
Schön Klinik Neustadt	155.000 €
Schön Klinik Hamburg Eilbek	60.000 €
Charité - University Medicine Berlin	45.000 €
RoMed Clinic Prien a. Chiemsee	40.000 €
DIAKOVERE Annastift	60.000 €
Sana Clinics Sommerfeld	130.000 €
Total	***820.000 €***

A2The expected effect size for the primary endpoint must be named and justified.

B2 An intervention effect on the primary outcome in the sense of influencing a ratio of patient-reported outcomes and costs incurred by patients in the health care system cannot be validly estimated in advance due to a lack of comparable data from the literature.
With regard to its size, rather conservative assumptions should also be made against the background of the primarily comparatively limited intervention at patient level. The reason for this is that after certain operations have been performed on study participants as an intervention within a one-year follow-up period, only three more PROM surveys are scheduled than in the control group. Further actions may then only take place depending on (abnormal) results of the PROM survey.

The required conservative assumptions are taken into account within the case number calculations in the form of a lower expected effect size of the intervention. If there are two groups with the same group size, as in the current study design, the effect size (d) according to Cohen can be calculated from the mean values and the standard deviation. This is based on the pooled standard deviation. Cohen's d indicates the distance between two distributions in units of their common standard deviation. In the current study design, an intervention effect of 0.15 standard deviations is conservatively calculated, which corresponds to an effect size of this order in the case of a standardized mean.

Assuming this effect size of the intervention on the "outcome over cost ratio", the minimum number of study participants required was determined to be n = 1,396. Under the above assumptions, the number of n = 1,403 study participants with cost data expected in PROMoting Quality should be sufficient for statistical validation of study effects assumed here.

A2 Operationalization of the primary endpoint in the evaluation concept is mandatory and is to be

B2 The content basis for an outcome-over-cost ratio calculation in terms of a quotient are the outcome indicators defined in the ICHOM standard indicator set, whose index forms the numerator. These indicators are partly based on established instruments such as the EQ-5D-5L health status questionnaires, joint functional status questionnaires (HOOS-PS / KOOS-PS), pain assessment, but also clinical outcomes such as reoperations and inpatient 30, 60, or 90-day readmissions.
Since these outcome indicators are represented on different scales, whose distribution also differs, the outcomes may have to be standardized before they can be added up to an index. The exact composition of a final outcome index and its weightings will therefore only be determined in the course of the project and on the basis of actual data.
For the denominator, the available health-related and orthopedic intervention-related expenditures from the areas of outpatient and inpatient care, including expenditures for medications, as well as prescribed remedies and aids, are determined and summed. In addition, the costs of the intervention are to be taken into account. The quotient of numerator and denominator forms the outcome-over-cost ratio. The individual results of the patient-level indicators are standardized by z-scores and then combined into a merged index. This is a formative construct, as it is expected that the scores of the individual outcome indicators will correlate with each other. It is then planned to incorporate the standardized scores with an equal weighting of functionality, health status, clinical outcomes, and pain assessment into the merged outcome index. A similar procedure is favored in Shwartz et al.[3] to form merged indices.

B1Supporting rationale in favor of total case count for primary outcome evaluation.
For the evaluation of the primary endpoint, the 'outcome over cost ratio', the effect of PROMs on cost-effectiveness is to be investigated with the available data of all patients of the cooperating payers (BARMER, BKK-DV). As already mentioned in the original evaluation concept, the inclusion of additional health insurers would have significantly increased the effort of coordination, data preparation and evaluation, which is why the associated routine data can only be examined for a quarter of all patients in the project.
The transferability of the results to the overall population (so-called translation) is supported by a complete recording of the outcome side. Possible simulations or conclusions of cost data for German patients with statutory health insurance become possible on the basis of almost 30% of the patients included in the study. In order to obtain a realistic picture of the effort and the process in the clinics in parallel, the proportionality of effort and benefit should also be ensured for the technical installation - independent of the successful inclusion of the patients.
In order to avoid sample bias due to health insurance affiliation with BARMER or the BKKs, it is also necessary for valid results in the primary outcome to encourage all patients - who meet the other inclusion criteria - to participate in the study, regardless of their health insurance affiliation. Ultimately, a fundamental aspect of full case ascertainment is also practicability in everyday clinical practice and a balanced cost/benefit ratio.
To the disadvantage of practicability in the clinics and an organization with as little effort as possible by the study nurses, the selective approach of patients could lead to confusion (also on the patient side) and unnecessarily increase the assessment effort for the study nurses. In addition, the strategic coordination within the clinics on the part of the participating physicians and the clinical management requires effort each time - which was not priced in this project and thus speaks for a high interest on the part of the clinics - which should be positively opposed to the ultimate added value and significance of the study.

An essential part of the project is to test the expected effects not only with the entire study group, but also to consider isolated effects in subgroups, in order to ultimately be able to make a comprehensive statement about the effectiveness of the intervention. The aim is to clarify whether different effects can be observed depending on certain characteristics, such as age, gender, level of education, as well as clinical factors or care characteristics.

The analysis of subgroups naturally requires smaller case numbers, since a subset of the overall study group is considered in each case. Based on our own preliminary analyses on the basis of Germany-wide hospital accounting data of the DRG statistics, it can be assumed, for example, that approx. 14% of the

[3] See Shwartz et al. (2015). Composite measures of health care provider performance: a description of approaches. Milbank Q.

included in the study had an age of 80 years and older. In a subgroup analysis based on patients of advanced age, just under 700 patients would therefore remain in each of the intervention and control groups. This corresponds approximately to the minimum number of cases required to detect a possible difference between intervention and control group in the order of 1.5 times the standard deviation (probability of error p = 5%, power 80%).
For other subgroup characteristics, such as educational level or perioperative treatment approach (rapid recovery vs. conventional care), no information is currently available on the distribution in the overall study group. However, in view of the planned total number of patients, there are justified hopes that at least some of the planned subgroup analyses will also provide statistically relevant results here.

The following subgroup analyses are planned, in each of which the measured effects, e.g. with regard to quality of life and other clinical outcomes, will be compared:

- Age groups
- Gender
- Education level
- Rapid Recovery vs. Conventional Care
- Surgical procedure and type of implant
- Follow-up treatment in an inpatient vs. outpatient rehab facility

The aim of the subgroup analyses is to uncover any different effects of the intervention in specific patient groups in order to obtain indications for a targeted use or possible need for optimization of the intervention with regard to specific patient groups.
In addition, hospital characteristics (minimum length of stay, degree of specialization, teaching status) can also be contrasted with the quality of outcomes measured by patients by further stratification. This also enables a comparison with the clinical quality reported by hospitals (e.g. esQS).

In addition, some of the secondary outcomes studied, such as the occurrence of complications, can be assumed to have a low event rate. It is reported from the QSR process that surgical complications occur in approximately 3% of patients undergoing elective hip or knee replacement over a one-year period. With the planned total number of cases of 3,798 in each intervention and control group, a difference of the order of approximately one percentage point could be demonstrated with an error probability of p = 5% and a power of 80%.

As a last point, we would like to mention that the PROMoting Quality project is funded in the research focus "New forms of care". The difference to the research focus of "health care research" is mainly that no secondary data analysis a posteriori shall be performed, but primary data shall be collected. For the evaluation of the secondary endpoints and for a simulation of the effort and process of collecting PROMs for standard care, the statistical quantity must be sufficient as a reference in order to be able to make a final statement about whether there is potential for the new form of care.

A2 For the planned caseload of 2,589 participants with routine data delivery, the added value is to represent.

B2 It is expected that outcomes and costs will not change to the same degree in all areas of care, but that these will occur in different areas depending on the subgroup. The research hypothesis is that the PROM survey and early detection and addressing of aberrations may avoid major revision surgery and expensive hospitalizations in the first year, but may incur additional costs for conservative measures (e.g., physical therapy), for example. In order to reliably identify these cost differences and to assess whether these cost differences have a relevant influence on the total costs, it is necessary that the individual subgroups have sufficiently large case numbers.

A3 The available opinions regarding the number of cases are still insufficient. The added value for the cases exceeding the number of 2,589 participants with routine data delivery is continue to be presented in more detail.
Subgroups are to be defined for the primary research question and examples of the planned subgroup analyses are to be presented.

B3 The increase in the number of cases of participants with routine data delivery from 2,589 to 2,818 results from purely practical reasons. Due to the replacement of Tabea Krankenhaus by Waldkliniken Eisenberg, there is a slight increase in the number of treatment cases with PROM surveys from 9,817 to 10,243. Furthermore, the historical proportion of Barmer patients receiving knee or hip TEP at Waldkliniken is above the proportion of Barmer patients at Tabea Krankenhaus.
Two aspects are essential to mention: (1) the increase in the number of cases will be achieved with the means established in the unblocking, and (2) in contrast to other clinical trials, the patient will receive an additional benefit with the participation in the project "PROMoting Quality", which is not accompanied by any risks.

No subgroup analyses in the strict sense are planned for the primary research question. The primary research question aims to analyze the cost-effectiveness of follow-up and eventual intervention for the entire study population in order to assess its utility for standard care of all knee and hip TEP patients. However, based on routine or cost data, expenditures in intervention participants and controls in different major service areas should also be considered separately, as it is likely that the intervention does not affect all costs equally. For example, early intervention in the postoperative course is expected to tend to result in higher outpatient medical care expenditures in the intervention group than in the control group, at least within an initial period, although these (outpatient) expenditures should at the same time help to reduce costs for inpatient treatment after discharge from initial inpatient treatment with the target intervention. Correspondingly differentiated results would support the evaluation of the study success.

A2 Please check if for the planned subgroup analysis "OP type" a secondary research question is can be defined.

B2 Subgroup analyses corresponding to the overarching question of "OP type" can be performed on the basis of various data,. Thus, the following indicators are collected and merged by the study assistance:

- Procedure code
  (OPS codes endoprosthesis hip joint 5-820.0, 5-820.2, 5-820.3, 5-820.4, 5-820.5, 5-820.7, 5.820.8, 5.820.9, 5.820.x OPS codes endoprosthesis knee joint 5-822.0, 5-822.8, 5-822.9, 5-822.c, 5-822.f, 5.822.g, 5.822.j, 5.822.k, 5-822.x, 5-822.y)
- Pending: REF/LOT of the implant. A list has already been requested from the Endoprosthesis Register, but has not yet been made available.
- Experience of the main surgeon (number of procedures per year)

Conceivable research questions that arise from this include:

- Does the material type of the prosthesis (noncemented, cemented, hybrid) have an impact on the reoperation rate and readmissions in specific patient subgroups?
- Does the material type of the prosthesis (noncemented, cemented, hybrid) have an influence on the course of recovery, especially on functionality and general quality of life?

- Do certain implants used show higher quality, especially in functionality and general quality of life of the patient?
- How much does the experience of the surgeon affect quality? What are possibly sensible minimum values?

A2 Please check whether with the targeted number of cases further subgroup analyses / research to the degree of care, multimorbidity or migration status are possible.

B2 Thanks to the expected heterogeneous study population (the clinics of different sponsors participating in the study cover seven federal states), the analysis of the targeted number of cases of approx. 10,000 patients will most likely allow many further subgroup analyses or research questions, which will be concretized in the course of the evaluation preparation.

We would therefore like to thank you for the information on researching the degree of care. We have checked this and included it in the questionnaires (patients are asked whether they are assigned to one of the degrees of care from 1-5).

Conceivable research questions that arise from this include:

- Do patients who have a higher level of care experience more critical threshold crossings during the post-operative year?
- Is there a correlation between level of care and reoperation rate, and readmission?

We would also like to thank you for the information on researching comorbidity. At present, we already ask for the main diagnosis (ICD code) as well as the secondary diagnoses via the study assistance and check an allocation according to the Charlson Comorbidity Index or the Elixhauser Comorbidity Score. We ask the patient about comorbidities such as heart disease, high blood pressure, stroke, lung disease, diabetes mellitus or kidney disease.
Conceivable research questions that arise from this include:

- Do patients who have higher comorbidity experience more critical threshold crossings during the post-operative year?
- Is there a correlation between comorbidities and reoperation rate, as well as readmission? Which measures (physiotherapy, inpatient rehabilitation) have been shown to reduce the risk of reoperation in patients with more severe comorbidities?

We will not investigate research questions on migration status because we do not have valid information on nationality or migration background.

A3 It should be examined whether the inclusion of the criterion "migration background" in the context of the

B3 In the context of the letter of additional requirements dated 12.09.2019, it was already explained that the patient's migration background can unfortunately no longer be integrated into the patient surveys already in progress. Therefore, a subgroup analysis based on migration status will not be possible in the context of addressing our secondary research questions. Health insurance companies typically ask for nationality when members are admitted, which could be used as a proxy for the characteristic migration status in the context of analyses of (exclusively) routine data. However, this characteristic is not included in the already accessible BARMER data. Within the framework of the study, it will be clarified or estimated together with the participating health insurance funds which proportions of study participants with a non-German nationality are to be expected and then, on this basis, it will be decided whether and how evaluations on this topic are possible on the basis of routine data from the health insurance funds.

Caseload planning

In general, case number planning serves to estimate the required size of study populations in order to be able to statistically substantiate specific and a priori expected effects or subgroup differences within these study populations with a sufficiently high probability. Ideally, an adequate sample size planning should prove that the targeted study population size, which can be realized within a reasonable period of time, is sufficient to be able to statistically prove at least the expected effects.

At the same time, case number planning can also be used in certain studies to avoid unnecessarily large surveys or study populations. This point is particularly relevant when

a. if the prerequisites for precise case number planning are met (e.g., if a priori precise knowledge of the empirical distribution of the primary outcome in the intended target population exists and, at the same time, certain outcome differences can be considered clinically relevant without doubt),

b. if the planned intervention can at least potentially also have significant negative effects on patients and its use in unnecessarily large populations is in this respect ethically unjustifiable (e.g. administration of a previously unproven drug substance),

c. if a study is exclusively or at least predominantly concerned with a primary outcome, which is also taken into account in the planning of the number of cases, and other aspects play no or only a very minor role (as, for example, in the clinical trial of a new lipid-lowering agent in comparison with a standard therapy, which may initially only wish to demonstrate a relevant superiority with regard to lipid lowering and can at best record frequent side effects).

From the point of view of the project participants and the methodologist responsible for the evaluation, none of the conditions mentioned under a) to c) can be considered fulfilled in the context of PROMoting Quality. This applies already in view of the content-related study conception and the planned intervention clearly and without doubt in particular to the conditions listed under
b) and c) above.

Against this background, the case number planning presented in the PROMoting Quality application served exclusively and solely to estimate whether the number of study participants planned in the project was likely to be sufficient with a sufficiently high probability (power 80%) for the statistical validation of an expected intervention effect with regard to the primary outcome with the usual probability of error of p = 5%.

Assuming an intervention effect of the order of 0.15 times the standard deviation of the
"Outcome over Cost Ratio", the minimum number of study participants required was determined to be n = 1,396. Under these assumptions, the number of n = 1,600 study participants with cost data expected in PROMoting Quality should be sufficient for statistical validation of the study effects assumed here. It should be noted that the calculations described here could not be based on pre-existing information that would allow a precise estimate of the number of cases according to the example described in a).

Rationale for cross-payer patient engagement.
Comparatively large discrepancies between the results of the case number planning and the study population targeted in PROMoting Quality (by a factor of about 7), which are explicitly mentioned by the project sponsor, result from the intention not to limit study participation and collection of primary data to insured persons of the partner insurers (BARMER and insurers in the BKK umbrella organization).

The justification and necessity for this procedure results from different aspects and objectives of PROMoting Quality, which in part also appear to be extremely relevant independently of effects on the defined primary outcome and in this respect are largely independent of the described case number planning. The main justifications have already been described in the preceding text (cf. in particular the section under "Study participants without cost data") and will be listed again here.

- Within the framework of PROMoting Quality, a realistic practical test of the work of study nurses in different clinics is to be carried out. This would not make sense if study participation were limited to only about a quarter of the currently expected number of cases, especially in clinics with lower case numbers. possible.

- By including patients across health insurers in the primary survey, possible bias effects in the (limited) consideration of the primary outcome can be detected or excluded with a probability bordering on certainty.

- The outcomes recorded with the primary survey form very important parameters for the differentiated assessment of the study success (see section Primary collection of data) with regard to different aspects. For the overall assessment, it seems essential to be able to distinguish between effects of the intervention in different areas that are likely to be different.
- Likewise, subgroup analyses and multivariate analyses appear to be essential for revealing differentially pronounced effects of the intervention in specific patient groups and, more generally and independently of the intervention, also on risk factors for complications in the postoperative course, which may contribute to a more targeted use or optimization of the intervention in the future. Since the intervention is an innovative and novel procedure, corresponding steps are a necessary component of PROMoting Quality from the point of view of those involved in the study.

This should make it clear that the survey of PROMs and the care services provided with them are also included in the scientific monitoring and evaluation.

A1 In this context, the accessibility of case numbers should also be specified and, in particular, the to give more detailed reasons for the assumed high willingness to

B1 Accessibility of the case numbers

In the PROMoting Quality application, the case numbers in the partner hospitals were calculated based on the operation figures of the OPS codes 5,820 and 5,822 from 2015. In order to make these figures more precise and to calculate the associated personnel expenditure by the study nurses in the respective clinics as realistically as possible, we have asked the clinics for an internal estimate of the expected case numbers for the survey period from October 2019 to September 2020. The information should be based on the most recently measured number of cases (usually from 2017), taking into account the exclusion criteria we have specified, and should also take into account factors such as changes in physicians, mergers of wards, etc.

Although we are aware that the planned case numbers are approximations, we hope that by providing the internal figures we can give as true a picture as possible of the effort to be expected. The participant and response rates we assume will only be calculated subsequently on the basis of these figures.

An effort-based remuneration and the scale of the disbursement of funds defined with it will be submitted to you with the first status report as requested. These are additionally intended to ensure that the clinics have sufficient incentive to include the cases advised by the project.

Willingness to participate

Regarding the assumption of high participation rates, we highlight the difference between expected inclusion rates and response rates. Our assumptions are based on Heartbeat's experience in PROM capture in routine clinical practice across the company's customer base, which - similar to our study setting - covers a range of different levels of care.
Even though the reference studies do not involve explicit participation in a clinical trial, we also assume comparable values thanks to the use of low-threshold software.

- **Inclusion rate**: Heartbeat Medical's digital solution is easy to use and the questionnaires are collected on the tablets in the clinics without too much explanation or effort. The inclusion rate is on average between 80% - 90%. It is calculated by dividing the number of patients who agree to be surveyed by the total number of patients who would be eligible for the survey. Ultimately, we calculate an inclusion rate of 85% (instead of 90% previously) when calculating the number of cases.
  We requested explicit real-world examples of inclusion rates from Heartbeat Medical. The company bases its assumption on the clinics' published case numbers for the calculation. As an example, we would like to refer to a study in arthroplasty in which Heartbeat had the internal total number of cases as the basis for calculating the inclusion rate. This was 90%.
- **Response rate:** The response rate for Heartbeat is on average between 60% - 80% and results from the number of participating patients who have completed all questionnaires 1 year after treatment as a proportion of all patients who had originally consented to the survey. Ultimately, we calculate the number of cases with a response rate of 70% (90% in the project application).
  Heartbeat has also provided us with figures from previous practice for response rates. These are available for different homes and are as follows:

	House A	House B	House C	House D	House E
**Department (intervention)**	Orthopedics (knee/hip)	Orthopedics (knee/hip/foot)	Orthopedics (knee)	Endoprosthetics (knee/hip)	Sports orthopedics (knee/shoulder)
**Cases**	337	88	10.149	250	1.492
**Measured response rate**	85%	83%	85%	67%	60%

In support of this, we point out that the project uses an already existing and technically proven solution (Heartbeat's software has already been used in more than 80 thousand patients) and the estimation can be made on the basis of empirical values and with higher certainty. In addition, we believe that the willingness to participate can be positively influenced by the following other factors:

- **Study Nurse Training:** We intend to provide all responsible study nurses with comprehensible information materials about the project and the target population before the first patients are enrolled in October. This is to ensure that they can quickly decide in the clinic which patients are eligible for participation and also, if possible, only accept those patients who meet the inclusion criteria of the study. To this end, we are currently preparing information and are already planning telephone interviews with one or two study nurses, whom we will ask for their impression and an assessment of the plausibility of the materials.
- **Patient information: It** is assumed that patients will also be encouraged to participate by receiving comprehensible information about the project. For this purpose, the study nurse receives project brochures that she can hand out to the patients (an integral part here is, for example, an easy-to-understand presentation and explanation of the data protection concept). The documents on participation and informed consent should also be prepared in a way that is understandable for all patients and should not exceed two pages. In order to coordinate closely with the health insurance companies involved in the project, a kick-off meeting was held on April 8, 19. The topic of patient information was discussed in detail and it is planned to promote understanding of the PROMs concept and the project through articles in the respective member magazines (digital and/or analog).
- **Inquiries of the study nurse with the patient: It** is important to mention that the response rate can be influenced by the study nurse asking the patients and, from Heartbeat's experience, can also be significantly increased.

A4 Please provide a statement from the responsible evaluator indicating which Impact of the requested caseload reduction on the expected knowledge gain, and submit an updated evaluation concept taking into account the new case numbers. Please indicate the changes in the evaluation concept. In addition, it must be examined and explained whether the reduction in the number of cases results in a reduced workload for the evaluation.

B4 Gain in knowledge with reduced number of cases

With the projected case number of 9,494 patients with PROM survey of whom approximately 80% (approximately 7,597 patients) continue study participation to completion, and the resulting approximately 1,403 VBHC patients, an evaluation of the primary outcome is still possible as the required minimum case number of 1,396 patients will be met.

The reduction in PROM patients of approximately 26%, (7,597 versus 10,243 patients estimated to date) is expected to have some impact on the depth of subgroup analyses, however, it is anticipated that with the effect size of 0.15 standard deviations, analogous to the primary endpoint, subgroups with at least 18.4% proportion of the total number of cases can still be studied. Because the effects of the endpoints are only estimated due to the lack of comparable studies, it is not possible to determine the structure and depth of the subgroup analysis until the evaluation.

In the first example mentioned under B1 for the subgroup analysis of patients over 80 years of age, the change in the number of cases (531 instead of 700 patients) would lead to a slightly higher effect size than previously assumed (0.17 instead of 0.15) with the same marginal conditions for error probability (5%) and power (80%).

In the second example under B1, the secondary outcome of the occurrence of complications, which is possibly to be investigated, a difference of the order of approximately one percentage point (1.19% instead of 1.07%) can still be demonstrated with a prevalence of the outcome of 4%, with the expected total number of cases of 3,798 in each of the intervention and control groups.

Evaluation workload

A reduction in workload due to the reduction in the number of cases is not to be expected, as it is not the number of patients included in the study that is decisive for the evaluation, but rather the evaluation plan, which remains unchanged in terms of structure. A possible reduction of individual subgroup analyses is offset by the higher effort to merge the partial data export at the end of 2021, which has become necessary due to the shortened evaluation period, with the second partial data export in Q3 of 2022.

Responsible methodologist for the evaluation concept:
Thomas G. Grobe, MD, MPH (aQua Institute, Göttin gen)

Göttingen, 24.09.2020 — Thomas Grobe

Place, date — Signature

24. September 2020

PROMoting Quality - Intersektorale Nutzung von Patient Reported Outcome Measures zur Steigerung der patientenrelevanten Ergebnisqualität

Förderkennzeichen 01NVF18016

Evaluationskonzept V4

Zuständiger Methodiker und Ansprechpartner für das Evaluationskonzept:
Dr. med. Thomas G. Grobe, MPH

aQua – Institut für angewandte Qualitätsförderung
und Forschung im Gesundheitswesen GmbH
Maschmühlenweg 8–10 · 37073 Göttingen
Telefon (+49) 0551-789 52 - 139
thomas.grobe@aqua-institut.de

Hintergrund

Das vorliegende Dokument beinhaltet eine Zusammenstellung unterschiedlicher Fassungen des Evaluationskonzeptes. Berücksichtigt wurden Inhalte der ersten Fassung des Evaluationskonzepts (Version 1, 08. Januar 2019), der überarbeiteten Fassung (Version 2, 28. Mai 2019), der dritten Fassung (Version 3, 27. November 2019), welche in der vorliegenden vierten Fassung (Version 4, 18. September 2020) noch weiter ergänzt wurden.

Zur Vereinfachung der Lesbarkeit sind in dieser Version ausschließlich die final angestrebten bzw. erwarteten Fallzahlen aufgeführt (Ausnahmen gibt es dort, wo es rein um die Finanzierungszusage aus dem Entsperrungsantrag der ursprünglich beantragen Fallzahl ging). Veränderungen der jetzt genannten Fallzahlen im Vergleich zu Angaben in vorausgehenden Fassungen des Evaluationskonzeptes erklären sich mit Veränderungen hinsichtlich der teilnehmenden Kliniken (so hat z.B. das Tabea Krankenhaus seine Teilnahme zurückgezogen, was jedoch durch das Hinzustoßen der RoMed Kliniken und des Waldklinikums Eisenberg kompensiert wurde) sowie durch Rekrutierungsverluste, die mit der COVID-19 Pandemie einhergingen.

Da die Fallzahlabschätzungen im Rahmen des Projektes auf bestimmten Annahmen sowie einer möglichst nachvollziehbaren Methodik beruhen, führen entsprechende Veränderungen von Rahmenbedingungen zwangsläufig auch zu Veränderungen der rechnerisch erwarteten Fallzahlen. Aus Sicht der Studienbeteiligten muss dabei in erster Linie sichergestellt sein, dass die ursprünglich angestrebten Fallzahlen durch veränderte Rahmenbedingungen nicht unterschritten werden und insofern die Studie gefährden können. Bereits an dieser Stelle sei ausdrücklich darauf hingewiesen, dass höhere Fallzahlen die Aussagemöglichkeiten der Studie eher verbessern. Da die höheren Fallzahlen im Rahmen der Studie kostenneutral realisiert werden und negativen Effekte der Studienintervention auf die Gesundheit von Patienten mit sehr hoher Wahrscheinlichkeit ausgeschlossen werden können, sind mit einer Erhöhung der Fallzahlen keine Nachteile zu erwarten.

Bestandteile Version 1

In der Benachrichtigung des Innovationsausschusses beim G-BA mit Datum vom 19.10.2018 wurde als eine erste Auflage zur Durchführung des Projektes angeführt: *Es ist ein ausführlicheres Evaluationskonzept vorzulegen, das insbesondere die Methodik, die Interventions- und Kontrollgruppe sowie die Fallzahlplanung detaillierter darstellt. Die Vorgehensweise zur Vermeidung eines Bias ist zu erläutern. Die Fallzahl für die gesundheitlichen Versorgungsleistungen muss sich aus dem Evaluationskonzept herleiten lassen.*

Im Rahmen eines Telefonates zwischen den Antragstellern und dem Projektträger am 04.12.2018 wurden diese Aussagen konkretisiert. Im Protokoll zum Telefonat werden unter „2. Evaluationskonzept“ seitens des Projektträgers folgende drei Unterpunkte aufgeführt:

- *Es ist ein ausführlicheres Evaluationskonzept mit Unterschrift des zuständigen Methodikers vorzulegen, dass insbesondere die Methodik, die Interventions- und Kontrollgruppe sowie die Fallzahlplanung detaillierter darstellt.*
- *Es ist zu bedenken, dass durch den E-Mail-Kontakt ein Selektionsbias entstehen kann. Die Vorgehensweise zur Vermeidung eines Bias ist bitte zu erläutern.*
- *Die Fallzahl für die gesundheitlichen Versorgungsleistungen muss sich aus dem Evaluationskonzept herleiten lassen. Die Fallzahl für die gesundheitlichen Versorgungsleistungen umfasst allerdings deutlich mehr Personen (4.908), als durch die Evaluationskriterien (698) notwendig wären (Faktor 7). Dieser Widerspruch ist bitte aufzulösen.*

Die nachfolgenden Ausführungen stellen das Evaluationskonzept des Projektes PROMoting Quality – mit Rückgriff auf Textpassagen aus dem Original-Antrag – ausführlicher dar, wobei sowohl auf Strategien zur Vermeidung von Bias eingegangen wird als insbesondere auch nachvollziehbare Begründungen zu den angestrebten Fallzahlen geliefert werden sollen.

Mit den Ausführungen soll zugleich verdeutlicht werden, dass *„erbrachten Versorgungsleistungen auch Eingang in die wissenschaftliche Begleitung und Auswertung finden“*, was im Protokoll des Telefonates im Abschnitt unter *„Gesundheitliche Versorgungsleistungen“* als Voraussetzung für die Förderung entsprechender Leistungen genannt wird.

Bestandteile Version 2 und 3

Für die Erläuterungen haben wir uns an die Reihenfolge der Version 1 des Konzepts gehalten und die zusätzlichen Fragestellungen – da, wo inhaltlich passend – unter den jeweiligen Überschriften aus dem Förderbescheid (vom 29.03.19), dem Änderungsbescheid (vom 05.08.19) sowie den Nachforderungen (vom 24.10.19) in *grau hinterlegt* singemäß als Ausgangspunkt für die Beantwortung genutzt.

Bestandteile Version 4

Die aktuelle Version enthält sowohl neue Annahmen zu den zu erwartenden Fallzahlen aufgrund der geänderten COVID-19 Rahmenbedingungen als auch Erläuterungen zu deren Auswirkungen auf die geplanten Auswertungen. Weiterhin wird ein geänderter Evaluationsplan erläutert, der sich aus dem verlängerten Rekrutierungszeitraum ergibt.
Die aktualisierten Angaben sind in Schriftfarbe grün gekennzeichnet.

Versorgungsziele, Intervention und Zielpopulation

Die Intervention im Rahmen von PROMoting Quality hat – allgemein formuliert – zum Ziel, die gesundheitliche Entwicklung und Lebensqualität bei Patienten im zeitlichen Verlauf nach größeren medizinischen Interventionen/Eingriffen positiv zu beeinflussen. Der Gewinn an Gesundheit und Lebensqualität soll dabei kosteneffektiv, d.h. ohne einen überproportionalen Anstieg der aufgewendeten Ressourcen, realisiert werden, was mittelfristig zu Einsparungen bei der Inanspruchnahme von Gesundheitsleistungen führen kann. Neben der Ermittlung von Effekten der Intervention sollen im Rahmen des Projektes zudem auch die Praktikabilität des Vorgehens bei der Intervention nachgewiesen werden, Ansätze für eine Optimierung des Vorgehens gewonnen werden sowie die Übertragbarkeit der Ergebnisse und Auswirkungen einer Ausweitung der Intervention auf alle Patienten mit den betrachteten Zieleingriffen in Deutschland abgeschätzt werden.

Die **Intervention** besteht zunächst in einem regelmäßigen Monitoring von Patienten im zeitlichen Verlauf nach größeren medizinischen Interventionen/Eingriffen unter zur Hilfenahme von Patient Reported Outcome Measures (PROMs). Dadurch sollen Komplikationen und ungünstige Entwicklungen frühzeitig erkannt und entsprechend effektiver behandelt werden können. Bei ungünstigen Ereignissen und Entwicklungen erfolgt dann ggf. schwellenwertbezogen durch eine Study Nurse die Benachrichtigung des behandelnden Facharztes und darauf basierend eine engere und individuellere Betreuung im Sinne einer situationsgerechten Anpassung der Nachbehandlung.

Die **Zielpopulation** für eine entsprechende Intervention im Rahmen von PROMoting Quality bilden Patienten mit einem elektiven Hüft- oder Kniegelenksersatz. Grundsätzlich ließe sich die Intervention auch bei anderen medizinischen Interventionen/Eingriffen durchführen. Maßgeblich für eine Beschränkung auf genau diese Patientengruppe ist die Erwartung, dass innerhalb eines begrenzten Zeitraums unter Einbeziehung einer noch überschaubaren Anzahl an Zentren ausreichend viele Patienten mit vergleichsweise homogenen Charakteristika rekrutiert werden können. Nur unter diesen Bedingungen kann innerhalb eines begrenzten Studienzeitraums mit begrenzten Ressourcen die praktische Umsetzung der geplanten Intervention und deren Evaluation gewährleistet werden.

Evaluationsrelevante Outcomes

Bei der Evaluation von Interventionseffekten werden im Rahmen von PROMoting Quality unterschiedliche Merkmale und abgeleitete Kenngrößen berücksichtigt, welche auf unterschiedlichen Wegen erfasst werden.

Als **primäres Outcome** wird gemäß Projektantrag die „Outcome over Cost ratio“ betrachtet.

Als **sekundäre Outcomes** werden bewertet:

- Funktioneller Status (bewertet via HOOS-PS / KOOS-PS Score),
- Lebensqualität (bewertet via EQ-5D-3L Score),
- Schmerzen in Hüfte, Knie oder dem unteren Rücken,
- Arbeitsstatus,
- generelle Zufriedenheit mit Behandlungserfolg,
- Klinische Ergebnisindikatoren wie postoperative Wiederaufnahmerate, Reoperationsrate.

Die „Outcome over Cost ratio“ bildet das maßgebliche Ziel der Intervention – den kosteneffektiven Gewinn an Gesundheit und Lebensqualität ohne einen überproportionalen Anstieg der aufgewendeten Ressourcen – in einer einzelnen Kennzahl ab und wurde vor diesem Hintergrund als primäres Outcome festgelegt.

Gleichwohl kommt jedoch auch den sekundären Outcomes und dabei insbesondere auch der Einbeziehung der Patientenperspektive im Rahmen von PROMoting Quality bei der Beurteilung des Interventionserfolges eine wesentliche Rolle zu. Alle sekundären Outcomes sollten durch die Intervention in einem positiven Sinne beeinflusst werden, wobei im Rahmen der Evaluation zumindest negative Effekte mit hoher Wahrscheinlichkeit ausgeschlossen werden sollten.

Studiendesign (RCT) und Studienablauf

PROMoting Quality ist eine multizentrisch durchgeführte, randomisierte und kontrollierte Interventionsstudie (RCT) im klassischen Sinne. Dabei wird die randomisierte Zuordnung von Patienten zum Kontroll- oder Interventionsarm der Studie softwaregesteuert und unbeeinflusst durch die behandelnden Personen auf der Ebene der einzelnen Patienten erfolgen, womit aus allen Partnerkliniken (s.u.) gleichermaßen sowohl Patienten mit Zuordnung zur Kontroll- als auch zur Interventionsgruppe rekrutiert werden.

Eine Besonderheit des Forschungsdesigns besteht darin, dass zu Subgruppen der Patienten sowohl aus der Interventions- als auch aus der Kontrollgruppe im Falle einer Versicherung bei den projektbeteiligten Krankenkassen BARMER sowie des BKK-Dachverbands im Sinne eines 'Value Based Health Care' (VBHC) Ansatzes neben der Ergebnisqualität auch die Leistungs- und Abrechnungsdaten ausgewertet werden. Der Anteil dieser Patienten, deren Daten vornehmlich in die Bewertung des primären Endpunkts einfließen an der untersuchten Gesamtpopulation beträgt voraussichtlich knapp ein Drittel (29,06%). Gleichwohl finden alle erhobenen Daten, also sowohl Daten der 'VBHC-Patienten' als auch Daten der reinen PROM-Patienten (ohne die Verfügbarkeit von Leistungs- und Abrechnungsdaten) Eingang in die wissenschaftliche Begleitung und Auswertung.

Ein- und Ausschlusskriterien

Patienten mit Zieleingriffen sollen im Rahmen der Studie innerhalb eines 12-monatigen Zeitraums vollständig rekrutiert werden. Die rekrutierten Patienten mit einer Zuordnung zur Kontroll- oder Interventionsgruppe werden dann jeweils über einen Zeitraum von 12 Monaten ab dem Operationstag/Zieleingriff nachbeobachtet. Als Partnerkliniken sind bei PROMoting Quality insgesamt neun Kliniken mit unterschiedlichen Standorten in Hamburg, Schleswig-Holstein, Niedersachsen, Bayern, Berlin und Brandenburg beteiligt.

- **Einschlusskriterium** für die Studienteilnahme ist die elektive Durchführung eines erstmaligen Gelenkersatzes an Hüfte oder Knie in einer der Partnerkliniken innerhalb des Rekrutierungszeitraums (Eingriffe mit den Operationen- und Prozedurenschlüsseln (OPS) 5-820 „Implantation einer Endoprothese am Hüftgelenk“ sowie 5-822 „Implantation einer Endoprothese am Kniegelenk“.
- **Von einer Studienteilnahme ausgeschlossen** werden Patienten, die bei Krankenhausaufnahme jünger als 18 Jahre alt sind, welche bereits vorausgehend einen Gelenkersatz am selben Gelenk erhalten haben, welche notfallmäßig (z.B. nach hüftgelenksnahe Femurfraktur) operiert werden sowie Patienten der ASA-Klassen 4 bis 6 (Patient mit lebensbedrohlicher Erkrankung; moribunder Patient, der ohne Operation wahrscheinlich nicht überleben wird; gestorbener Patient mit festgestelltem Hirntod, Organspender).

  Zudem werden Patienten von einer Studienteilnahme ausgeschlossen, welche absehbar über die Studiendauer weder mittelbar noch unmittelbar über E-Mail erreichbar sind und welche keinen Zugang zum Internet haben.

Randomisierung

Die randomisierte Zuordnung von geeigneten Patienten (gemäß Einschlusskriterium und ohne eines der genannten Ausschlusskriterien) zur Interventions- oder Kontrollgruppe erfolgt nach einer Einwilligung zur Studienteilnahme im Rahmen der Baseline-PROM-Erhebung, die im Studienablauf für beide Gruppen obligat erforderlich ist. Die Zuordnung wird dabei so implementiert, dass sie ohne jegliche Einflussnahme durch den behandelnden Arzt oder den Patienten abläuft. Patienten werden dabei in allen Partnerkliniken mit gleichen Wahrscheinlichkeiten jeweils zufällig entweder der Interventions- oder der Kontrollgruppe zugeordnet, womit pro Klinik sowie auch insgesamt annähernd die gleiche Zahl an Interventions- und Kontrollgruppenpatienten resultieren sollte.

Mit dem beschriebenen Vorgehen ist aus Sicht der Studienbeteiligten sichergestellt, dass Studienteilnehmer im besten Sinne eines RCTs – und abgesehen von zufallsbedingten Abweichungen – ohne jegliches Bias der Interventions- oder Kontrollgruppe zugeordnet werden. Die im Rahmen der Baseline-PROM-Erhebung erstmalig der Interventions- oder der Kontrollgruppe zugeordneten Patienten bilden den Ausgangspunkt aller nachfolgenden Analysen, wobei sich Auswertungen unter Einbeziehung genau dieser Patienten, welche zeitlich vor einer ggf. stattfindenden Intervention selektiert wurden, auch als Intention-to-treat-Analysen bezeichnen lassen. Bezogen auf die Subgruppe von Versicherten der BARMER sowie von Krankenkassen mit Zugehörigkeit zum BKK Dachverband kann im Rahmen von PROMoting Quality zugleich mit der späteren Verfügbarkeit nicht verzerrter Daten bis zum Ende des geplanten Beobachtungszeitraum ein Jahr nach dem operativen Gelenkersatz auch unabhängig von der weiteren Beteiligung der Patienten an der PROM-Erhebung gerechnet werden.

Primärerhebung von Daten (insbesondere PROMs)

Bei allen Patienten mit Einschlusskriterium und ohne Ausschlussgründe erfolgt bei der stationären Aufnahme und nach einer schriftlichen Einwilligung in die Studienteilnahme mittels der Software Heartbeat ONE eine Baseline-PROM-Erhebung, die damit also noch vor dem operativen Eingriff in der Partnerklinik erfolgt.

Ausschließlich in der Interventionsgruppe erfolgen softwaregestützt vier weitere PROM-Erhebungen, nämlich einen Tag vor Entlassung sowie regulär auch in den Monaten 1, 3 und 6 nach dem operativen Eingriff.

Eine erneute und abschließende PROM-Erhebung bei allen in die Studie eingeschlossenen Patienten ist 12 Monate nach Durchführung des Zieleingriffes vorgesehen. Es wird angenommen, dass nach entsprechendem Einsatz der Study Nurses Ergebnisse abschließender PROM-Erhebung zu rund 70% der Studienteilnehmer erfasst werden können.

Die Verwaltung der PROM-Ergebnisse durch Study Nurses umfasst neben einer manuellen Nachverfolgung fehlender PROM-Daten auch die Schulung der Patienten mit dem PROM-Erhebungstool Heartbeat ONE bei stationärer Aufnahme und vor Entlassung (nur für Interventionsgruppe I), das Komplementieren des ICHOM Standard Sets um die gelenkspezifische Krankheitsgeschichte (zurückliegende Indikationen und (chirurgische) Prozeduren) und um klinische Ergebnisqualitätsindikatoren, die Dokumentation von Variablen aus dem OP-Bericht (Teamzusammensetzung, Erfahrung des Operateurs und Implantatwahl) und den §21 KHEntgG-Daten (stationäre Verweildauer, Entlassungsart/-grund) sowie die initiale Sichtung der PROM-Ergebnisse, Weiterleitung kritischer PROM-Ergebnisse an relevante Einrichtungen (in jedem Fall an behandelnden Facharzt).

Zur Unterstützung der PROM-Analyse werden auffällige PROM-Ergebnisse durch die Software Heartbeat ONE prioritär angezeigt. Auffällige PROM-Ergebnisse sind hierbei als Überschreitung definierter kritischer Werte und/oder einer kontinuierlichen Werteverschlechterung definiert. Basierend auf den weitergeleiteten auffälligen PROM-Ergebnissen kann der behandelnde Facharzt (zusätzliche) Untersuchungen bzw. Interventionen anordnen, um den identifizierten Symptomen und Beschwerden abzuhelfen.

Sekundärnutzung von Daten bei Krankenkassen (insbesondere Kosten)
Insbesondere zur Ermittlung von Kosten der gesundheitlichen Versorgung bei Studienteilnehmern, aber auch zur Abschätzung der Übertragbarkeit von Studienergebnissen auf die bundesweite Versorgung, werden Daten genutzt, die routinemäßig bei Krankenkassen erfasst werden. Für eine Bereitstellung entsprechender Daten konnten im Rahmen von PROMoting Quality als Kooperationspartner die BARMER sowie der BKK Dachverband gewonnen werden. Abschätzungen zur Übertragbarkeit von Studienergebnissen erfolgen vorrangig auf der Basis von Daten der BARMER. Bei der BARMER und dem BKK Dachverband angehörenden Kassen dürften schätzungsweise insgesamt etwa rund 30% der bundesdeutschen Bevölkerung versichert sein, womit voraussichtlich Routinedaten zu etwas mehr als einem Viertel aller Studienteilnehmer im Rahmen von PROMoting Quality genutzt werden können.

Weitere Krankenkassen konnten im Vorfeld der Studienplanung nicht für eine Beteiligung an dem Projekt gewonnen werden. Die Einbeziehung weiterer Krankenkassen hätte in jedem Fall die kassenseitig anfallenden Aufwände erhöht. Zugleich wäre der Koordinierungsaufwand sowie der Auffand bei der Datenaufbereitung im Zuge der Evaluation merklich gestiegen, da unterschiedliche Datawarehouse-Lösungen bei den Krankenkassen eingesetzt werden.

Erwartete Patientenzahlen und Studienteilnahme
Alle Kliniken wurden darum gebeten, ihre Fallzahlen entsprechend der Ausschlusskriterien (Patienten unter 18 Jahre, Frakturen sowie Patienten der ASA-Klassen 4-6) zu konkretisieren. Dies ermöglicht eine Einbeziehung nur der Patienten mit gewünschten Einschlusskriterien in die Evaluation.

Die erwarteten Patientenzahlen mussten allerdings im Laufe der Vorbereitungszeit korrigiert werden.

- Gründe dafür waren Veränderungen im Kliniksample: Zwei Kliniken (Asklepios Westklinikum Hamburg und das Tabea Krankenhaus) haben ihre Teilnahme abgesagt, dafür sind zwei neue Kliniken hinzugestoßen (RoMed Klinik Prien a. Chiemsee und die Waldkliniken Eisenberg).
- Zusätzlich mussten die erwarteten Patientenzahlen aufgrund der besonderen Situation des COVID-19 Lockdown und der damit verbundenen Aussetzung geplanter- und der Terminierung neuer Hüft- und Knie-Endoprothesenoperationen erneut angepasst werden. Die Krankenhäuser waren aufgrund der Vorhaltung von Ressourcen im Bereich der intensivmedizinischen Betten für an COVID-19 erkrankten Patienten, sowie der Risikominimierung hinsichtlich der Ansteckung besonders gefährdeter Altersgruppen (das Durchschnittsalter der Untersuchungspopulation liegt bei > 68 Jahre[1,2]) nicht in der Lage, diese in der Regel elektiven Operationen zeitgerecht durchzuführen. Dies führte zu einem erheblichen Einbruch der Rekrutierungszahlen.

Der finalen Schätzung zufolge ist bei den Partnerkliniken von PROMoting Quality innerhalb von 12 Monaten mit insgesamt etwa **17.213** Implantationen eines Hüft- oder Kniegelenkes im Sinne der OPS-Codes 5.820 bzw. 5.822 zu rechnen (welche Eingriffe im Sinne von Revisionen explizit nicht einschließen).

[1] Bundesauswertung Knieendoprothesenversorgung: Basisauswertung, 2017 IQTiG
[2] Bundesauswertung: Hüftendoprothesenversorgung: Basisauswertung 2017, IQTiG

Zusätzlich lassen Abschätzungen vermuten, dass rund 17% der Patienten mit Implantationen eines Hüft- oder Kniegelenkes aufgrund von Ausschlusskriterien nicht an der Studie teilnehmen können und rund 15% der Patienten eine Studienteilnahme aus persönlichen Gründen ablehnen. Zudem wird davon ausgegangen, dass, ungeachtet intensiver Bemühungen, bei 30% der Studienteilnehmer keine PROMs im Rahmen der abschließenden Befragung erhoben werden können.

Basierend auf diesen Abschätzungen und unter Berücksichtigung der notwendigen Fallzahlanpassung aus oben genannten Gründen wird damit gerechnet, dass innerhalb des Rekrutierungszeitraumes **7.597 Patienten** in die Studie eingeschlossen werden können und diese auch beenden.

Diese Zahl dürfte sich einerseits noch um einen gewissen Anteil von Patienten ohne eine unmittelbare oder mittelbare Internetzugangsmöglichkeit reduzieren, andererseits ist damit zu rechnen, dass in den Partnerkliniken zwischenzeitlich tendenziell noch mehr Implantationen von Hüft- und Kniegelenken als 2015 vorgenommen werden.

Bei Zugrundelegung von **7.597 Studienteilnehmern** kann aufgrund der Versichertenzahlen bzw. Bevölkerungs-anteilen der studienbeteiligten Krankenkassen davon ausgegangen werden, dass im Rahmen von PROMoting Quality Routinedaten und damit gesundheitsbezogene Ausgaben zu insgesamt schätzungsweise **1.403 Studienteilnehmern** berücksichtigt werden können.

Tabelle 1: Erwarte Patientenzahlen, Studienteilnahme und Kostendatenverfügbarkeit

Beschreibung der Population	Gesamtgruppe	Randomisierung auf Patientenebene	
		Interventionsgruppe	Kontrollgruppe
Gesamtzahl der Implantationen in Partnerkliniken gemäß Einschlusskriterien (1.)	17.213	-	-
davon Behandlungsfälle mit Studienteilnahme (2.)	9.494	4.747	4.747
davon Behandlungsfälle mit PROM-Erhebung (3.)	7.597	3.798	3.798
davon versichert bei Partnerkassen und insofern mit Verfügbarkeit auch von Kostendaten (4.)	1.403	701	701
Minimal erforderliche Populationsgröße zur statistischen Absicherung von Gruppenunterschieden ausschließlich hinsichtlich des primären Outcomes (5.)	1.396	698	698

1.): Annahme für Erhebungszeitraum basierend auf internen Fallzahlen 2017 (Einschluss nur elektive Erstimplantation; Ausschluss von Frakturen und Alter < 18 Jahre)
2.) Annahme: 2% mit anderweitigen (z.B. Patienten mit Fehlerquote bei Aufnahme wie etwa Revision) und nicht bereits unter 1.) berücksichtigten Ausschlussgründen, 15% Fälle mit Verweigerung der Studienteilnahme.
3.) Annahme: Rücklaufquote 70%.
4.) Annahme: Versicherter Bevölkerungsanteil 19,35% (Abfrage durch aQua Institut bei BARMER, Anteil BKKen gleichbleibend).
5.) Approximative Annahme: Interventionsbedingte Veränderung des primären Outcomes entsprechend dem 0,15-fachen der Standardabweichung, statistische Absicherung mit einer Fehlerwahrscheinlichkeit von p = 5% mit einer Wahrscheinlichkeit von 80% (Power) – vgl. auch nachfolgenden Abschnitt zur Fallzahlplanung.

Studienteilnehmer ohne Kostendaten

Im Rahmen der Konzeptionierung von PROMoting Quality wurde abgewogen (insbesondere auch vor dem Hintergrund der Entscheidung, die „Outcome over Cost ratio" als primäres Outcome zu betrachten), ob es sinnvoll sein könnte, die Studienteilnahme a priori von der Versicherung bei einer studienbeteiligten Krankenkasse abhängig zu machen. Die Überlegung wurde vorrangig aus den nachfolgend aufgeführten Gründen verworfen:

- Die versicherungsabhängige Beschränkung von Studienteilnahmen auf etwa ein Viertel der jetzt erwarteten Fallzahlen hätte insbesondere in Kliniken mit geringeren Fallzahlen keine sinnvolle Implementierung und alltagsnahe Arbeit von Study Nurses mehr erlaubt. Ein realitätsnaher Praxistest der Arbeit entsprechender Study Nurses in unterschiedlichen Kliniken stellt jedoch ein wesentliches Element im Gesamtprojekt dar.
- Nur mit einer kassenübergreifenden Einbeziehung von Patienten bei der Primärerhebung lassen sich ggf. vorhandene Verzerrungseffekte durch die Beschränkung auf Versicherte bestimmter Krankenkassen hinsichtlich bestimmter Aspekte aufdecken bzw. mit hoher Wahrscheinlichkeit ausschließen.
- Die mit der Primärerhebung erfassten Outcomes bilden – auch unabhängig von einer Nutzung im kombinierten primären Studien-Outcome (der „Outcome over Cost ratio") sehr wesentliche Kenngrößen zur Beurteilung des Studienerfolgs, womit eine Verfügbarkeit zu einer möglichst großen Population in jedem Fall wünschenswert ist. Nur mit Daten zu vergleichsweise großen Populationen lassen sich zudem auch Subgruppenanalysen durchführen, die ggf. unterschiedlich ausgeprägte Effekte der Intervention bei bestimmten Patientengruppen aufdecken und damit zu einem gezielteren Einsatz oder einer Optimierung

der Intervention beitragen können. Da es sich bei der Intervention um ein innovatives und neuartiges Vorgehen handelt, zu dem bislang vergleichsweise wenige Praxiserfahrungen verfügbar sind, erscheinen entsprechende Analysen nicht nur wünschenswert, sondern sind aus Sicht der Konsortialpartner notwendiger Bestandteil von PROMoting Quality.

Zusammenfassung aus den Nachforderungen

Im Zuge der Vorbereitungszeit wurden durch den Projektträger verschiedene Nachforderungen gestellt, die durch die Konsortialführung beantwortet wurden. Zudem werden in dieser Version Nachfragen aus dem letzten Schreiben (A3) beantwortet, diese sind unter B3 festgehalten.
Der Verlauf wird im Folgenden systematisch aufgeführt:

A1 *Förderbescheid vom 29.03.2019 (DLR)*
B1 *Entsperrungsantrag vom 28.05.2019 (TUB)*
A2 *Änderungsbescheid vom 05.08.2019 (DLR)*
B2 *Statusbericht/Nachforderungen vom 12.09.2019 (TUB)*
A3 *Rechtsgrundlage_Nachforderungen vom 24.10.2019 (DLR)*
B3 EvaluationskonzeptV3_Rechtsgrundlage vom 29.11.2019 (TUB)
A4 *Antrag auf Arbeitsplanänderung - weitere Nachforderungen vom 16.09.2020 (DLR)*
B4 *EvaluationskonzeptV4 vom 18.09.2020 (TUB/aQua)*

A1 Es ist ein überarbeitetes Evaluationskonzept vorzulegen, das die Notwendigkeit und den Mehrwert dieser Fallzahl (9.817 Patienten) für die sekundären Outcomes und Subgruppenanalysen ausführlicher begründet.

B1 Nach Ergebnissen der aktualisierten Kalkulation liegt die erwartete Gesamtzahl an Studienteilnehmern mit erfolgreicher PROM-Erhebung trotz der konservativeren Annahmen bei Einschluss- und Rücklaufquote statt 9.817 nun geringfügig höher bei 9.833 Patienten. Wie bereits begründet beruhen beide Schätzungen auf Näherungswerten, weshalb der Wert aus dem ursprünglichen Antrag – für den das Entsperrungsanschreiben gilt - nicht weiter modifiziert werden soll.

Zusammengefasst streben wir eine Finanzierungszusage für die Versorgungsleistung von 9.817 Patienten an. Dafür wird im Folgenden ausführlich begründet, welche Argumente für die Erhebung von PROMs in Bezug auf die Sekundäroutcomes sprechen.
Zusätzlich werden der Vollständigkeit halber Punkte aufgeführt, welche die valide Evaluation des Primärendpunkts beeinflussen. So soll verdeutlicht werden, welche Bedeutung die angestrebte Gesamtpatientenzahl sowohl für die Evaluation des Primärendpunktes als auch für die wissenschaftliche Untersuchung und Evaluation zu den sekundären Schlüsselendpunkten hat.

B1 Begründung zugunsten der Gesamtfallzahlerhebung für die Evaluation der Sekundäroutcomes
Für die Analyse einer potenziellen Verbesserung der Lebensqualität, einer Optimierung des funktionellen Status, der Minimierung von Schmerzen und postoperativen Mortalitäts-, Komplikations-, Wiederaufnahme- und Re-Operationsraten sowie der schnelleren Arbeitsbefähigung durch den Einsatz von PROMs (insgesamt: Sekundäroutcomes) streben wir die Erfassung einer möglichst vollständigen Population in den teilnehmenden Kliniken an.

Die sekundären Endpunkte der Studie basieren auf den im *ICHOM Standard Set definierten Items (*siehe dazu den Projektantrag). Sie sind von hoher Relevanz für die Gesamtaussage der Studie, da die daraus resultierenden Scores gleichzusetzen sind mit der untersuchten „Ergebnisqualität“ eines jeden Patienten. Nur wenn von allen Patienten vollständige Daten – auch PROMs erhoben werden (d.h. **alle** 9.817 Fälle), und zwar unabhängig von ihrer Versicherungszugehörigkeit, ist die statistische Aussagekraft der erfassten Ergebnisqualität, welche sich in den Sekundäroutcomes widerspiegelt, sichergestellt. Diese bildet (neben der Kosteneffektivität) eine wesentliche Grundlage für die Begründung einer Umsetzung von PROMs in der Regelversorgung.

B2 Antrag Aufnahme der Waldkliniken Eisenberg
Im Klinik-Sample des PROMoting Quality Projektes kam es kurzfristig zu einer Veränderung: Das Tabea Krankenhaus hat sich leider im Verlauf der Vorbereitung gegen eine Projektteilnahme entschieden. Dafür konnte als Ersatz zügig die Waldkliniken Eisenberg GmbH gewonnen werden, deren LOI diesem Schreiben beigefügt ist.

Für die Fallzahlkalkulation betrug der Ausfall der durch das Tabea Krankenhaus potenziell in die Studie einzuschließenden Patienten 850 Teilnehmer (500 Hüft-TEP und 350 Knie-TEP). Bei den Waldkliniken Eisenberg ist mit einer höheren Patientenanzahl von 1.637 zu rechnen (1012 Hüft-TEP, 625 Knie-TEP). Dies führt dazu, dass nun insgesamt für die Berechnung des primären Endpunktes die Daten von mehr PROM-Patienten einfließen können (Anzahl zuerst 2.589, dann 2.907, nun 1.600). Auf die gesamte, zur Verfügung stehende Summe der Gesundheitlichen Versorgungsleistungen (823.332,90 €) hat dies keinen Einfluss. Wir möchten an dieser Stelle betonen, dass es sich bei der Fallzahlkalkulation immer nur um eine Annahme handelt, und dass mit Kliniken vertraglich geregelt ist, dass die ihnen jeweils zustehende Vergütung auch bei einer Mehrrekrutierung nicht erhöht werden kann.

Insgesamt stehen für die Gesundheitlichen Versorgungsleistungen im PROMoting Quality Projekt 823.332,90 € zur Verfügung. Gemäß Fallzahlkalkulation wurde eine Gesamtsumme von 789.879 € für die Kliniken anerkannt, sowie nachträglich eine Implementierungspauschale von 33.453,87 € bewilligt.

Laut Kalkulation hätte dem Tabea Krankenhaus eine maximale Vergütungssumme von 44.710 € zugestanden. Wir möchten gerne beantragen, die ursprünglich dem Tabea-Krankenhaus zur Verfügung stehende Summe, sowie das durch leichte Verschiebungen zwischen den Kliniken (inkl. der Implementierungspauschale) entstandene Budget für die Vergütung der Waldkliniken Eisenberg einzusetzen.
Mit allen neun teilnehmenden Kliniken ist – unter Berücksichtigung des jeweiligen Aufwands zur Personalgewinnung und zur technischen Implementierung – untenstehende gerundete Vergütung abgestimmt. Diese Vergütungssummen werden derzeit im Kooperationsvertrag mit den Kliniken fixiert.

**Klinikum**	**Zustehende Vergütungssumme**
HELIOS ENDO-Klinik Hamburg	190.000 €
VAMED Ostseeklinik Damp	60.000 €
Waldkliniken Eisenberg	80.000 €
Schön Klinik Neustadt	155.000 €
Schön Klinik Hamburg Eilbek	60.000 €
Charité - Universitätsmedizin Berlin	45.000 €
RoMed Klinik Prien a. Chiemsee	40.000 €
DIAKOVERE Annastift	60.000 €
Sana Kliniken Sommerfeld	130.000 €
Gesamt	***820.000 €***

A2 Die erwartete Effektstärke für den primären Endpunkt muss benannt und begründet werden.

B2 Ein Interventionseffekt auf das primäre Outcome im Sinne der Beeinflussung eines Quotienten aus den patientenseitig berichteten Outcomes und der durch die Patienten im Gesundheitssystem verursachten Kosten kann im Vorfeld, mangels vergleichbarer Daten aus der Literatur, nicht valide geschätzt werden.
Hinsichtlich seiner Größe sollten auch vor dem Hintergrund der primär vergleichsweise beschränkten Intervention auf Patienten-Ebene eher konservative Annahmen getroffen werden. Grund dafür ist, dass nach Durchführung bestimmter Operationen bei Studienteilnehmern als Intervention innerhalb eines einjährigen Nachbeobachtungszeitraums planmäßig lediglich drei PROM-Erhebungen mehr als in der Kontrollgruppe erfolgen. Weitere Aktionen erfolgen dann ggf. erst abhängig von (auffälligen) Ergebnissen der PROM-Erhebung.

Die geforderten konservativen Annahmen werden im Rahmen der Fallzahlberechnungen in Form einer geringeren erwarteten Effektstärke der Intervention berücksichtigt. Handelt es sich um zwei Gruppen mit gleicher Gruppengröße, wie im aktuellen Studiendesign, so kann aus den Mittelwerten sowie der Standardabweichung die Effektstärke (d) nach Cohen berechnet werden. Dabei wird die gepoolte Standardabweichung zugrunde gelegt. Cohens d gibt den Abstand zweier Verteilungen in Einheiten ihrer gemeinsamen Standardabweichung an. Im aktuellen Studiendesign wird konservativ mit einem Interventionseffekt von 0,15 Standardabweichungen gerechnet, was im Falle eines standardisierten Mittelwertes einer Effektstärke in dieser Größenordnung entspricht.

Unter Annahme dieser Effektstärke der Intervention auf die „Outcome over Cost Ratio“ wurde als minimal erforderliche Anzahl an Studienteilnehmern die Anzahl von n = 1.396 ermittelt. Unter den genannten Annahmen sollte die in PROMoting Quality erwartete Anzahl der n = 1.403 Studienteilnehmer mit Kostendaten ausreichend zur statistischen Absicherung von hier angenommenen Studieneffekten sein.

A2 Eine Operationalisierung des primären Endpunktes im Evaluationskonzept ist zwingend erforderlich und ist zu ergänzen.

B2 Inhaltliche Grundlage für eine Outcome-over-Cost-Ratio Berechnung im Sinne eines Quotienten sind die im ICHOM-Standard Indikatoren-Set festgelegten Ergebnisindikatoren, deren Index den Zähler bildet. Diese Indikatoren basieren z.T. auf etablierten Instrumenten wie beispielsweise den EQ-5D-5L Fragebögen zum Gesundheitszustand, Fragebögen zum funktionalen Status des Gelenks (HOOS-PS / KOOS-PS), zur Schmerzbewertung, aber auch zu klinischen Outcomes, wie Reoperationen und stationäre 30, 60 bzw. 90 Tage Wiederaufnahmen.
Da diese Ergebnisindikatoren sich auf unterschiedlichen Skalen abbilden, deren Verteilung sich zudem unterscheidet, müssen vor der Verrechnung zu einem Index ggf. Standardisierungen der Outcomes vorgenommen werden. Die genaue Zusammensetzung eines finalen Outcome-Index und seiner Gewichtungen wird somit erst im Laufe des Projektes und auf Basis tatsächlicher Daten ermittelt werden.
Für den Nenner werden die verfügbaren gesundheitsbezogenen und mit dem orthopädischen Eingriff im Zusammenhang stehenden Ausgaben aus den Bereichen der ambulanten und stationären Versorgung inklusive der Ausgaben für Medikamente, sowie der verordneten Heil- und Hilfsmittel ermittelt und summiert. Zudem sind die Kosten der Intervention zu berücksichtigen. Der Quotient aus Zähler und Nenner bildet die Outcome-over-Cost Ratio.
Die einzelnen Ergebnisse der Indikatoren auf Patientenebene werden per z-Scores standardisiert und dann zu einem zusammengeführten Index kombiniert. Es handelt sich hierbei um ein formatives Konstrukt, da erwartet wird, dass die Scores der einzelnen Ergebnisindikatoren untereinander korrelieren. Es ist dann geplant, die standardisierten Scores mit einer Gleichgewichtung von Funktionalität, Gesundheitszustand, klinischen Outcomes und Schmerzbewertung in den zusammengeführten Outcome-Index einfließen zu lassen. Ein ähnliches Verfahren wird in Shwartz et al.[3] zur Bildung von zusammengeführten Indizes favorisiert.

B1 Unterstützende Begründung zugunsten der Gesamtfallzahlerhebung für die Evaluation des Primäroutcomes
Für die Bewertung des primären Endpunkts, der 'Outcome over Cost ratio', sollen mit den zur Verfügung stehenden Daten aller Patienten der kooperierenden Kostenträger (BARMER, BKK-DV) die Wirkung von PROMs auf die Kosteneffektivität untersucht werden. Wie im ursprünglichen Evaluationskonzept bereits erwähnt, hätte die Einbeziehung weiterer Krankenkassen den Aufwand der Koordination, Datenaufbereitung und Evaluation deutlich erhöht, weshalb im Projekt nur bei einem Viertel aller Patienten die zugehörigen Routinedaten untersucht werden können.
Die Übertragbarkeit der Ergebnisse auf die Gesamtpopulation (sog. Translation) wird unterstützt durch eine vollständige Erfassung der Ergebnisseite. Mögliche Simulationen bzw. Schlussfolgerungen von Kostendaten für die gesetzlich Krankenversicherten Deutschlands werden auf Basis von fast 30% der in der Studie erfassten Patienten möglich. Um parallel ein realistisches Abbild des Aufwands und des Ablaufs in den Kliniken zu erhalten, sollte auch die Verhältnismäßigkeit von Aufwand und Nutzen bei der technischen Installation - unabhängig vom erfolgreichen Einschluss der Patienten - sichergestellt werden.
Damit es nicht zu einer Stichprobenverzerrung durch die Kassenzugehörigkeit zur BARMER bzw. den BKKen kommt, ist es auch für valide Ergebnisse im primären Outcome notwendig, alle Patienten - die den sonstigen Einschlusskriterien entsprechen - unabhängig von ihrer Krankenkassenzugehörigkeit zu einer Teilnahme an der Studie zu ermutigen. Ein elementarer Aspekt der vollen Fallzahlerhebung sind letztlich auch die Praktikabilität im Klinikalltag und ein ausgewogenes Kosten/Nutzen-Verhältnis.
Zu Ungunsten der Praktikabilität in den Kliniken und einer möglichst aufwandsarmen Organisation durch die Study Nurses, könnte die selektive Ansprache von Patienten zu Verwirrung führen (auch auf Patientenseite) und den Einschätzungsaufwand für die Study Nurses unnötig erhöhen. Zusätzlich fällt auch in der strategischen Koordination innerhalb der Kliniken auf Seiten der beteiligten Ärzte und der klinischen Leitung jedes Mal Aufwand an - der in diesem Projekt nicht eingepreist wurde und somit für ein hohes Interesse seitens der Kliniken spricht -, welcher der letztlichen Wertschöpfung und Aussagekraft der Studie positiv gegenüberstehen sollte.

Ein wesentlicher Bestandteil des Projektes ist es, die zu erwartenden Effekte nicht nur mit der Gesamtstudiengruppe zu testen, sondern auch isolierte Effekte in Subgruppen zu betrachten, um letztendlich eine umfängliche Aussage über die Effektivität der Intervention treffen zu können. Dabei soll geklärt werden, ob in Abhängigkeit von bestimmten Merkmalen, wie z.B. Alter, Geschlecht, Bildungsstand, sowie klinischen Faktoren oder Versorgungsmerkmalen, unterschiedlich ausgeprägte Effekte zu beobachten sind.

Die Analyse von Subgruppen bedingt naturgemäß kleinere Fallzahlen, da dabei jeweils eine Teilmenge der Gesamtstudiengruppe betrachtet wird. Basierend auf eigenen Voranalysen auf Grundlage der deutschlandweiten Krankenhausabrechnungsdaten der DRG-Statistik ist beispielsweise davon auszugehen, dass ca. 14% der

[3] Vgl. Shwartz et al. (2015). Composite measures of health care provider performance: a description of approaches. Milbank Q.

eingeschlossenen Patienten ein Alter von 80 Jahren und höher aufweisen. In einer Subgruppenanalyse bezogen auf Patienten im höheren Lebensalter würden in Interventions- und Kontrollgruppe daher jeweils knapp 700 Patienten verbleiben. Dies entspricht in etwa der Mindestfallzahl, die zur Erkennung eines möglichen Unterschiedes zwischen Interventions- und Kontrollgruppe in der Größenordnung des 1,5-fachen der Standardabweichung (Fehlerwahrscheinlichkeit p = 5%, Power 80%) erforderlich ist.
Zu anderen Subgruppenmerkmalen, wie z.B. dem Bildungsstand oder dem perioperativen Behandlungsansatz (Rapid Recovery vs. Conventional Care) liegen derzeit keine Informationen zur Verteilung in der Gesamtstudiengruppe vor. Angesichts der geplanten Gesamtfallzahl von Patienten bestehen jedoch auch hier berechtigte Hoffnungen, dass zumindest ein Teil der vorgesehenen Subgruppenanalysen auch hier statistisch relevante Ergebnisse liefern werden.

Geplant sind die folgenden Subgruppenanalysen, in denen jeweils die gemessenen Effekte z.B. hinsichtlich Lebensqualität und sonstigen klinischen Outcomes gegenübergestellt werden:

- Altersgruppen
- Geschlecht
- Bildungsstand
- Rapid Recovery vs. Conventional Care
- Operationsverfahren und Typ des Implantats
- Nachbehandlung in einer stationären vs. ambulanten Reha Einrichtung

Ziel der Subgruppenanalysen ist, ggf. unterschiedlich ausgeprägte Effekte der Intervention in bestimmten Patientengruppen aufzudecken, um damit Hinweise für einen gezielten Einsatz oder auch möglichen Optimierungsbedarf der Intervention in Bezug auf bestimmte Patientengruppen zu erhalten.
Darüber hinaus können durch weiteres Stratifizieren auch Krankenhauscharakteristika (Mindestverweildauer, Spezialisierungsgrad, Lehrstatus) der von den Patienten gemessenen Ergebnisqualität gegenübergestellt werden. Dies ermöglicht auch einen Vergleich mit der von den Kliniken berichteten klinischen Qualität (z.B. esQS).

Bei einigen der untersuchten Sekundäroutcomes, wie z.B. dem Auftreten von Komplikationen, ist zudem von einer geringen Ereignisrate auszugehen. Aus dem QSR-Verfahren wird berichtet, dass chirurgische Komplikationen im Zeitraum von einem Jahr bei etwa 3% der Patienten mit elektivem Hüft- oder Kniegelenkersatz auftreten. Mit der geplanten Gesamtfallzahl von jeweils 3.798 in Interventions- und Kontrollgruppe ließe sich mit einer Fehlerwahrscheinlichkeit von p = 5% und einer Power von 80% ein Unterschied in der Größenordnung von ca. einem Prozentpunkt nachweisen.

Als letzten Punkt möchten wir anführen, dass das PROMoting Quality Projekt im Forschungsschwerpunkt "Neue Versorgungsformen" gefördert wird. Der Unterschied zum Forschungsschwerpunkt der "Versorgungsforschung" liegt vornehmlich darin, dass keine Sekundärdatenanalyse a posteriori durchgeführt, sondern Primärdaten erhoben werden sollen. Für die Bewertung der sekundären Endpunkte und für eine Simulation von Aufwand und Ablauf der Erhebung von PROMs für die Regelversorgung, muss die statistische Größe als Referenz ausreichend sein, um abschließend eine Aussage darüber treffen zu können, ob es Potenzial für die neue Versorgungsform gibt.

A2 Für die geplante Fallzahl von 2.589 Teilnehmern mit Routinedatenlieferung ist der Mehrwert darzustellen.

B2 Es ist zu erwarten, dass die Outcomes und Kosten sich nicht in allen Versorgungsbereichen in gleichem Maße verändern, sondern diese sich je nach Subgruppe in unterschiedlichen Bereichen ergeben. Die Forschungshypothese ist, dass durch die PROM-Erhebung und frühzeitige Erkennung und Adressierung von Fehlentwicklungen möglicherweise größere Revisionseingriffe und teure Krankenhausaufenthalte im ersten Jahr vermieden werden können, aber dafür beispielsweise zusätzliche Kosten für konservative Maßnahmen (z.B. Physiotherapie) anfallen. Um diese Kostenunterschiede sicher zu erkennen und einschätzen zu können, ob diese Kostenunterschiede einen relevanten Einfluss auf die Gesamtkosten haben, ist es notwendig, dass die einzelnen Subgruppen hinreichend große Fallzahlen aufweisen.

A3 Die vorliegenden Stellungnahmen bzgl. der Fallzahl sind weiterhin nicht ausreichend. Der Mehrwert für die über die Fallzahl von 2.589 Teilnehmern mit Routinedatenlieferung hinausgehenden Fälle ist weiterhin ausführlicher darzustellen.
Für die primäre Forschungsfrage sind Subgruppen zu definieren und die geplanten Subgruppenanalysen beispielhaft darzustellen.

B3 Die Erhöhung der Fallzahl der Teilnehmer mit Routinedatenlieferung von 2.589 auf 2.818 ergibt sich aus rein praktischen Gründen. Aufgrund des Austauschs des Tabea Krankenhauses durch die Waldkliniken Eisenberg ergibt sich eine leicht erhöhte Fallzahl der Behandlungsfälle mit PROM-Erhebungen von 9.817 auf 10.243. Des Weiteren befindet sich der historische Anteil der Barmer-Patienten, welche in den Waldkliniken eine Knie- oder Hüft-TEP erhalten, über dem Anteil der Barmer-Patienten des Tabea Krankenhauses.
Es sind hierbei zwei Aspekte essentiell zu erwähnen: (1) Die Erhöhung der Fallzahl wird mit denen in der Entsperrung festgesetzten Mitteln bewerkstelligt, und (2) im Gegensatz zu anderen klinischen Studien, erhält der Patient mit der Teilnahme am Projekt „PROMoting Quality“ einen zusätzlichen Nutzen, dem keinerlei Risiken gegenüberstehen.

Für die primäre Forschungsfrage sind keine Subgruppenanalysen im engeren Sinne geplant. Die primäre Forschungsfrage zielt darauf hin, die Kosteneffektivität der Nachverfolgung und eventuellen Intervention für die gesamte Studienpopulation zu analysieren, um deren Nutzen für die Regelversorgung aller Knie- und Hüft-TEP Patienten zu bewerten. Allerdings sollen, basierend auf den Routine- bzw. Kostendaten, Ausgaben bei Interventionsteilnehmern und Kontrollen in unterschiedlichen Hauptleistungsbereichen auch separat betrachtet werden, da davon auszugehen ist, dass die Intervention nicht alle Kosten gleichermaßen beeinflusst. So ist beispielsweise zu erwarten, dass durch frühzeitige Interventionen im postoperativen Verlauf die Aufwendungen für die ambulante ärztliche Versorgung in der Interventionsgruppe, zumindest innerhalb eines initialen Zeitraums, eher höher als in der Kontrollgruppe liegen, wobei diese (ambulanten) Aufwendungen zugleich dazu beitragen sollten, Kosten für stationäre Behandlungen nach Entlassung aus der initialen stationären Behandlung mit dem Zieleingriff zu reduzieren. Entsprechend differenzierte Ergebnisse würden die Bewertung der Studienerfolge unterstützen.

A2 Bitte prüfen Sie, ob für die geplante Subgruppenanalyse „OP-Typ“ eine sekundäre Forschungsfrage definiert werden kann.

B2 Subgruppenanalysen, die der übergeordneten Frage nach dem „OP-Typ“ entsprechen, können auf Grundlage von verschiedenen Daten, durchgeführt werden. So werden folgende Indikatoren durch die Studienassistenz erfasst und zusammengeführt:

- Prozedurenschlüssel
  (OPS-Kodes Endoprothese Hüftgelenk 5-820.0, 5-820.2, 5-820.3, 5-820.4, 5-820.5, 5-820.7, 5.820.8, 5.820.9, 5.820.x
  OPS-Kodes Endoprothese Kniegelenk 5-822.0, 5-822.8, 5-822.9, 5-822.c, 5-822.f, 5.822.g, 5.822.j, 5.822.k, 5-822.x, 5-822.y)
- Ausstehend: REF/LOT des Implantats. Eine Liste ist beim Endoprothesenregister bereits angefragt, jedoch noch nicht zur Verfügung gestellt worden.
- Erfahrung des Hauptoperateurs (Anzahl der Eingriffe pro Jahr)

Denkbare Forschungsfragen, die sich hieraus ergeben, sind z.B.:

- Hat der Materialtyp der Prothese (nicht zementiert, zementiert, hybrid) einen Einfluss auf die Reoperationsrate und Wiederaufnahmen bei bestimmen Patientensubgruppen?
- Hat der Materialtyp der Prothese (nicht zementiert, zementiert, hybrid) einen Einfluss auf die Genesungsverläufe, insbesondere auf Funktionalität und generelle Lebensqualität?

- Zeigen gewisse eingesetzte Implantate eine höhere Qualität, insbesondere bei Funktionalität und genereller Lebensqualität des Patienten?
- Wie stark wirkt sich die Erfahrung des Operateurs auf die Qualität aus? Was sind möglicherweise sinnvolle Mindestwerte?

A2 Bitte prüfen Sie, ob mit der angestrebten Fallzahl weitere Subgruppenanalysen / Forschungsfragen zum Pflegegrad, zur Multimorbidität oder zum Migrationsstatus möglich sind.

B2 Die Analyse der angestrebten Fallzahl von ca. 10.000 Patienten erlaubt Dank einer zu erwartenden heterogenen Studienpopulation (die an der Studie teilnehmenden Kliniken verschiedenster Trägerschaft erstrecken sich auf sieben Bundesländer) mit hoher Wahrscheinlichkeit viele weitere Subgruppenanalysen bzw. Forschungsfragen, welche im Laufe der Evaluationsvorbereitung konkretisiert werden.

Somit danken wir für den Hinweis zur Erforschung des Pflegegrads. Diesen haben wir geprüft und in die Fragebögen mit aufgenommen (Patienten werden befragt, ob sie einem der Pflegegrade von 1-5 zugeordnet sind.).

Denkbare Forschungsfragen, die sich hieraus ergeben, sind z.B.:

- Kommt es bei Patienten, die einen höheren Pflegegrad haben, vermehrt zu kritischen Schwellenwertüberschreitungen während des post-operativen Jahres?
- Gibt es eine Korrelation zwischen Pflegegrad und Reoperationsrate, sowie Wiederaufnahme?

Wir danken ebenfalls für den Hinweis zur Erforschung der Komorbidität. Wir erfragen derzeit schon über die Studienassistenz die Hauptdiagnose (ICD-Code) sowie die Nebendiagnosen und prüfen eine Zuordnung nach dem Charlson Comorbidity Index bzw. dem Elixhauser Comorbidity Score. Wir erheben durch den Patienten Komorbiditäten wie eine Herzerkrankung, Bluthochdruck, Schlaganfall, Lungenerkrankung, Diabetes mellitus oder Nierenerkrankungen.
Denkbare Forschungsfragen, die sich hieraus ergeben, sind z.B.:

- Kommt es bei Patienten, welche eine höhere Komorbidität aufweisen, vermehrt zu kritischen Schwellenwertüberschreitungen während des post-operativen Jahres?
- Gibt es eine Korrelation zwischen Komorbiditäten und Reoperationsrate, sowie Wiederaufnahme? Welche Maßnahmen (Physiotherapie, stationäre Reha) reduzieren nachweisbar das Reoperationsrisiko bei Patienten mit stärkeren Komorbiditäten?

Forschungsfragen zum Migrationsstatus werden wir nicht untersuchen, da wir keine validen Informationen zur Nationalität bzw. des Migrationshintergrundes vorliegen haben.

A3 Es ist zu prüfen, ob die Aufnahme des Kriteriums „Migrationshintergrund“ im Rahmen der Fragebogenerhebung integriert werden kann.

B3 Im Rahmen des Schreibens der Nachforderungen vom 12.09.2019 wurde bereits dargelegt, dass der Migrationshintergrund des Patienten leider nicht mehr in die schon laufenden Befragungen der Patienten integriert werden kann. Daher wird eine Subgruppenanalyse anhand des Migrationsstatus im Rahmen der Bearbeitung unserer sekundären Forschungsfragen nicht möglich sein. Von den Krankenkassen wird typischerweise bei einer Aufnahme von Mitgliedern die Nationalität erfragt, welche als Proxy für das Merkmal Migrationsstatus im Rahmen der Auswertungen (ausschließlich) von Routinedaten herangezogen werden könnte. Allerdings ist dieses Merkmal in den bereits zugänglichen Daten der BARMER nicht enthalten. Im Rahmen der Studie wird gemeinsam mit den beteiligten Krankenkassen geklärt bzw. abgeschätzt, bei welchen Anteilen von Studienteilnehmern mit einer nicht-deutschen Nationalität zu rechnen ist und dann auf dieser Basis entschieden, ob und wie Auswertungen zu diesem Thema auf der Basis von Routinedaten der Krankenkassen möglich sind.

Fallzahlplanung

Die Fallzahlplanung dient ganz allgemein der Abschätzung der erforderlichen Größe von Studienpopulationen, um innerhalb dieser Studienpopulationen bestimmte und a priori erwartete Effekte bzw. Subgruppenunterschiede ggf. auch mit einer ausreichend hohen Wahrscheinlichkeit statistisch abgesichert belegen zu können. Eine adäquate Fallzahlplanung sollte im Idealfall also nachweisen, dass die angestrebte und zugleich in angemessener Zeit realisierbare Studienpopulationsgröße ausreichend ist, um zumindest die erwarteten Effekte statistisch belegen zu können.

Zugleich kann eine Fallzahlplanung bei bestimmten Studien auch dazu herangezogen werden, um unnötig große Erhebungen bzw. Studienpopulationen zu vermeiden. Dieser Punkt ist insbesondere dann relevant,

a. wenn die Voraussetzungen für eine präzise Fallzahlplanung erfüllt sind (z.B. wenn a priori genaue Kenntnisse zur empirischen Verteilung des primären Outcomes in der angestrebten Zielpopulation existieren und zugleich bestimmte Ergebnisdifferenzen zweifelsfrei als klinisch relevant erachtet werden können),

b. wenn die geplante Intervention zumindest potenziell auch erhebliche negative Auswirkungen auf Patienten haben kann und ihr Einsatz bei unnötig großen Populationen insofern ethisch nicht vertretbar ist (z.B. die Verabreichung einer zuvor nicht erprobten Arzneimittelsubstanz),

c. wenn es im Rahmen einer Studie ausschließlich oder zumindest weit überwiegend um ein auch bei der Fallzahlplanung berücksichtigtes primäres Outcome geht und andere Aspekte keine oder nur eine sehr untergeordnete Rolle spielen (wie z.B. bei der klinischen Erprobung eines neuen Lipidsenkers im Vergleich zu einer Standardtherapie, die ggf. zunächst nur eine relevante Überlegenheit hinsichtlich der Lipidsenkung aufzeigen möchte und dabei allenfalls häufige Nebenwirkungen erfassen kann).

Aus Sicht der Projektbeteiligten und des für die Evaluation zuständigen Methodikers kann im Rahmen von PROMoting Quality keine der unter a) bis c) genannten Bedingungen als erfüllt gelten. Dies gilt bereits in Anbetracht der inhaltlichen Studienkonzeption und der geplanten Intervention eindeutig und zweifelsfrei insbesondere für die unter b) und c) genannten Punkte.

Die im Rahmen der Antragstellung von PROMoting Quality dargestellte Fallzahlplanung diente vor diesem Hintergrund ausschließlich und einzig der Abschätzung, ob die im Rahmen des Projektes geplante Anzahl an Studienteilnehmern mit ausreichend hoher Wahrscheinlichkeit (Power 80%) für die statistische Absicherung eines erwarteten Interventionseffektes hinsichtlich des primären Outcomes mit der üblichen Irrtumswahrscheinlichkeit von p = 5% voraussichtlich ausreichend sein dürften.

Unter Annahme eines Interventionseffektes in einer Größenordnung des 0,15-fachen der Standardabweichung der „Outcome over Cost Ratio" wurde als minimal erforderliche Anzahl an Studienteilnehmern die Anzahl von n = 1.396 ermittelt. Unter den genannten Annahmen sollte, die in PROMoting Quality erwartete, Anzahl der n = 1.600 Studienteilnehmer mit Kostendaten ausreichend zur statistischen Absicherung von hier angenommenen Studieneffekten sein. Einschränkend muss darauf hingewiesen werden, dass die hier beschriebenen Berechnungen nicht auf vorbestehende Informationen zurückgreifen konnten, die eine präzise Fallzahlabschätzung entsprechend dem unter a) beschriebenen Beispiel erlauben.

Begründung für die kassenübergreifende Einbeziehung von Patienten
Vergleichsweise große und vom Projektträger explizit erwähnte Diskrepanzen zwischen den Ergebnissen der Fallzahlplanung und der im Rahmen von PROMoting Quality angestrebten Studienpopulation (um etwa den Faktor 7) resultieren aus der Absicht, die Studienteilnahme und Erhebung von Primärdaten nicht auf Versicherte der Partnerkassen (BARMER sowie Kassen im BKK Dachverband) zu beschränken.

Die Begründung und Notwendigkeit für dieses Vorgehen resultiert aus unterschiedlichen Aspekten und Zielen von PROMoting Quality, die zum Teil auch unabhängig von Effekten auf den festgelegten primären Outcome ausgesprochen relevant erscheinen und insofern weitgehend unabhängig von der beschriebenen Fallzahlplanung sind. Die wesentlichen Begründungen wurden bereits im vorausgehenden Text beschrieben (vgl. insbesondere den Abschnitt unter „Studienteilnehmer ohne Kostendaten") und sollen an dieser Stelle nochmals aufgeführt werden.

- Im Rahmen von PROMoting Quality soll ein realitätsnaher Praxistest der Arbeit von Study Nurses in unterschiedlichen Kliniken erfolgen. Dies wäre bei einer Beschränkung von Studienteilnahmen auf nur etwa ein Viertel der jetzt erwarteten Fallzahlen insbesondere in Kliniken mit geringeren Fallzahlen nicht sinnvoll möglich.

- Durch eine kassenübergreifende Einbeziehung von Patienten bei der Primärerhebung lassen sich mögliche Verzerrungseffekte bei der (eingeschränkten) Betrachtung des primären Outcomes aufdecken oder mit an Sicherheit grenzender Wahrscheinlichkeit ausschließen.

- Die mit der Primärerhebung erfassten Outcomes bilden sehr wesentliche Kenngrößen zur differenzierten Beurteilung des Studienerfolgs (siehe Abschnitt Primärerhebung von Daten) im Hinblick auf unterschiedliche Aspekte. Für die Gesamteinschätzung erscheint es wesentlich, zwischen voraussichtlich unterschiedlich deutlichen Auswirkungen der Intervention in unterschiedlichen Bereichen unterscheiden zu können.
- Gleichfalls wesentlich erscheinen Subgruppenanalysen und multivariate Analysen zur Aufdeckung unterschiedlich ausgeprägter Effekte der Intervention bei bestimmten Patientengruppen sowie ganz allgemein und unabhängig von der Intervention auch zu Risikofaktoren für Komplikationen im postoperativen Verlauf, die zukünftig zu einem gezielteren Einsatz oder einer Optimierung der Intervention beitragen können. Da es sich bei der Intervention um ein innovatives und neuartiges Vorgehen handelt, sind entsprechende Schritte aus Sicht der Studienbeteiligten notwendiger Bestandteil von PROMoting Quality.

Hiermit sollte deutlich werden, dass die Erhebung von PROMs und damit erbrachte Versorgungsleistungen auch Eingang in die wissenschaftliche Begleitung und Auswertung finden.

A1 In diesem Zusammenhang ist auch die Erreichbarkeit der Fallzahlen zu präzisieren und insbesondere die angenommene hohe Teilnahmebereitschaft ausführlicher zu begründen.

B1 Erreichbarkeit der Fallzahlen

Im Antrag von PROMoting Quality wurden die Fallzahlen in den Partnerkliniken basierend auf den Operationszahlen der OPS-Codes 5.820 bzw. 5.822 aus dem Jahr 2015 berechnet. Um diese Zahlen nun zu präzisieren und den damit einhergehenden Personalaufwand durch die Study Nurses in den jeweiligen Kliniken möglichst realistisch einzukalkulieren, haben wir die Kliniken um eine interne Einschätzung der zu erwartenden Fallzahlen für den Erhebungszeitraum von Oktober 2019 bis September 2020 gebeten. Die Angabe soll auf der zuletzt gemessenen Fallzahl (i.d.R. von 2017) unter Beachtung der von uns präzisierten Ausschlusskriterien basieren und auch Faktoren wie Arztwechsel, Stationszusammenlegungen o.Ä. berücksichtigen.

Obgleich uns bewusst ist, dass die geplanten Fallzahlen Näherungswerte sind, erhoffen wir uns durch die Angabe der internen Zahlen ein möglichst getreues Bild des zu erwartenden Aufwands zu machen. Die von uns angenommenen Teilnehmer- und Rücklaufquoten werden erst anschließend auf der Basis dieser Zahlen berechnet.

Eine aufwandsbezogene Vergütung und die damit festgelegte Staffelung der Mittelausschüttung werden Ihnen wie gefordert mit dem ersten Statusbericht vorgelegt. Diese sollen zusätzlich sicherstellen, dass die Kliniken genügend Anreiz dazu haben, die vom Projekt avisierten Fälle einzuschließen.

Teilnahmebereitschaft

Bezüglich der Annahme einer hohen Teilnahmebereitschaft wird der Unterschied zwischen den erwarteten Einschlussquoten und den Rücklaufquoten hervorgehoben. Unsere Annahmen basieren auf Erfahrungswerten von Heartbeat bei der PROM-Erfassung in der klinischen Routine beim gesamten Kundenstamm des Unternehmens, welcher – ähnlich wie in unserem Studiensetting – eine Bandbreite verschiedener Versorgungsstufen abdeckt.
Auch wenn es sich bei den Referenzstudien nicht um die explizite Teilnahme an einer klinischen Studie handelt, gehen wir auch dank der Nutzung einer niedrigschwellig bedienbaren Software von vergleichbaren Werten aus.

- **Einschlussquote**: Die digitale Lösung von Heartbeat Medical ist leicht zu bedienen und die Fragebögen werden ohne allzu großen Erklärungsbedarf bzw. ohne großen Aufwand auf den Tablets in den Kliniken erhoben. Die Einschlussquote liegt im Schnitt zwischen 80% - 90%. Sie ergibt sich aus der Anzahl der Patienten, die einer Befragung zustimmen geteilt durch die Anzahl der Patienten, die insgesamt für die Befragung in Frage kämen. Letztlich kalkulieren wir bei der Berechnung der Fallzahlen mit einer Einschlussquote von 85% (anstatt vorher 90%).
  Wir haben von Heartbeat Medical explizite Beispiele aus der Praxis zu den Einschlussquoten erfragt. Das Unternehmen stützt seine Annahme auf Basis der veröffentlichten Fallzahlen der Kliniken für die Berechnung. Als Beispiel möchten wir hier eine Studie in der Endoprothetik heranziehen, bei welcher Heartbeat die interne gesamte Fallzahl als Berechnungsgrundlage für die Einschlussquote vorlag. Diese lag bei 90%.
- **Rücklaufquote:** Die Rücklaufquote liegt bei Heartbeat im Schnitt zwischen 60% - 80% und ergibt sich aus der Anzahl an teilnehmenden Patienten, die 1 Jahr nach der Behandlung alle Fragebögen ausgefüllt haben als Anteil aller Patienten, die ursprünglich zur Befragung eingewilligt hatten. Letztlich kalkulieren wir bei der Berechnung der Fallzahlen mit einer Rücklaufquote von 70% (im Projektantrag 90%).
  Auch für die Rücklaufquoten hat uns Heartbeat Zahlen aus der bisherigen Praxis zur Verfügung gestellt. Diese stehen für unterschiedliche Häuser zur Verfügung und lauten folgendermaßen:

	Haus A	Haus B	Haus C	Haus D	Haus E
**Fachbereich (Eingriff)**	Orthopädie (Knie/Hüfte)	Orthopädie (Knie/Hüfte/Fuß)	Orthopädie (Knie)	Endoprothetik (Knie/Hüfte)	Sportorthopädie (Knie/Schulter)
**Fälle**	337	88	10.149	250	1.492
**Gemessene Rücklaufquote**	85%	83%	85%	67%	60%

Unterstützend weisen wir darauf hin, dass das Projekt eine bereits existierende und technisch erprobte Lösung nutzt (die Software von Heartbeat war bereits bei über 80 Tausend Patienten im Einsatz) und die Abschätzung auf Basis von Erfahrungswerten und mit höherer Sicherheit getätigt werden können. Zusätzlich kann die Teilnahmebereitschaft aus unserer Sicht von folgenden weiteren Faktoren positiv beeinflusst werden:

- **Schulung der Study Nurse:** Wir beabsichtigen, alle verantwortlichen Study Nurses noch vor Einschluss der ersten Patienten im Oktober mit verständlichen Informationsmaterialien über das Projekt und die Zielpopulation zu informieren. So soll sichergestellt werden, dass sie in der Klinik schnell entscheiden können, welche Patienten für eine Teilnahme infrage kommen und auch möglichst nur die Patienten aufnehmen, die den Einschlusskriterien der Studie entsprechen. Zu diesem Zweck bereiten wir aktuell Informationen auf und planen bereits Telefoninterviews mit ein bis zwei Study Nurses, die wir um ihren Eindruck und eine Beurteilung zur Plausibilität der Materialien bitten.
- **Patienteninformation:** Es wird davon ausgegangen, dass auch die Patienten durch verständliche Informationen über das Projekt zu einer Teilnahme ermutigt werden. Zu diesem Zweck erhält die Study Nurse Projektbroschüren, die sie den Patienten aushändigen kann (Bestandteil hier ist zum Beispiel eine leicht anschauliche Darstellung und Erklärung des Datenschutzkonzepts). Auch die Unterlagen zu Teilnahme- und Einwilligungserklärung sollen für alle Patienten verständlich aufbereitet und maximal zwei Seiten betragen. Um sich diesbezüglich auch mit den projektbeteiligten Krankenkassen eng abzustimmen, hat am 08.04.19 bereits ein Auftakttreffen stattgefunden. Das Thema Patienteninformation wurde hierbei ausführlich besprochen und es ist geplant, das Verständnis zum Konzept von PROMs und dem Projekt durch Beiträge in den jeweiligen Mitgliederzeitschriften (digital und/oder analog) zu fördern.
- **Nachfragen der Study Nurse beim Patienten:** Wichtig ist zu erwähnen, dass die Rücklaufquote durch Nachfragen der Study Nurse bei den Patienten beeinflusst und aus Erfahrung seitens Heartbeat auch deutlich erhöht werden kann.

A4 Bitte legen Sie eine Stellungnahme des zuständigen Evaluators vor, aus der hervorgeht, welche Auswirkungen die beantragte Fallzahlreduktion auf den erwarteten Erkenntnisgewinn hat, und reichen Sie ein aktualisiertes Evaluationskonzept unter Berücksichtigung der neuen Fallzahlen ein. Die Änderungen im Evaluationskonzept sind bitte kenntlich zu machen. Es ist zudem zu prüfen und darzulegen, ob sich aufgrund der Fallzahlreduktion ein reduzierter Arbeitsaufwand für die Evaluation ergibt.

B4 Erkenntnisgewinn bei reduzierter Fallzahl

Mit der prognostizierten Fallzahl von hochgerechnet 9.494 Patienten mit PROM-Erhebung von denen etwa 80% (etwa 7.597 Patienten) die Studienteilnahme bis zum Schluss fortsetzen, sowie den daraus resultierenden etwa 1.403 VBHC Patienten, ist eine Auswertung des primären Outcomes weiterhin möglich, da die benötigte Mindestfallzahl von 1.396 Patienten erreicht wird.

Die Reduktion der PROM-Patienten von etwa 26 %, (7.597 gegenüber bisher geschätzten 10.243 Patienten) wird voraussichtlich einen gewissen Einfluss auf die Tiefe der Subgruppenanalysen haben, allerdings ist davon auszugehen, dass bei der Effektgröße von 0,15 Standardabweichungen, analog zum primären Endpunkt, Subgruppen mit mindestens 18,4 % Anteil an der Gesamtfallzahl weiterhin untersucht werden können. Da die Effekte der Endpunkte aufgrund fehlender vergleichbarer Studien nur geschätzt sind, ist es erst bei der Auswertung möglich, die Struktur und Tiefe der Subgruppenanalyse festzulegen.

In dem ersten unter B1 genannten Beispiel zur Subgruppenanalyse über 80-jähriger Patienten würde die genannte Änderung der Fallzahlen (531 statt 700 Patienten) dazu führen, das bei gleichen Randbedingungen zur Fehlerwahrscheinlichkeit (5%) und Power (80%) eine geringfügig höhere Effektgröße erforderlich wäre als bisher angenommen (0,17 statt 0,15).

Beim zweiten Beispiel unter B1, dem möglicherweise zu untersuchenden Sekundäroutcomes des Auftretens von Komplikationen, ist bei einer Prävalenz des Outcomes von 4%, mit der zu erwartenden Gesamtfallzahl von jeweils 3.798 in Interventions- und Kontrollgruppe weiterhin ein Unterschied in der Größenordnung von ca. einem Prozentpunkt (1,19 % statt 1,07 %) nachzuweisen.

Arbeitsaufwand der Evaluation

Ein reduzierter Arbeitsaufwand durch die Verringerung der Fallzahlen ist nicht zu erwarten, da für die Evaluation nicht Anzahl der in der Studie eingeschlossenen Patienten, sondern der in der Struktur unveränderte Evaluationsplan ausschlaggebend ist. Eine mögliche Reduktion einzelner Subgruppenanalysen steht hierbei dem höheren Aufwand gegenüber, den durch den verkürzten Evaluationszeitraum notwendig gewordenen Teil-Datenexport zum Ende des Jahres 2021 mit dem zweiten Teildatenexport im Q3 des Jahres 2022 zusammenzuführen.

Zuständiger Methodiker für das Evaluationskonzept:
Dr. med. Thomas G. Grobe, MPH (aQua-Institut, Göttingen)

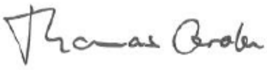

Göttingen, 24.09.2020	
Ort, Datum	Unterschrift
