# Supplementary material for: Electronic Patient-Reported Outcome Monitoring to Improve Quality of Life After Joint Replacement: Secondary Analysis of a Randomized Clinical Trial
Source: JAMA Netw Open. 2023 Sep 1;6(9):e2331301. doi: 10.1001/jamanetworkopen.2023.31301 (PMC10474554; doi:10.1001/jamanetworkopen.2023.31301)
Supplement: Supplement 2. — eFigure 1. Participant Flow in the “PROMoting Quality” Trial With Detailed Information on the Reasons for Exclusion eTable 1. Adjustments to the Study Protocol eTable 2. Absolute Intervention Alert Thresholds for Each PROM-Score eTable 3. Descriptive Statistics of the Compliance Corrected Study Population eTable 4. Descriptive Statistics of the Admission, Discharge and 12-Month Follow-up PROM-Scores eFigure 2. Raw PROM-Score Distributions (Baseline, 12 Months) for the Control and Intervention Groups in the Intention to Treat Population for the EQ-5D-5L eTable 5. Readmission and Reoperation Descriptive Statistics in the Intervention Versus the Control Group Separated per Joint Replacement Type eTable 6. Information on Critical Values and Reactions to PROM-Based Critical Value Alerts From Study Assistants and Patients -Critical Values and Alert Reactions eFigure 3. Count of Hip Replacement Patients Whose PROM-Scores Initiated Alerts per Intervention Time/Times eFigure 4. Count of Knee Replacement Patients Whose PROM-Scores Initiated Alerts per Intervention Time/Times eTable 7. Therapy Changes in the Intervention Group of Patients Who Visited a Physician and Were Called Versus Not Called eFigure 5. Classification of Notes by Study Nurses of Patients Who Were Not Called Following an Alert (n= 560) eTable 8. Difference in the PROM-Score-Change From Baseline to 12 Months Post-Surgery, Between Intervention and Control Group (Compliance Corrected Study Population) eTable 9. Mixed Effects Models With PROM-Score Changes as Outcomes, Controlling for Gender, Age, Mobilization and Admission PROM Scores as Fixed Effects and Hospital as Random Intercept (Intention to Treat Study Population) eTable 10. Mixed Effects Models With PROM-Score Changes as Outcomes, Controlling for Gender, Age, Mobilization and Admission PROM Scores as Fixed Effects and Hospital as Random Intercept (Compliance Corrected Study Population) eTable 11. Mixed Effects Models With PROM-Score Change Z-Scores as O [file jamanetwopen-e2331301-s002.pdf]

Supplementary Online Content

Steinbeck V, Langenberger B, Schöner L, et al. Electronic patient-reported outcome monitoring to improve quality of life after joint replacement: secondary analysis of a randomized clinical trial. *JAMA Netw Open.* 2023;6(9):e2331301. doi:10.1001/jamanetworkopen.2023.31301

eFigure 1. Participant Flow in the "PROMoting Quality" Trial With Detailed Information on the Reasons for Exclusion
eTable 1. Adjustments to the Study Protocol
eTable 2. Absolute Intervention Alert Thresholds for Each PROM-Score
eTable 3. Descriptive Statistics of the Compliance Corrected Study Population
eTable 4. Descriptive Statistics of the Admission, Discharge and 12-Month Follow-up PROM-Scores
eFigure 2. Raw PROM-Score Distributions (Baseline, 12 Months) for the Control and Intervention Groups in the Intention to Treat Population for the EQ-5D-5L
eTable 5. Readmission and Reoperation Descriptive Statistics in the Intervention Versus the Control Group Separated per Joint Replacement Type
eTable 6. Information on Critical Values and Reactions to PROM-Based Critical Value Alerts From Study Assistants and Patients -Critical Values and Alert Reactions
eFigure 3. Count of Hip Replacement Patients Whose PROM-Scores Initiated Alerts per Intervention Time/Times
eFigure 4. Count of Knee Replacement Patients Whose PROM-Scores Initiated Alerts per Intervention Time/Times
eTable 7. Therapy Changes in the Intervention Group of Patients Who Visited a Physician and Were Called Versus Not Called
eFigure 5. Classification of Notes by Study Nurses of Patients Who Were Not Called Following an Alert (n= 560)
eTable 8. Difference in the PROM-Score-Change From Baseline to 12 Months Post-Surgery, Between Intervention and Control Group (Compliance Corrected Study Population)
eTable 9. Mixed Effects Models With PROM-Score Changes as Outcomes, Controlling for Gender, Age, Mobilization and Admission PROM Scores as Fixed Effects and Hospital as Random Intercept (Intention to Treat Study Population)
eTable 10. Mixed Effects Models With PROM-Score Changes as Outcomes, Controlling for Gender, Age, Mobilization and Admission PROM Scores as Fixed Effects and Hospital as Random Intercept (Compliance Corrected Study Population)
eTable 11. Mixed Effects Models With PROM-Score Change Z-Scores as Outcomes, Controlling for Gender, Age, Mobilization and Admission PROM Scores as Fixed Effects and Hospital as Random Intercept (Intention to Treat Study Population)
eTable 12. Mixed Effects Models With PROM-Score Change Z-Scores as Outcomes, Controlling for Gender, Age, Mobilization and Admission PROM Scores as Fixed Effects and Hospital as Random Intercept (Compliance Corrected Study Population)

© 2023 Steinbeck V et al. *JAMA Network Open.*

This supplementary material has been provided by the authors to give readers additional information about their work.

© 2023 Steinbeck V et al. *JAMA Network Open.*

eFigure 1. Participant Flow in the "PROMoting Quality" Trial With Detailed Information on the Reasons for Exclusion

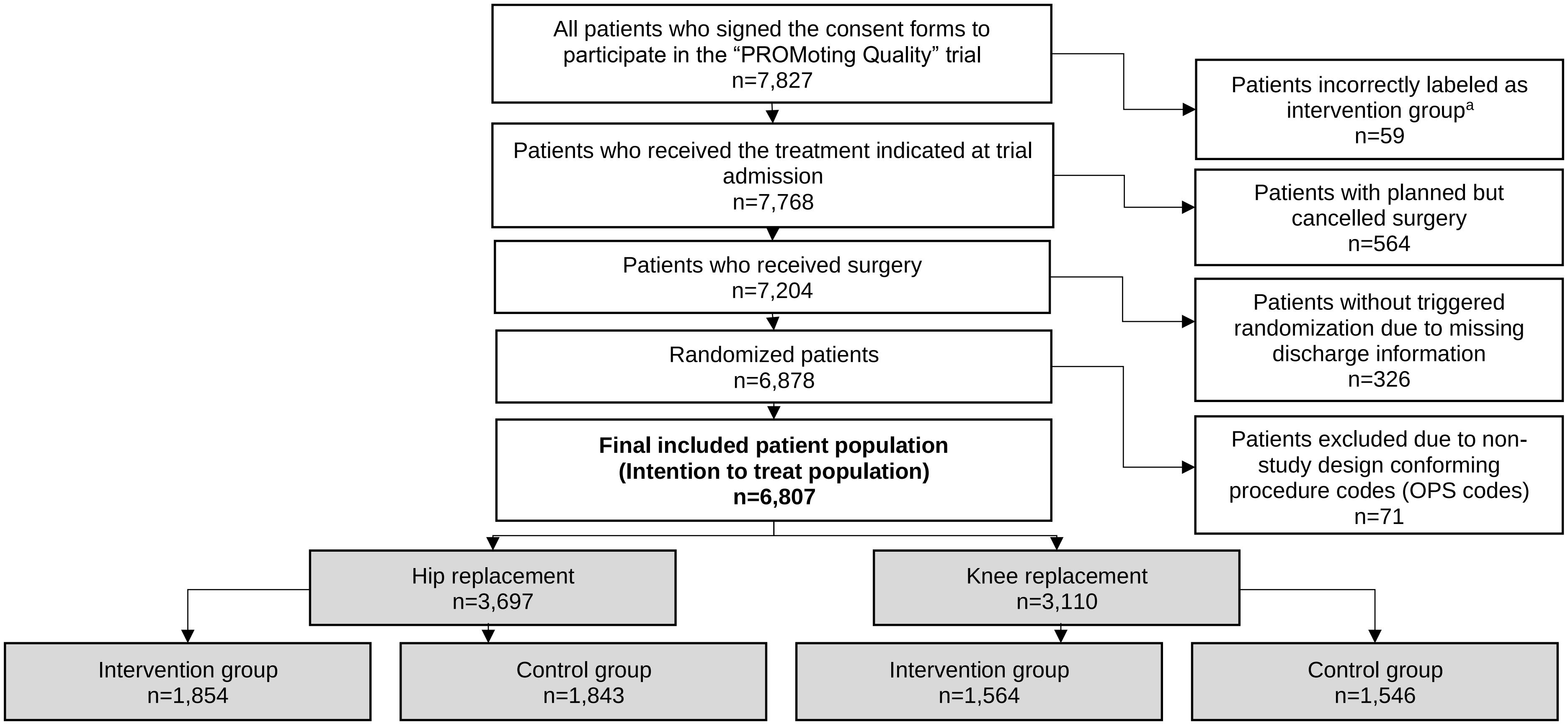

a Patients excluded in this step were originally designated to the control group. A study nurse manually sent PROMs to these participants (only in one hospital) at the intervention time points (at one-, three- and six-months). The digital system however did not initiate alerts, as this aspect could not be changed manually. Due to the difference in timepoints at which PROMs were answered among the 59 participants compared to the other control group participants, these patients were excluded.

© 2023 Steinbeck V et al. *JAMA Network Open.*

eTable 1. Adjustments to the Study Protocol

**Threshold alerts**	Originally any marginal deterioration in the HOOS-PS, KOOS-PS and EQ-5D-5L scores initiated an alert. After study nurses' feedback about many unnecessary calls (i.e. patients did not report health-related problems after being contacted), the relative threshold was adjusted to initiate an alert with a minimum 10-point deterioration in the HOOS-PS, KOOS-PS and 10% deterioration in EQ-5D-5L. These relative thresholds were calculated based on an analysis of comments made by the study assistants after contacting the patients. It was found that below a 10 point or percent deterioration, patients did not report any problems. Thus, the high administrative burden of the study assistants could be reduced without impacting the PROM-based intervention.
**Timeframe**	The study's timeframe was prolonged from the original recruitment period (October 2019 to September 2020) to last until December 2020. The follow-up lasted until March 2022 due to lower numbers of hip and knee-replacement surgeries being performed during waves of the COVID-19 pandemic. This extension was agreed to by the funder.
**OPS-codes**	Originally a list of surgical procedure codes (OPS-codes) was defined and communicated to study nurses. Unexpectedly, patients with other OPS-codes were also included in the study which initiated a closer look into which OPS-codes of the included ones fits to the target group of primary hip and knee replacements. The following OPS-codes were hence included:<br><br>Hip: 5-820.00, 5-820.01, 5-820.02, 5-820.20, 5-820.22, 5-820.8, 5-820.80, 5-820.81, 5-820.82, 5-820.9, 5-820.92, 5-820.93, 5-820.94, 5-820.95, 5-820.96, 5-820.X, 5-820.x0, 5-820.x1, 5-820.x2, 5-820.y<br><br>Knee: 5-822.0, 5-822.00, 5-822.01, 5-822.02, 5-822.g, 5-822.g0, 5-822.g1, 5-822.g2, 5-822.j, 5-822.j1, 5-822.j2, 5-822.k, 5-822.k0, 5-822.k1, 5-822.k2, 5-822.h1, 5-822.h2

© 2023 Steinbeck V et al. *JAMA Network Open.*

eTable 2. Absolute Intervention Alert Thresholds for Each PROM-Score

PROM Sets	Month 1[a]	Month 3[a]	Month 6[a]	Relative deterioration alert[b]	Relative threshold
EQ-5D-5L hip	0.37	0.64	0.74	Yes	10% worse than t-1
HOOS-PS	53.00	36.30	27.70	Yes	10% worse than t-1
EQ-5D-5L knee	0.36	0.51	0.70	Yes	10% worse than t-1
KOOS-PS	51.60	43.60	33.60	Yes	10% worse than t-1
PROMIS Depression	65.80	65.80	65.80	No	NA
PROMIS Fatigue	69.00	69.00	69.00	No	NA

a the absolute alert thresholds were set through a Delphi panel (Kuklinski et al. 2020)

b the relative change to a worse score initiated an alert for all PROM-scores with “Yes”. This table is based on the table published in the study protocol of the PROMoting Quality trial (Kuklinski et al. 2020)

© 2023 Steinbeck V et al. *JAMA Network Open.*

eTable 3. Descriptive Statistics of the Compliance Corrected Study Population

group	Hip replacement patients		Knee replacement patients	
	**intervention (N=1,649)**	**control (N=1,843)**	**intervention (N=1,366)**	**control (N=1,546)**
**Age**				
mean (SD)	65.7 (10.4)	65.7 (10.6)	65.9 (8.90)	65.9 (9.36)
**Gender (%)**				
female	929 (65.3)	1,036 (56.2)	738 (54.0)	830 (53.7)
male	720 (43.7)	807 (43.8)	628 (46.0)	716 (46.3)
**BMI group (%)**				
underweight	10 (0.6)	10 (0.5)	1 (0.1)	3 (0.2)
normal	513 (31.1)	588 (31.9)	223 (16.3)	222 (14.4)
overweight	650 (39.4)	699 (37.9)	491 (35.9)	618 (40.0)
obese	476 (28.9)	546 (29.6)	651 (47.7)	703 (45.5)
**Smoker (%)**				
no	1,423 (86.3)	1,547 (83.9)	1,200 (87.8)	1,336 (86.4)
yes	226 (13.7)	296 (16.1)	166 (12.2)	210 (13.6)
**Education (%)**				
no school	7 (0.4)	7 (0.4)	9 (0.7)	5 (0.3)
primary school	202 (12.2)	249 (13.5)	222 (16.3)	271 (17.5)
high/middle school	964 (58.5)	1,033 (56.1)	805 (58.9)	927 (60.0)
university	476 (28.9)	554 (30.1)	330 (24.2)	343 (22.2)
**Living situation (%)**				
alone	375 (22.7)	414 (22.5)	263 (19.3)	289 (18.7)
care facility	4 (0.2)	7 (0.4)	7 (0.5)	9 (0.6)
with a partner/family/friends	1,261 (76.5)	1,408 (76.4)	1,093 (80.0)	1,233 (79.8)
other	9 (0.5)	14 (0.8)	3 (0.2)	15 (1.0)
**Job (%)**				
working	524 (31.8)	618 (33.5)	404 (29.6)	468 (30.3)
voluntarily not working including retirement	950 (57.6)	1,034 (56.1)	792 (58.0)	875 (56.6)
looking for work	18 (1.1)	15 (0.8)	10 (0.7)	23 (1.5)
unable to work	177 (9.8)	176 (9.6)	160 (11.7)	180 (11.6)

© 2023 Steinbeck V et al. *JAMA Network Open.*

group	Hip replacement patients		Knee replacement patients	
	**intervention (N=1,649)**	**control (N=1,843)**	**intervention (N=1,366)**	**control (N=1,546)**
**mobilization after surgery (%)**				
within 6 hours	755 (45.8)	854 (46.3)	716 (45.8)	709 (45.9)
within 12 hours	503 (30.5)	496 (26.9)	410 (30.0)	425 (27.5)
within 24 hours	359 (21.8)	443 (24.0)	283 (20.7)	356 (23.0)
within 48 hours	22 (1.3)	35 (1.9)	36 (2.6)	46 (3.0)
after 48 hours	10 (0.6)	15 (0.8)	9 (0.7)	10 (0.6)
**Readmission (%)**				
no	1,609 (97.6)	1,789 (97.1)	1,335 (97.7)	1,506 (97.4)
within 30 days post-surgery	18 (1.1)	28 (1.5)	7 (0.5)	21 (1.4)
between 30-90 days post-surgery	22 (1.3)	26 (1.4)	24 (1.7)	19 (1.2)
**reoperation within 12 months post-surgery (%)**				
no	1,617 (98.1)	1,798 (97.6)	1,336 (97.8)	1,504 (97.3)
yes	32 (1.9)	45 (2.4)	30 (2.2)	42 (2.7)
**PROM baseline score mean (SD)[a]**				
EQ-5D-5L	0.599 (0.260)	0.603 (0.256)	0.628 (0.258)	0.623 (0.245)
EQ-VAS	56.6 (19.9)	57.1 (19.7)	58.9 (19.5)	57.9 (19.1)
HOOS/KOOS-PS	48.1 (16.3)	47.1 (16.1)	43.1 (13.4)	43.0 (12.0)
PROMIS-fatigue	49.2 (9.84)	49.2 (9.98)	48.2 (10.1)	48.1 (9.54)
PROMIS-depression	49.7 (8.32)	49.8 (8.26)	49.3 (8.40)	49.4 (8.16)

a PROM-scores have different score ranges: -0.661-1 for the EQ-5D-5L, 0-100 for the EQ-VAS, 0-100 for the HOOS-PS and KOOS-PS, 33.7-75.8 for the PROMIS-fatigue and 41-79.4 for the PROMIS-depression. Higher values in the EQ-VAS and EQ-5D-5L indicate better health levels, whereas lower values in H/KOOS-PS, PROMIS-fatigue, and depression indicate better health (less health impairment).

© 2023 Steinbeck V et al. *JAMA Network Open.*

eTable 4. Descriptive Statistics of the Admission, Discharge and 12-Month Follow-up PROM-Scores

**procedure**	**hip replacement patients**				**knee replacement patients**			
**group**	**Intervention (n = 1,854)**		**Control (n = 1,843)**		**Intervention (n = 1,564)**		**Control (n = 1,546)**	
**variable**	**Mean**	**SD**	**Mean**	**SD**	**Mean**	**SD**	**Mean**	**SD**
**admission PROM-score**								
EQ-VAS	56.529	20.035	57.096	19.694	58.652	19.379	57.926	19.135
EQ-5D-5L	0.594	0.263	0.603	0.256	0.625	0.257	0.623	0.245
HOOS/KOOS-PS	48.35	16.398	47.109	16.099	43.135	13.339	42.983	12.026
PROMIS-depression	49.704	8.354	49.835	8.263	49.367	8.393	49.384	8.156
PROMIS-fatigue	49.163	9.912	49.209	9.975	48.319	10.105	48.145	9.544
**12-months post-surgery**								
EQ-VAS	75.505	17.148	73.974	18.45	72.198	17.519	70.235	18.423
EQ-5D-5L	0.881	0.158	0.874	0.169	0.851	0.182	0.843	0.191
HOOS/KOOS-PS	14.557	13.304	15.179	14.246	25.787	13.417	26.625	12.869
PROMIS-depression	46.926	7.403	47.622	7.633	47.483	7.7	47.744	7.831
PROMIS-fatigue	44.839	8.652	45.582	8.727	45.595	8.932	46.27	8.791

© 2023 Steinbeck V et al. *JAMA Network Open.*

eFigure 2. Raw PROM-Score Distributions (Baseline, 12 Months) for the Control and Intervention Groups in the Intention to Treat Population for the EQ-5D-5L[a]

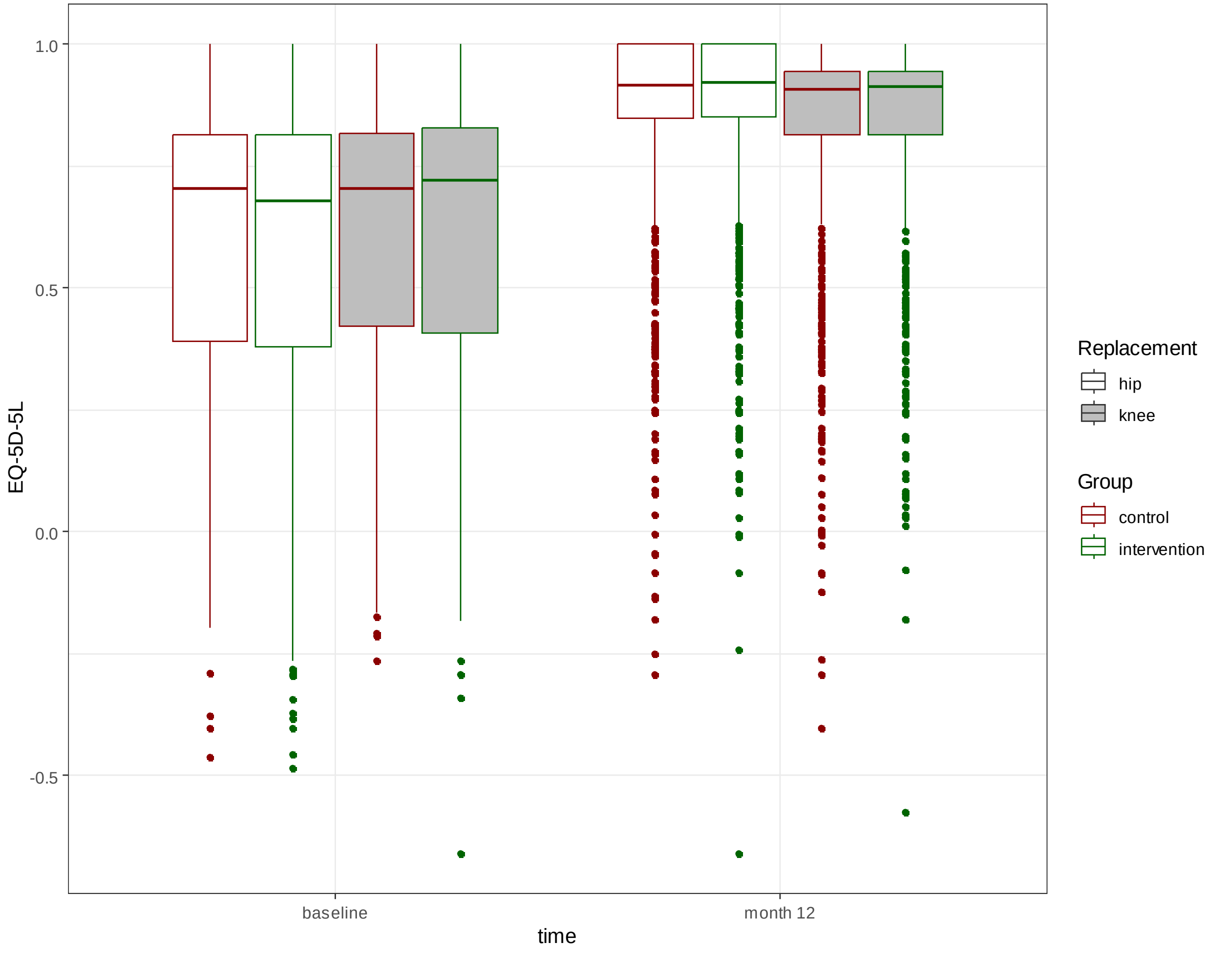

a higher value in the EQ-5D-5L indicates better health levels

© 2023 Steinbeck V et al. *JAMA Network Open.*

eTable 5. Readmission and Reoperation Descriptive Statistics in the Intervention Versus the Control Group Separated per Joint Replacement Type

**Hip replacement patients**					
**Group**	**control**		**intervention**		**Chi-Square Test p-value**
**Variable**	**N**	**Percent**	**N**	**Percent**	
**readmission**	1843		1854		0.544
… no	1789	97%	1809	98%	
... readmission within 30 days	28	2%	19	1%	
... readmission between 30 to 60 days	10	1%	8	0%	
... readmission between 60 to 90 days	16	1%	18	1%	
**reoperation**	1843		1854		0.488
... no	1798	98%	1816	98%	
... yes	45	2%	38	2%	
**Knee replacement patients**					
**readmission**	1546		1564		0.140
… no	1506	97%	1531	98%	
... readmission within 30 days	21	1%	9	1%	
... readmission between 30 to 60 days	8	1%	10	1%	
... readmission between 60 to 90 days	11	1%	14	1%	
**reoperation**	1546		1564		0.388
... no	1504	97%	1530	98%	
... yes	42	3%	34	2%	

© 2023 Steinbeck V et al. *JAMA Network Open.*

eTable 6. Information on Critical Values and Reactions to PROM-Based Critical Value Alerts From Study Assistants and Patients - Critical Values and Alert Reactions

procedure	***Hip replacement patients***			***Knee replacement patients***		
month	**month 1**	**month 3**	**month 6**	**month 1**	**month 3**	**month 6**
**number of patients in the intervention group**						
critical value information available	1,772	1,773	1,709	1,486	1,485	1,435
**number of patients in the intervention group (% of patients with information on critical values)**						
patients with critical values	157 (9%)	454 (26%)	424 (25%)	157 (11%)	386 (26%)	461 (32%)
patients without critical values	1615 (91%)	1319 (74%)	1285 (75%)	1329 (89%)	1099 (74%)	974 (68%)
**number of those with critical values (% of critical value patients)**						
call to patient by study nurse	114 (73%)	319 (70%)	321 (76%)	111 (71%)	287 (74%)	316 (69%)
data transfer to patients	29 (18%)	88 (19%)	83 (20%)	32 (20%)	74 (19%)	105 (23%)
data transfer to physician	24 (15%)	43 (9%)	26 (6%)	28 (18%)	49 (13%)	25 (5%)
physician visit	103 (66%)	299 (66%)	259 (61%)	102 (65%)	288 (75%)	323 (70%)

© 2023 Steinbeck V et al. *JAMA Network Open.*

eFigure 3. Count of Hip Replacement Patients Whose PROM-Scores Initiated Alerts per Intervention Time/Times[a]

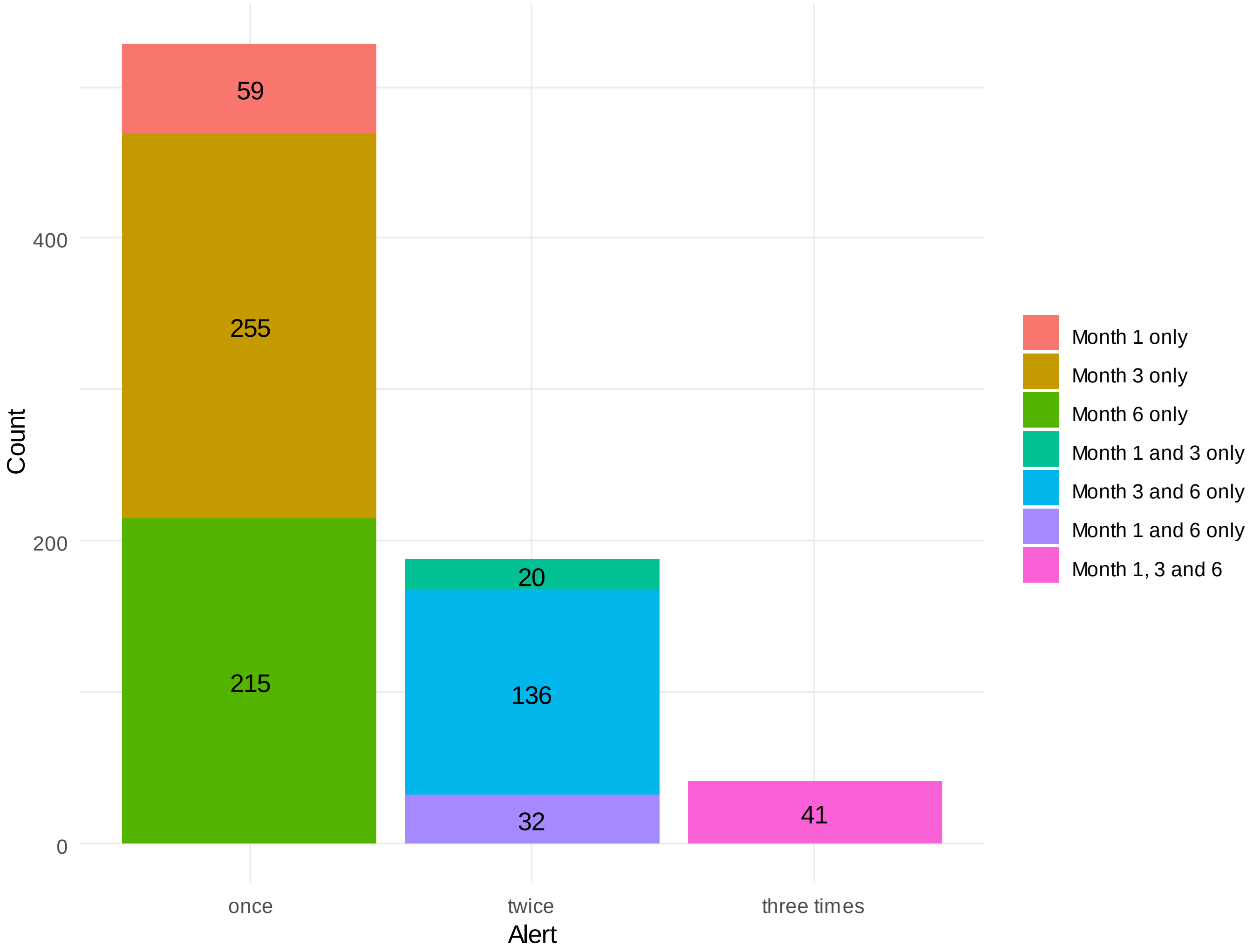

a the total number of patients with critical values (alerts) were 157, 454 and 424 for month one, three and six respectively. The total number of patients who did not have a critical value (no alert) were 1615, 1099 and 974 respectively.

© 2023 Steinbeck V et al. *JAMA Network Open.*

eFigure 4. Count of Knee Replacement Patients Whose PROM-Scores Initiated Alerts per Intervention Time/Times[a]

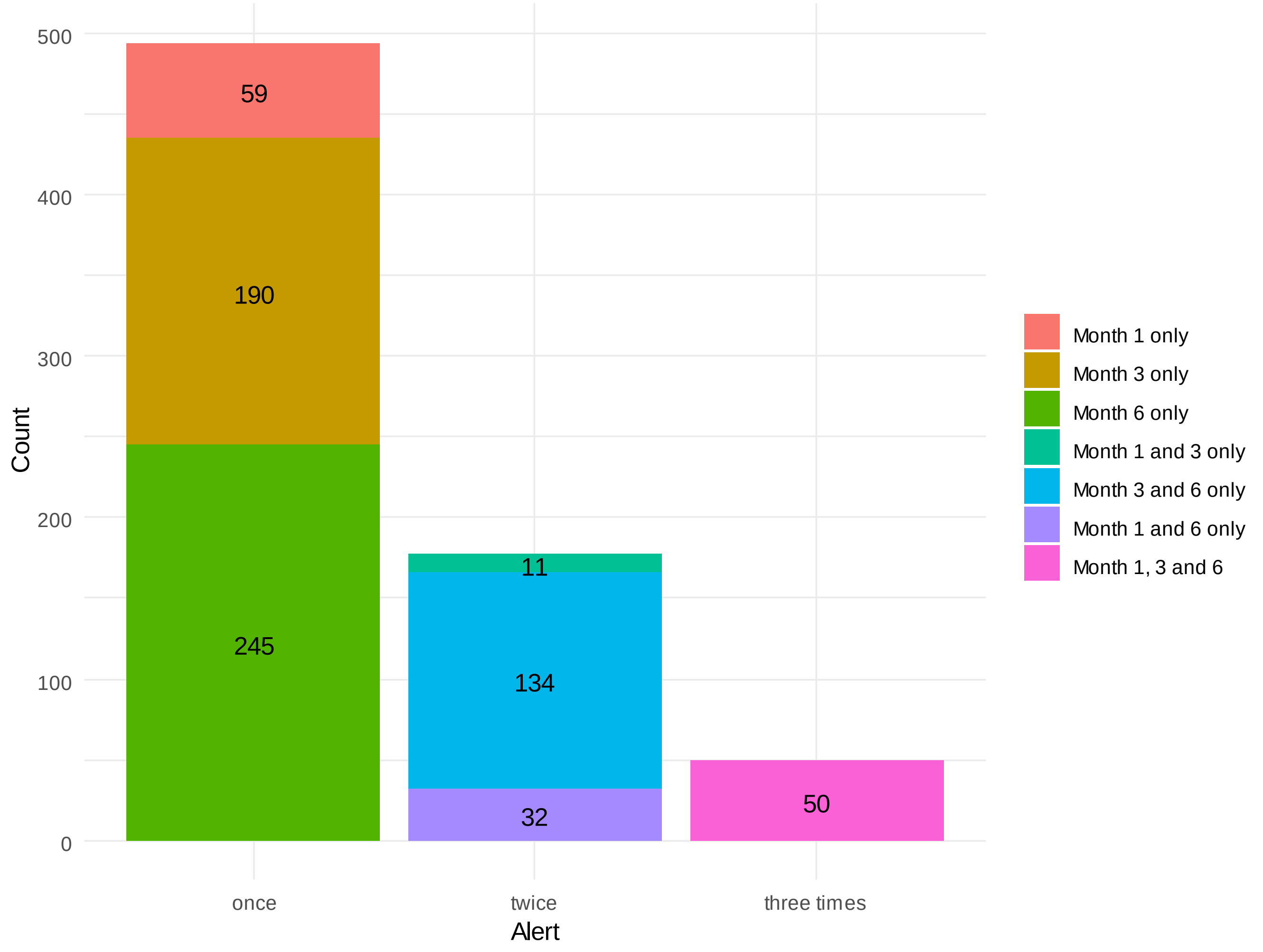

a the total number of patients with critical values (alerts) were 157, 386 and 461 for month one, three and six respectively. The total number of patients who did not have a critical value (no alert) were 1329, 1319 and 1285 respectively.

© 2023 Steinbeck V et al. *JAMA Network Open.*

eTable 7. Therapy Changes in the Intervention Group of Patients Who Visited a Physician and Were Called Versus Not Called

**procedure**	**hip replacement patients**			**knee replacement patients**		
Call in:[a]	***month 1***	***month 3***	***month 6***	***month 1***	***month 3***	***month 6***
Reaction by:[b]	***month 3***	***month 6***	***month 12***	***month 3***	***month 6***	***month 12***
**discussed PROs with treating physician**						
yes	30 (36.6%)	72 (38.7%)	58 (36.5%)	30 (33.0%)	49 (26.6%)	43 (24.3%)
no	52 (63.4%)	114 (61.3%)	101 (63.5%)	61 (67.0%)	135 (73.4%)	134 (75.7%)
**change in medication**						
yes	20 (24.4%)	49 (26.3%)	38 (23.9%)	41 (45.1%)	42 (22.8%)	48 (27.1%)
no	62 (75.6%)	137 (73.7%)	121 (76.1%)	50 (54.9%)	142 (77.2%)	129 (72.9%)
**change in physiotherapy**						
yes	31 (37.8%)	86 (46.2%)	64 (40.3%)	34 (37.4%)	75 (40.8%)	62 (35.0%)
no	51 (62.2%)	100 (53.8%)	95 (59.7%)	57 (62.6%)	109 (59.2%)	115 (65.0%)
**change of aftercare physician**						
yes	8 (9.8%)	16 (8.6%)	NA	3 (3.3%)	7 (3.8%)	NA
no	74 (90.2%)	170 (91.4%)	NA	88 (96.7%)	177 (96.2%)	NA
No call in:[a]	***month 1***	***month 3***	***month 6***	***month 1***	***month 3***	***month 6***
Reaction by:[b]	***month 3***	***month 6***	***month 12***	***month 3***	***month 6***	***month 12***
**discussed PROs with treating physician**						
yes	267 (27.8%)	191 (28.9%)	143 (27.7%)	225 (26.1%)	210 (32.4%)	140 (31.9%)
no	693 (72.2%)	471 (71.1%)	373 (72.3%)	637 (73.9%)	439 (67.6%)	299 (68.1%)
**change in medication**						
yes	224 (23.3%)	106 (16.0%)	72 (14.0%)	239 (27.7%)	151 (23.3%)	71 (16.2%)
no	736 (76.7%)	556 (84.0%)	444 (86.0%)	623 (72.3%)	498 (76.7%)	368 (83.8%)
**change in physiotherapy**						
yes	407 (42.4%)	233 (35.2%)	153 (29.7%)	356 (41.3%)	252 (38.8%)	133 (30.3%)
no	553 (57.6%)	429 (64.8%)	363 (70.3%)	506 (58.7%)	397 (61.2%)	306 (69.7%)
**change of aftercare physician**						
yes	24 (2.5%)	24 (3.6%)	NA	26 (3.0%)	28 (4.3%)	NA
no	936 (97.5%)	638 (96.4%)	NA	836 (97.0%)	621 (95.7%)	NA

a Call means that a phone call took place in which the study nurse called the patient who had an alert at month 1,3,6 (one of the intervention steps). No call means that no call took place in the respective month.

b Reaction by refers to the month in which a change in therapy was reported. This row specified the next measurement time after the call taking place/not taking place, as the reaction based on the call could have only be visible in the data after the call at the next measurement time.

© 2023 Steinbeck V et al. *JAMA Network Open.*

eFigure 5. Classification of Notes by Study Nurses of Patients Who Were Not Called Following an Alert (n= 560)

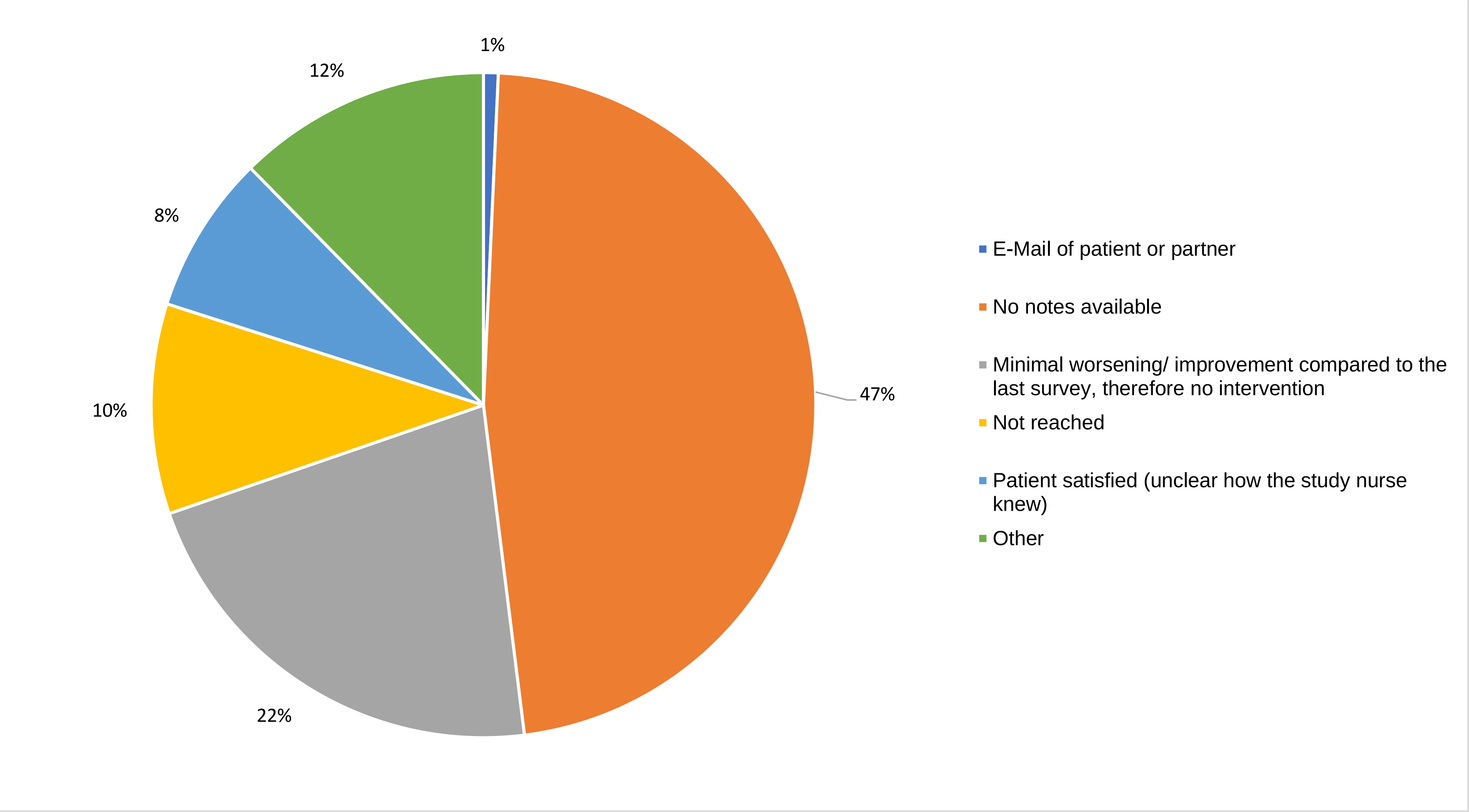

© 2023 Steinbeck V et al. *JAMA Network Open.*

eTable 8. Difference in the PROM-Score-Change From Baseline to 12 Months Post-Surgery, Between Intervention and Control Group (Compliance Corrected Study Population)

	intervention			control			mean between-group difference		
variable	N	Mean change in PROM-score[a]	SD	N	Mean change in PROM-score[a]	SD	in points[b]	95% confidence interval	p-value[c]
**Hip replacement patients**									
EQ-5D-5L	1,649	0.283	0.262	1,843	0.271	0.262	0.012	- 0.004 to 0.030	0.144
EQ-VAS	1,649	19.14	22.35	1,843	16.88	22.56	2.26	0.76 to 3.75	0.003***
HOOS-PS	1,649	-33.78	17.68	1,843	-31.93	17.99	-1.85	-3.04 to -0.66	0.002***
PROMIS-depression	1,649	-2.79	7.85	1,843	-2.21	7.90	-0.58	-1.10 to -0.05 to	0.031**
PROMIS-fatigue	1,649	-4.45	9.32	1,843	-3.63	9.17	-0.82	-1.44 to -0.21	0.009***
**Knee replacement patients**									
EQ-5D-5L	1,366	0.223	0.258	1,546	0.220	0.251	0.003	-0.015 to 0.022	0.697
EQ-VAS	1,366	13.65	22.19	1,546	12.31	21.27	1.34	-0.25 to 2.92	0.098*
KOOS-PS	1,366	-17.50	14.60	1,546	-16.36	13.60	-1.14	-2.17 to -0.12	0.028**
PROMIS-depression	1,366	-1.89	7.81	1,546	-1.64	7.71	-0.25	-0.82 to 0.31	0.384
PROMIS-fatigue	1,366	-2.78	9.11	1,546	-1.88	8.81	-0.90	-1.55 to 0.25	0.007***

a The score ranges for the PROMs are: -0.661- 1.0 for the EQ-5D-5L, 0-100 for the EQ-VAS, 0-100 for the HOOS-PS and KOOS-PS, 33.7-75.8 for the PROMIS-fatigue and 41-79.4 for the PROMIS-depression. Due to the low score range of the EQ-5D-5L, three digits after the decimal point are reported

b The mean difference in points refers to the difference between intervention and control group in terms of their mean change in the respective PROM-score

© 2023 Steinbeck V et al. *JAMA Network Open.*

eTable 9. Mixed Effects Models With PROM-Score Changes as Outcomes, Controlling for Gender, Age, Mobilization and Admission PROM Scores as Fixed Effects and Hospital as Random Intercept (Intention to Treat Study Population)

Hip replacement patients

	**EQ-5D-5L**			**EQ-VAS**			**HOOS-PS**			**PROMIS-fatigue**			**PROMIS-depression**		
Predictors	*Estimates*	*CI*	*p*	*Estimates*	*CI*	*p*	*Estimates*	*CI*	*p*	*Estimates*	*CI*	*p*	*Estimates*	*CI*	*p*
(Intercept)	0.743	0.675 – 0.811	**<0.001**	65.00	57.56 – 72.44	**<0.001**	4.63	-1.24 – 10.49	0.122	27.50	23.92 – 31.08	**<0.001**	26.60	23.43 – 29.77	**<0.001**
group [intervention]	0.008	-0.002 – 0.018	0.113	1.66	0.58 – 2.74	**0.003**	-0.82	-1.65 – 0.00	0.051	-0.65	-1.12 – -0.18	**0.007**	-0.60	-1.01 – -0.18	**0.005**
gender [male]	0.004	-0.006 – 0.014	0.450	-0.26	-1.35 – 0.84	0.647	-0.17	-1.01 – 0.68	0.702	0.57	0.08 – 1.06	**0.022**	-0.17	-0.59 – 0.26	0.444
age	-0.001	-0.001 – -0.001	**<0.001**	-0.24	-0.30 – -0.19	**<0.001**	0.15	0.11 – 0.19	**<0.001**	-0.02	-0.04 – 0.00	0.118	0.01	-0.01 – 0.03	0.180
mobilisation [within 12 hours after surgery]	0.074	0.014 – 0.135	**0.016**	8.75	2.22 – 15.27	**0.009**	-11.10	-16.10 – -6.10	**<0.001**	-3.95	-6.81 – -1.09	**0.007**	-3.62	-6.12 – -1.13	**0.004**
mobilisation [within 24 hours after surgery]	0.059	-0.001 – 0.120	0.056	7.88	1.33 – 14.43	**0.018**	-9.60	-14.62 – -4.59	**<0.001**	-2.92	-5.79 – -0.05	**0.046**	-3.01	-5.52 – -0.50	**0.019**
mobilisation [within 48 hours after surgery]	0.032	-0.039 – 0.103	0.382	5.18	-2.52 – 12.87	0.187	-6.84	-12.74 – -0.95	**0.023**	-2.17	-5.54 – 1.19	0.206	-1.19	-4.13 – 1.76	0.429
mobilisation [within 6 hours after surgery]	0.082	0.022 – 0.142	**0.007**	10.10	3.61 – 16.59	**0.002**	-12.24	-17.22 – -7.27	**<0.001**	-3.94	-6.79 – -1.10	**0.007**	-3.61	-6.09 – -1.13	**0.004**
EQ-5D-5L admission	-0.811	-0.831 – -0.792	**<0.001**												

© 2023 Steinbeck V et al. *JAMA Network Open.*

EQ-VAS admission				-0.73	-0.75 – -0.70	**<0.001**									
HOOS-PS admission							-0.74	-0.77 – -0.71	**<0.001**						
PROMIS-fatigue admission										-0.53	-0.56 – -0.51	**<0.001**			
PROMIS-depression admission													-0.53	-0.55 – -0.50	**<0.001**
**Random Effects**															
σ^2	0.02			279.03			163.70			53.42			40.84		
τ_{00}	0.00 $_{hospital}$			3.20 $_{hospital}$			4.99 $_{hospital}$			1.64 $_{hospital}$			0.50 $_{hospital}$		
ICC	0.01			0.01			0.03			0.03			0.01		
N	9 $_{hospital}$			9 $_{hospital}$			9 $_{hospital}$			9 $_{hospital}$			9 $_{hospital}$		
Observations	3,697			3,697			3,697			3,697			3,697		
Marginal R^2 / Conditional R^2	0.646 / 0.650			0.437 / 0.443			0.461 / 0.477			0.342 / 0.362			0.318 / 0.326		

Knee replacement patients

	**EQ-5D-5L**			**EQ-VAS**			**KOOS-PS**			**PROMIS-fatigue**			**PROMIS-depression**		
Predictors	*Estimates*	*CI*	*p*	*Estimates*	*CI*	*p*	*Estimates*	*CI*	*p*	*Estimates*	*CI*	*p*	*Estimates*	*CI*	*p*
(Intercept)	0.518	0.434 – 0.603	**<0.001**	46.58	38.35 – 54.80	**<0.001**	16.16	10.06 – 22.26	**<0.001**	29.27	25.28 – 33.26	**<0.001**	24.73	21.15 – 28.32	**<0.001**
group [intervention]	0.007	-0.005 – 0.019	0.248	1.71	0.53 – 2.90	**0.005**	-0.80	-1.63 – 0.03	0.057	-0.71	-1.23 – -0.20	**0.006**	-0.23	-0.69 – 0.22	0.313
gender [male]	0.010	-0.002 – 0.023	0.096	1.23	0.02 – 2.43	**0.046**	-1.03	-1.88 – -0.19	**0.016**	-0.23	-0.75 – 0.30	0.400	-0.77	-1.24 – -0.30	**0.001**
age	0.001	0.000 – 0.002	**0.012**	-0.03	-0.10 – 0.03	0.308	-0.06	-0.11 – -0.02	**0.007**	-0.06	-0.09 – -0.03	**<0.001**	-0.01	-0.03 – 0.02	0.514
mobilisation [within 12	0.108	0.038 – 0.179	**0.003**	7.11	0.25 – 13.97	**0.042**	-3.22	-8.02 – 1.57	0.187	-3.02	-5.99 – -0.04	**0.047**	-1.33	-3.97 – 1.30	0.321

© 2023 Steinbeck V et al. *JAMA Network Open.*

hours after surgery]															
mobilisation [within 24 hours after surgery]	0.087	0.017 – 0.158	**0.015**	6.51	-0.37 – 13.39	0.064	-1.81	-6.61 – 2.99	0.460	-2.49	-5.47 – 0.50	0.102	-1.02	-3.66 – 1.62	0.450
mobilisation [within 48 hours after surgery]	0.046	-0.032 – 0.125	0.253	2.77	-4.87 – 10.40	0.478	-1.24	-6.57 – 4.10	0.649	-1.61	-4.92 – 1.70	0.341	0.70	-2.23 – 3.63	0.640
mobilisation [within 6 hours after surgery]	0.104	0.034 – 0.174	**0.004**	7.53	0.69 – 14.36	**0.031**	-3.95	-8.73 – 0.83	0.105	-2.88	-5.85 – 0.08	0.057	-1.29	-3.91 – 1.33	0.335
EQ-5D-5L admission	-0.740	-0.764 – -0.715	**<0.001**												
EQ-VAS admission				-0.69	-0.72 – -0.66	**<0.001**									
KOOS-PS admission							-0.57	-0.61 – -0.54	**<0.001**						
PROMIS-fatique admission										-0.50	-0.53 – -0.48	**<0.001**			
PROMIS-depression admission													-0.49	-0.52 – -0.46	**<0.001**
**Random Effects**															
σ^2	0.03			283.95			137.79			53.04			41.67		
τ_{00}	0.00 hospital			1.73 hospital			4.32 hospital			1.76 hospital			0.55 hospital		
ICC	0.01			0.01			0.03			0.03			0.01		
N	9 hospital			9 hospital			9 hospital			9 hospital			9 hospital		
Observations	3,110			3,110			3,110			3,110			3,110		

© 2023 Steinbeck V et al. *JAMA Network Open.*

Marginal R^2 / Conditional R^2	0.529 / 0.534	0.380 / 0.384	0.267 / 0.289	0.303 / 0.326	0.272 / 0.282

© 2023 Steinbeck V et al. *JAMA Network Open.*

eTable 10. Mixed Effects Models With PROM-Score Changes as Outcomes, Controlling for Gender, Age, Mobilization and Admission PROM Scores as Fixed Effects and Hospital as Random Intercept (Compliance Corrected Study Population)

Hip replacement patients

	EQ-5D-5L			EQ-VAS			HOOS-PS			PROMIS-fatigue			PROMIS-depression		
Predictors	*Estimates*	*CI*	*p*	*Estimates*	*CI*	*p*	*Estimates*	*CI*	*p*	*Estimates*	*CI*	*p*	*Estimates*	*CI*	*p*
(Intercept)	0.745	0.674 – 0.815	**<0.001**	65.17	57.48 – 72.86	**<0.001**	4.78	-1.31 – 10.88	0.124	27.52	23.81 – 31.23	**<0.001**	26.79	23.49 – 30.09	**<0.001**
group [intervention]	0.008	-0.002 – 0.019	0.121	1.80	0.67 – 2.92	**0.002**	-0.89	-1.76 – -0.03	**0.043**	-0.68	-1.17 – -0.19	**0.007**	-0.56	-1.00 – -0.13	**0.010**
gender [male]	0.003	-0.008 – 0.014	0.575	-0.40	-1.54 – 0.74	0.492	-0.11	-1.00 – 0.78	0.808	0.53	0.02 – 1.04	**0.041**	-0.19	-0.64 – 0.25	0.399
age	-0.001	-0.001 – -0.000	**<0.001**	-0.24	-0.30 – -0.19	**<0.001**	0.15	0.11 – 0.19	**<0.001**	-0.02	-0.04 – 0.01	0.133	0.01	-0.01 – 0.03	0.227
mobilisation [within 12 hours after surgery]	0.080	0.018 – 0.143	**0.012**	8.61	1.88 – 15.35	**0.012**	-11.47	-16.65 – -6.28	**<0.001**	-4.08	-7.03 – -1.12	**0.007**	-3.68	-6.26 – -1.10	**0.005**
mobilisation [within 24 hours after surgery]	0.065	0.002 – 0.128	**0.042**	7.84	1.08 – 14.60	**0.023**	-9.99	-15.19 – -4.79	**<0.001**	-3.07	-6.03 – -0.10	**0.043**	-3.06	-5.65 – -0.47	**0.021**
mobilisation [within 48 hours after surgery]	0.041	-0.033 – 0.115	0.277	5.76	-2.21 – 13.73	0.156	-7.45	-13.58 – -1.32	**0.017**	-2.53	-6.03 – 0.97	0.156	-1.49	-4.55 – 1.56	0.338
mobilisation [within 6 hours after surgery]	0.087	0.025 – 0.149	**0.006**	9.99	3.29 – 16.69	**0.003**	-12.50	-17.65 – -7.35	**<0.001**	-3.93	-6.87 – -1.00	**0.009**	-3.61	-6.18 – -1.04	**0.006**
EQ-5D-5L admission	-0.813	-0.834 – -0.793	**<0.001**												

© 2023 Steinbeck V et al. *JAMA Network Open.*

EQ-VAS admission				-0.73	-0.76 – -0.70	**<0.001**									
HOOS-PS admission							-0.74	-0.77 – -0.71	**<0.001**						
PROMIS-fatigue admission										-0.53	-0.56 – -0.50	**<0.001**			
PROMIS-depression admission													-0.53	-0.56 – -0.50	**<0.001**
**Random Effects**															
σ^2	0.02			285.66			168.93			54.92			41.98		
τ_{00}	0.00 hospital			3.35 hospital			5.18 hospital			1.62 hospital			0.61 hospital		
ICC	0.01			0.01			0.03			0.03			0.01		
N	9 hospital			9 hospital			9 hospital			9 hospital			9 hospital		
Observations	3,492			3,492			3,492			3,492			3,492		
Marginal R^2 / Conditional R^2	0.639 / 0.643			0.431 / 0.438			0.453 / 0.469			0.333 / 0.353			0.312 / 0.322		

Knee replacement patients

	**EQ-5D-5L**			**EQ-VAS**			**KOOS-PS**			**PROMIS-fatigue**			**PROMIS-depression**		
Predictors	*Estimates*	*CI*	*p*	*Estimates*	*CI*	*p*	*Estimates*	*CI*	*p*	*Estimates*	*CI*	*p*	*Estimates*	*CI*	*p*
(Intercept)	0.511	0.418 – 0.606	**<0.001**	44.91	35.73 – 54.09	**<0.001**	15.49	8.77 – 22.22	**<0.001**	27.90	23.55 – 32.24	**<0.001**	24.30	20.37 – 28.23	**<0.001**
group [intervention]	0.005	-0.007 – 0.018	0.412	1.86	0.61 – 3.11	**0.004**	-0.94	-1.81 – -0.07	**0.035**	-0.73	-1.27 – -0.20	**0.007**	-0.22	-0.70 – 0.26	0.365
gender [male]	0.009	-0.003 – 0.023	0.146	1.02	-0.24 – 2.29	0.113	-1.10	-1.99 – -0.22	**0.015**	-0.10	-0.65 – 0.45	0.721	-0.73	-1.23 – -0.24	**0.003**

© 2023 Steinbeck V et al. *JAMA Network Open.*

age	0.001	0.004 – 0.001	**0.038**	-0.04	-0.10 – 0.03	0.317	-0.06	-0.11 – -0.01	**0.021**	-0.06	-0.09 – -0.03	**<0.001**	-0.01	-0.03 – 0.02	0.681
mobilisation [within 12 hours after surgery]	0.117	0.036 – 0.197	**0.005**	8.65	0.79 – 16.50	**0.031**	-3.07	-8.54 – 2.41	0.272	-1.89	-5.26 – 1.48	0.272	-1.26	-4.26 – 1.75	0.412
mobilisation [within 24 hours after surgery]	0.098	0.017 – 0.178	**0.018**	8.27	0.40 – 16.14	**0.040**	-1.83	-7.32 – 3.65	0.512	-1.52	-4.90 – 1.86	0.378	-0.99	-4.00 – 2.02	0.520
mobilisation [within 48 hours after surgery]	0.053	-0.035 – 0.141	0.242	4.34	-4.27 – 12.95	0.323	-1.16	-7.17 – 4.84	0.704	-0.75	-4.44 – 2.95	0.692	0.63	-2.67 – 3.92	0.709
mobilisation [within 6 hours after surgery]	0.114	0.034 – 0.194	**0.005**	9.22	1.39 – 17.05	**0.021**	-3.98	-9.44 – 1.48	0.153	-1.83	-5.19 – 1.54	0.287	-1.24	-4.24 – 1.75	0.416
EQ-5D-5L admission	-0.734	-0.759 – -0.708	**<0.001**												
EQ-VAS admission				-0.68	-0.72 – -0.65	**<0.001**									
KOOS-PS admission							-0.57	-0.60 – -0.53	**<0.001**						
PROMIS-fatigue admission										-0.49	-0.52 – -0.46	**<0.001**			
PROMIS-depression admission													-0.49	-0.52 – -0.46	**<0.001**
**Random Effects**															
σ^2	0.03			295.34			142.89			54.13			43.01		
τ_{00}	0.00 $_{hospital}$			2.15 $_{hospital}$			4.15 $_{hospital}$			1.92 $_{hospital}$			0.73 $_{hospital}$		
ICC	0.01			0.01			0.03			0.03			0.02		
N	9 $_{hospital}$			9 $_{hospital}$			9 $_{hospital}$			9 $_{hospital}$			9 $_{hospital}$		

© 2023 Steinbeck V et al. *JAMA Network Open.*

Observations	2,912	2,912	2,912	2,912	2,912
Marginal R^2 / Conditional R^2	0.516 / 0.522	0.370 / 0.375	0.256 / 0.277	0.290 / 0.315	0.263 / 0.275

© 2023 Steinbeck V et al. *JAMA Network Open.*

eTable 11. Mixed Effects Models With PROM-Score Change Z-Scores as Outcomes, Controlling for Gender, Age, Mobilization and Admission PROM Scores as Fixed Effects and Hospital as Random Intercept (Intention to Treat Study Population)

Hip replacement patients

	**EQ total Z**			**EQ-VAS Z**			**HOOS Z**			**PROMIS fat Z**			**PROMIS dep Z**		
Predictors	*Estimates*	*CI*	*p*	*Estimates*	*CI*	*p*	*Estimates*	*CI*	*p*	*Estimates*	*CI*	*p*	*Estimates*	*CI*	*p*
(Intercept)	1.78	1.52 – 2.04	**<0.001**	2.11	1.78 – 2.44	**<0.001**	-2.12	-2.45 – -1.78	**<0.001**	-3.42	-3.81 – -3.03	**<0.001**	-3.73	-4.14 – -3.33	**<0.001**
group [intervention]	0.03	-0.01 – 0.07	0.113	0.07	0.03 – 0.12	**0.003**	0.05	-0.00 – 0.09	0.051	0.07	0.02 – 0.12	**0.007**	0.08	0.02 – 0.13	**0.005**
gender [male]	0.02	-0.02 – 0.05	0.450	-0.01	-0.06 – 0.04	0.647	0.01	-0.04 – 0.06	0.702	-0.06	-0.11 – -0.01	**0.022**	0.02	-0.03 – 0.08	0.444
age	-0.00	-0.01 – -0.00	**<0.001**	-0.01	-0.01 – -0.01	**<0.001**	-0.01	-0.01 – -0.01	**<0.001**	0.00	-0.00 – 0.00	0.118	-0.00	-0.00 – 0.00	0.180
mobilisation [within 12 hours after surgery]	0.29	0.05 – 0.52	**0.016**	0.39	0.10 – 0.68	**0.009**	0.63	0.34 – 0.91	**<0.001**	0.43	0.12 – 0.74	**0.007**	0.46	0.14 – 0.79	**0.004**
mobilisation [within 24 hours after surgery]	0.23	-0.01 – 0.46	0.056	0.35	0.06 – 0.65	**0.018**	0.54	0.26 – 0.83	**<0.001**	0.32	0.01 – 0.63	**0.046**	0.39	0.06 – 0.71	**0.019**
mobilisation [within 48	0.12	-0.15 – 0.39	0.382	0.23	-0.11 – 0.58	0.187	0.39	0.05 – 0.72	**0.023**	0.24	-0.13 – 0.60	0.206	0.15	-0.23 – 0.53	0.429

© 2023 Steinbeck V et al. *JAMA Network Open.*

hours after surgery]															
mobilisation [within 6 hours after surgery]	0.32	0.09 – 0.55	**0.007**	0.45	0.16 – 0.74	**0.002**	0.69	0.41 – 0.97	**<0.001**	0.43	0.12 – 0.74	**0.007**	0.46	0.14 – 0.78	**0.004**
EQ total admission	-3.11	-3.18 – -3.03	**<0.001**												
EQVAS admission				-0.03	-0.03 – -0.03	**<0.001**									
HOOS KOOS admission							0.04	0.04 – 0.04	**<0.001**						
PROMIS fat admission										0.06	0.06 – 0.06	**<0.001**			
PROMIS dep admission													0.07	0.06 – 0.07	**<0.001**
**Random Effects**															
σ^2	0.35			0.56			0.52			0.63			0.67		
τ_{00}	0.00 hospital			0.01 hospital			0.02 hospital			0.02 hospital			0.01 hospital		
ICC	0.01			0.01			0.03			0.03			0.01		
N	9 hospital			9 hospital			9 hospital			9 hospital			9 hospital		
Observations	3,697			3,697			3,697			3,697			3,697		

© 2023 Steinbeck V et al. *JAMA Network Open.*

Marginal R^2 / Conditional R^2	0.646 / 0.650	0.437 / 0.443	0.461 / 0.477	0.342 / 0.362	0.318 / 0.326

Knee replacement patients

	EQ total Z			EQ-VAS Z			KOOS Z			PROMIS fat Z			PROMIS dep Z		
Predictors	*Estimates*	*CI*	*p*	*Estimates*	*CI*	*p*	*Estimates*	*CI*	*p*	*Estimates*	*CI*	*p*	*Estimates*	*CI*	*p*
(Intercept)	1.16	0.83 – 1.50	**<0.001**	1.57	1.19 – 1.95	**<0.001**	-2.36	-2.80 – -1.93	**<0.001**	-3.52	-3.97 – -3.08	**<0.001**	-3.46	-3.92 – -2.99	**<0.001**
group [intervention]	0.03	-0.02 – 0.08	0.248	0.08	0.02 – 0.14	**0.005**	0.06	-0.00 – 0.12	0.057	0.08	0.02 – 0.14	**0.006**	0.03	-0.03 – 0.09	0.313
gender [male]	0.04	-0.01 – 0.09	0.096	0.06	0.00 – 0.11	**0.046**	0.07	0.01 – 0.13	**0.016**	0.03	-0.03 – 0.08	0.400	0.10	0.04 – 0.16	**0.001**
age	0.00	0.00 – 0.01	**0.012**	-0.00	-0.00 – 0.00	0.308	0.00	0.00 – 0.01	**0.007**	0.01	0.00 – 0.01	**<0.001**	0.00	-0.00 – 0.00	0.514
mobilisation [within 12 hours after surgery]	0.43	0.15 – 0.71	**0.003**	0.33	0.01 – 0.65	**0.042**	0.23	-0.11 – 0.57	0.187	0.34	0.00 – 0.67	**0.047**	0.17	-0.17 – 0.52	0.321
mobilisation [within 24 hours after surgery]	0.35	0.07 – 0.62	**0.015**	0.30	-0.02 – 0.62	0.064	0.13	-0.21 – 0.47	0.460	0.28	-0.06 – 0.61	0.102	0.13	-0.21 – 0.48	0.450
mobilisation [within 48 hours after surgery]	0.18	-0.13 – 0.49	0.253	0.13	-0.23 – 0.49	0.478	0.09	-0.29 – 0.47	0.649	0.18	-0.19 – 0.55	0.341	-0.09	-0.47 – 0.29	0.640

© 2023 Steinbeck V et al. *JAMA Network Open.*

mobilisation [within 6 hours after surgery]	0.41	0.13 – 0.69	**0.004**	0.35	0.03 – 0.67	**0.031**	0.28	-0.06 – 0.63	0.105	0.32	-0.01 – 0.65	0.057	0.17	-0.17 – 0.51	0.335
EQ total admission	-2.92	-3.02 – -2.82	**<0.001**												
EQVAS admission				-0.03	-0.03 – -0.03	**<0.001**									
HOOS KOOS admission							0.04	0.04 – 0.04	**<0.001**						
PROMIS fat admission										0.06	0.05 – 0.06	**<0.001**			
PROMIS dep admission													0.06	0.06 – 0.07	**<0.001**
**Random Effects**															
σ^2	0.47			0.62			0.71			0.66			0.71		
τ_{00}	0.00 hospital			0.00 hospital			0.02 hospital			0.02 hospital			0.01 hospital		
ICC	0.01			0.01			0.03			0.03			0.01		
N	9 hospital			9 hospital			9 hospital			9 hospital			9 hospital		
Observations	3,110			3,110			3,110			3,110			3,110		
Marginal R^2 / Conditional R^2	0.529 / 0.534			0.380 / 0.384			0.267 / 0.289			0.303 / 0.326			0.272 / 0.282		

© 2023 Steinbeck V et al. *JAMA Network Open.*

eTable 12. Mixed Effects Models With PROM-Score Change Z-Scores as Outcomes, Controlling for Gender, Age, Mobilization and Admission PROM Scores as Fixed Effects and Hospital as Random Intercept (Compliance Corrected Study Population)

Hip replacement patients

	**EQ total Z**			**EQ-VAS Z**			**HOOS Z**			**PROMIS fat Z**			**PROMIS dep Z**		
Predictors	*Estimates*	*CI*	*p*	*Estimates*	*CI*	*p*	*Estimates*	*CI*	*p*	*Estimates*	*CI*	*p*	*Estimates*	*CI*	*p*
(Intercept)	1.78	1.51 – 2.05	**<0.001**	2.12	1.77 – 2.46	**<0.001**	-2.12	-2.47 – -1.78	**<0.001**	-3.43	-3.83 – -3.02	**<0.001**	-3.76	-4.18 – -3.33	**<0.001**
group [intervention]	0.03	-0.01 – 0.07	0.121	0.08	0.03 – 0.13	**0.002**	0.05	0.00 – 0.10	**0.043**	0.07	0.02 – 0.13	**0.007**	0.07	0.02 – 0.13	**0.010**
gender [male]	0.01	-0.03 – 0.05	0.575	-0.02	-0.07 – 0.03	0.492	0.01	-0.04 – 0.06	0.808	-0.06	-0.11 – -0.00	**0.041**	0.02	-0.03 – 0.08	0.399
age	-0.00	-0.01 – -0.00	**<0.001**	-0.01	-0.01 – -0.01	**<0.001**	-0.01	-0.01 – -0.01	**<0.001**	0.00	-0.00 – 0.00	0.133	-0.00	-0.00 – 0.00	0.227
mobilisation [within 12 hours after surgery]	0.31	0.07 – 0.55	**0.012**	0.39	0.08 – 0.69	**0.012**	0.65	0.35 – 0.94	**<0.001**	0.44	0.12 – 0.76	**0.007**	0.47	0.14 – 0.80	**0.005**
mobilisation [within 24 hours after surgery]	0.25	0.01 – 0.49	**0.042**	0.35	0.05 – 0.65	**0.023**	0.56	0.27 – 0.86	**<0.001**	0.33	0.01 – 0.66	**0.043**	0.39	0.06 – 0.73	**0.021**
mobilisation [within 48	0.16	-0.13 – 0.44	0.277	0.26	-0.10 – 0.62	0.156	0.42	0.07 – 0.77	**0.017**	0.28	-0.11 – 0.66	0.156	0.19	-0.20 – 0.58	0.338

© 2023 Steinbeck V et al. *JAMA Network Open.*

hours after surgery]															
mobilisation [within 6 hours after surgery]	0.33	0.10 – 0.57	**0.006**	0.45	0.15 – 0.75	**0.003**	0.71	0.41 – 1.00	**<0.001**	0.43	0.11 – 0.75	**0.009**	0.46	0.13 – 0.79	**0.006**
EQ total admission	-3.12	-3.20 – -3.04	**<0.001**												
EQVAS admission				-0.03	-0.03 – -0.03	**<0.001**									
HOOS KOOS admission							0.04	0.04 – 0.04	**<0.001**						
PROMIS fat admission										0.06	0.05 – 0.06	**<0.001**			
PROMIS dep admission													0.07	0.06 – 0.07	**<0.001**
**Random Effects**															
σ^2	0.36			0.57			0.54			0.65			0.69		
τ_{00}	0.00 hospital			0.01 hospital			0.02 hospital			0.02 hospital			0.01 hospital		
ICC	0.01			0.01			0.03			0.03			0.01		
N	9 hospital			9 hospital			9 hospital			9 hospital			9 hospital		
Observations	3,492			3,492			3,492			3,492			3,492		

© 2023 Steinbeck V et al. *JAMA Network Open.*

Marginal R^2 / Conditional R^2	0.639 / 0.643	0.431 / 0.438	0.453 / 0.469	0.333 / 0.353	0.312 / 0.322

Knee replacement patients

	EQ total Z			EQ-VAS Z			KOOS Z			PROMIS fat Z			PROMIS dep Z		
Predictors	*Estimates*	*CI*	*p*	*Estimates*	*CI*	*p*	*Estimates*	*CI*	*p*	*Estimates*	*CI*	*p*	*Estimates*	*CI*	*p*
(Intercept)	1.16	0.83 – 1.50	**<0.001**	1.57	1.19 – 1.95	**<0.001**	-2.36	-2.80 – -1.93	**<0.001**	-3.52	-3.97 – -3.08	**<0.001**	-3.46	-3.92 – -2.99	**<0.001**
group [intervention]	0.03	-0.02 – 0.08	0.248	0.08	0.02 – 0.14	**0.005**	0.06	-0.00 – 0.12	0.057	0.08	0.02 – 0.14	**0.006**	0.03	-0.03 – 0.09	0.313
gender [male]	0.04	-0.01 – 0.09	0.096	0.06	0.00 – 0.11	**0.046**	0.07	0.01 – 0.13	**0.016**	0.03	-0.03 – 0.08	0.400	0.10	0.04 – 0.16	**0.001**
age	0.00	0.00 – 0.01	**0.012**	-0.00	-0.00 – 0.00	0.308	0.00	0.00 – 0.01	**0.007**	0.01	0.00 – 0.01	**<0.001**	0.00	-0.00 – 0.00	0.514
mobilisation [within 12 hours after surgery]	0.43	0.15 – 0.71	**0.003**	0.33	0.01 – 0.65	**0.042**	0.23	-0.11 – 0.57	0.187	0.34	0.00 – 0.67	**0.047**	0.17	-0.17 – 0.52	0.321
mobilisation [within 24 hours after surgery]	0.35	0.07 – 0.62	**0.015**	0.30	-0.02 – 0.62	0.064	0.13	-0.21 – 0.47	0.460	0.28	-0.06 – 0.61	0.102	0.13	-0.21 – 0.48	0.450
mobilisation [within 48 hours after surgery]	0.18	-0.13 – 0.49	0.253	0.13	-0.23 – 0.49	0.478	0.09	-0.29 – 0.47	0.649	0.18	-0.19 – 0.55	0.341	-0.09	-0.47 – 0.29	0.640

© 2023 Steinbeck V et al. *JAMA Network Open.*

mobilisation [within 6 hours after surgery]	0.41	0.13 – 0.69	**0.004**	0.35	0.03 – 0.67	**0.031**	0.28	-0.06 – 0.63	0.105	0.32	-0.01 – 0.65	0.057	0.17	-0.17 – 0.51	0.335
EQ total admission	-2.92	-3.02 – -2.82	**<0.001**												
EQVAS admission				-0.03	-0.03 – -0.03	**<0.001**									
HOOS KOOS admission							0.04	0.04 – 0.04	**<0.001**						
PROMIS fat admission										0.06	0.05 – 0.06	**<0.001**			
PROMIS dep admission													0.06	0.06 – 0.07	**<0.001**
**Random Effects**															
σ^2	0.47			0.62			0.71			0.66			0.71		
τ_{00}	0.00 hospital			0.00 hospital			0.02 hospital			0.02 hospital			0.01 hospital		
ICC	0.01			0.01			0.03			0.03			0.01		
N	9 hospital			9 hospital			9 hospital			9 hospital			9 hospital		
Observations	3,110			3,110			3,110			3,110			3,110		
Marginal R^2 / Conditional R^2	0.529 / 0.534			0.380 / 0.384			0.267 / 0.289			0.303 / 0.326			0.272 / 0.282		

© 2023 Steinbeck V et al. *JAMA Network Open.*
