# Supplementary material for: Electronic Patient-Reported Outcome Monitoring to Improve Quality of Life After Joint Replacement: Secondary Analysis of a Randomized Clinical Trial
Source: JAMA Netw Open. 2023 Sep 1;6(9):e2331301. doi: 10.1001/jamanetworkopen.2023.31301 (PMC10474554; doi:10.1001/jamanetworkopen.2023.31301)
Supplement: Supplement 3. — Data Sharing Statement [file jamanetwopen-e2331301-s003.pdf]

Data Sharing Statement

Steinbeck. Electronic Patient-Reported Outcome Monitoring to Improve Quality of Life After Joint Replacement. *JAMA Netw Open*. Published September 01, 2023. doi:10.1001/jamanetworkopen.2023.31301

Data
Data available: No

Additional Information
Explanation for why data not available: It is not allowed to make the data accessible due to the German data protection law and the data protection agreements within the trial.
